# Design, Synthesis, and Anti-Hepatic Fibrosis Evaluation of Cordycepin Derivatives

**DOI:** 10.3390/molecules31020264

**Published:** 2026-01-12

**Authors:** Wenfang Pan, Siqi Liu, Yuanchen Zhong, Bixi Tang, Yi Zang, Yuanchao Xie

**Affiliations:** 1 Lingang Laboratory, Shanghai 200031, China; s20230007panwenfang@lglab.ac.cn (W.P.); liusiqi@simm.ac.cn (S.L.); zhongych2024@shanghaitech.edu.cn (Y.Z.); phoenixabc@foxmail.com (B.T.); 2 School of Physical Science and Technology, ShanghaiTech University, Shanghai 201210, China; 3 School of Chinese Materia Medica, Nanjing University of Chinese Medicine, Nanjing 210023, China; 4 Stake Key Laboratory of Chemical Biology, Shanghai Institute of Materia Medica, Chinese Academy of Sciences, Shanghai 201203, China

**Keywords:** cordycepin, hepatic fibrosis, HSCs activation, α-SMA, AMPK

## Abstract

Activation of hepatic stellate cells (HSCs) featuring upregulated expression of α-smooth muscle actin (α-SMA) is recognized as a key driver for hepatic fibrosis, which provides a promising strategy for seeking anti-liver fibrogenic agents via suppressing the activation event. In this study, we designed and synthesized twenty-eight cordycepin derivatives through structural modifications at the C2 position and the C6-NH_2_ group of the purine moiety. These compounds were screened for their inhibitory effects on HSC activation by detecting the mRNA expression of α-SMA using quantitative real-time polymerase chain reaction (qPCR) in the LX-2 cell model. Most compounds displayed inhibitory activity comparable to cordycepin, with compound **3a** bearing a C2-chloro and a N6-methyl-N6-(2-chlorobenzyl) substituent, demonstrating enhanced in vitro anti-fibrotic effect. This compound was able to dose-dependently downregulate α-SMA and collagen-I at both mRNA and protein levels, inhibited LX-2 cell migration, and exhibited improved metabolic stability in liver microsomes. The Western blotting result also indicated that **3a** could activate the AMPK signaling pathway. Overall, these results suggest **3a** may serve as a lead compound for further investigation.

## 1. Introduction

Hepatic fibrosis is a dysregulated wound-healing response following persistent liver tissue damage and chronic inflammation disorder [1]. It is characterized by excessive fibrillar extracellular matrix (ECM) deposition and fibrotic scar formation, resulting in liver distorted morphology and dysfunction [2,3,4]. Hepatic fibrosis almost exists in all chronic liver diseases (CLDs), and acts as the key pathology for the evolution of CLDs to cirrhosis or hepatocellular carcinoma with high mortality, posing a severe threat to patient health and life [4,5]. However, current therapeutic methods for hepatic fibrosis are primarily targeted at liver diseases rather than the fibrosis itself, and the approved agents are mainly indicated for specific conditions such as fibrosis associated with hepatitis virus infection and metabolic-associated steatohepatitis (MASH), with anti-fibrotic effects being secondary [6,7]. Thus, the direct anti-hepatic fibrosis therapy presents an unmet medical need.

The pathogenesis of liver fibrosis is intricate, involving a multitude of biological processes, among which the activation of hepatic stellate cells (HSCs) is a pivotal event [8]. Upon activation, HSCs transdifferentiate into myofibroblasts, acquiring a proliferative, migratory, and contractile phenotype [9]. These activated cells are characterized by the marked upregulation of the fibrotic marker α-smooth muscle actin (α-SMA) and the excessive synthesis and deposition of ECM components, particularly type I collagen (collagen I) [10]. Recent studies have demonstrated that preventing excessive activation of HSCs is beneficial in suppressing the progression of liver fibrosis, and represents a promising therapeutic strategy for the treatment of liver fibrosis [8,11].

**Figure 1:**
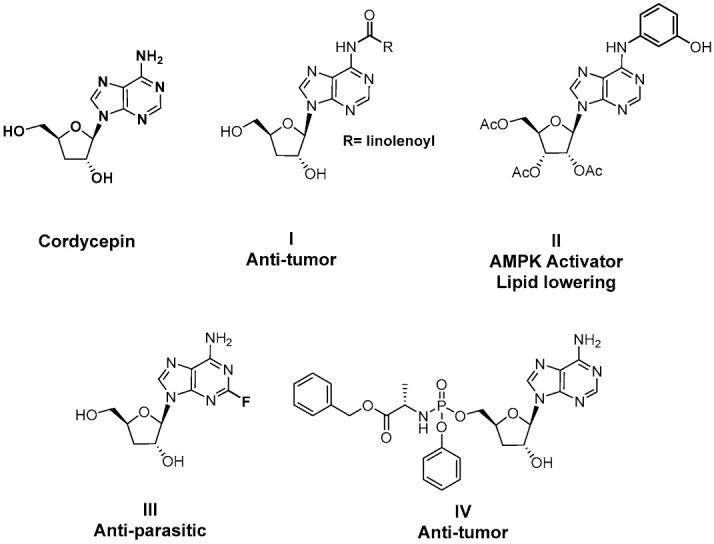
Structure of representative cordycepin derivatives from previous reports.

Cordycepin, an adenosine nucleoside analog that lacks a 3′-hydroxyl group at the ribose, is the main bioactive component of the traditional Chinese herbal medicine Cordyceps militaris [12]. This natural product has been reported to exhibit multiple biological activities, including anti-inflammation, anti-tumor, antiviral, and trypanocidal effects [13,14,15,16,17,18,19,20], and has received extensive attention as a lead compound for drug discovery (Figure 1) [21,22,23,24,25]. Recent studies have also revealed that cordycepin possesses hepatoprotective properties against a range of liver disorders, such as acute liver injury, nonalcoholic steatohepatitis, and diabetes mellitus-associated hepatic fibrosis [26,27,28]. Notably, its anti-fibrotic activity is linked to the suppression of HSCs activation. A recent study by Zhu et al. in 2024 reported that cordycepin exhibited dose-dependent repressive effects on HSC proliferation, migration, and pro-fibrotic function in TGF-β-activated HSC lines. In the diethyl 1,4-dihydro-2,4,6-trimethyl-3,5-pyridinedicarboxylate (DDC)-induced mouse model, cordycepin at an oral dose of 100–200 mg/kg for 4 weeks alleviated hepatocyte injury and suppressed HSC activation [29]. Cordycepin has also been reported to inhibit HSC activation in a diabetic mouse model [28].

**Figure 2:**
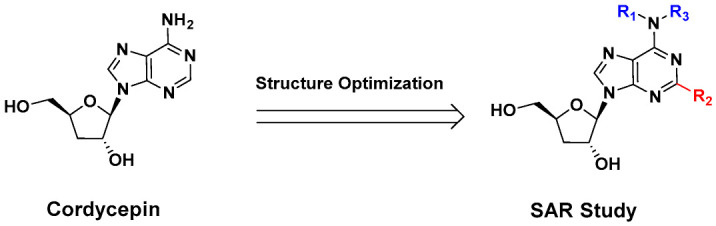
Structure optimization strategy in this study.

Despite its anti-fibrotic potential, the therapeutic efficacy of cordycepin is modest, and research on the structure–activity relationship (SAR) for its derivatives is lacking. Herein, we designed and synthesized a series of cordycepin derivatives by incorporating alkyl groups to the C6-NH_2_ and/or small substituents (halogen and hydroxyl group) at the C2 position (Figure 2). These compounds were screened in the human hepatic stellate cell line LX-2 for the activity of inhibiting the mRNA expression of the fibrotic marker α-SMA [30]. The C2, N6-dual substituted cordycepin derivatives exhibited favorable α-SMA-inhibiting activities, and a preliminary investigation on the mechanism of action was conducted.

## 2. Results and Discussion

### 2.1. Chemistry

**Scheme 1:**
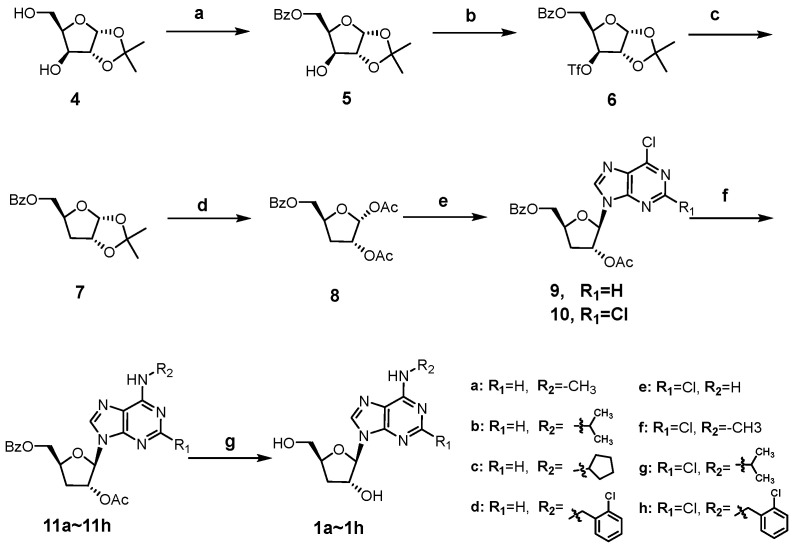
Synthesis of compounds **1a**~**1h**. Reagents and conditions: (**a**) BzCl, pyridine, −20 °C; (**b**) Tf_2_O, pyridine, DCM, −15 °C; (**c**) tetrabutylammonium borohydride, toluene, 80 °C; (**d**) Ac_2_O, conc. H_2_SO_4_, AcOH; (**e**) N, O-bis(trimethylsilyl)acetamide, TMSOTf, 6-chloropurine for **9**, 2,6-dichloropurine for **10**, ACN, 80 °C; (**f**) alkyl amines, TEA, ethanol, 60 °C; (**g**) 7N NH_3_/MeOH, 50 °C.

As shown in Scheme 1, the synthesis of cordycepin derivatives **1a**~**1h** begins with the commercially available 1,2-O-isopropy-lidene-α-D-xylofuranose (**4**). Selective benzoylation of the 5′-OH of **4** with benzoyl chloride furnished **5** [31], which was then trifluoromethanesulfonylated and subjected to reduction with tetrabutylammonium borohydride to yield the key 3′-deoxy intermediate **7** [32]. Treatment of **7** with acetic anhydride and conc. H_2_SO_4_ in acetic acid afforded the 1,2-diacetate intermediate **8** [33]. Vorbrüggen glycosylation of **8** with 6-chloropurine and 2,6-dichloropurine smoothly afforded the ester-protected nucleosides **9** and **10**, respectively [34]. The C6-chlorine of **9** and **10** were readily converted to alkylamino groups by SN_AR_ reactions with different amines (methylamine, isopropylamine, cyclopentylamine, and 2-chlorobenzylamine) under basic conditions. The obtained intermediates (**11a**~**11h**) were treated with methanolic ammonia to remove the protecting groups at C3′-OH and C5′-OH, giving the title compounds **1a**~**1h**.

**Scheme 2:**
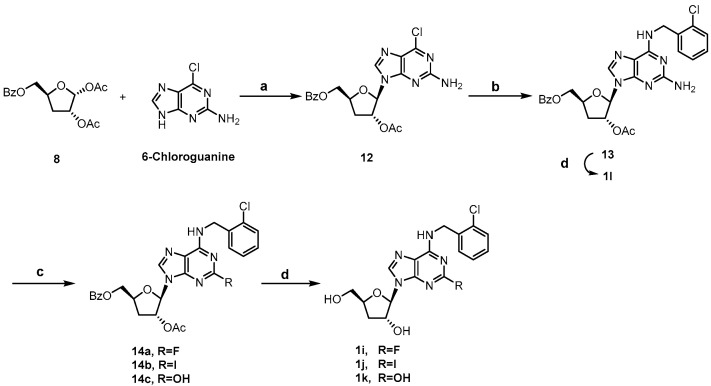
Synthesis of compounds **1i**~**1l**. Reagents and conditions: (**a**) N, O-bis(trimethylsilyl)acetamide, TMSOTf, ACN, 80 °C; (**b**) 2-chlorobenzylamine, TEA, ethanol, 60 °C; (**c**) i. tert-butyl nitrite, 70% pyridine hydrofluoride, −40 °C for **14a**; ii. I_2_, diiodomethane, cuprous iodide, tert-butyl nitrite, THF, 80 °C for **14b**; iii. tert-butyl nitrite, water: isopropanol (*v*:*v* = 1:1), 0 °C to rt for **14c**; (**d**) 7N NH_3_/MeOH, 50 °C.

Scheme 2 showed the synthesis of C2-substituted, N6-ortho-chlorobenzyl cordycepin derivatives **1i**~**1l**. Intermediate **13** was accessed using a similar method to that for **11** in Scheme 1, and deprotected to afford the target compound **1l**. The C2-amino group of intermediate **13** was diazotized using tert-butyl nitrite, followed by fluorination [35], iodination [36], and hydrolysis to give **14a**, **14b**, and **14c**, respectively. Removal of the protecting groups afforded compounds **1i**~**1k**.

**Scheme 3:**
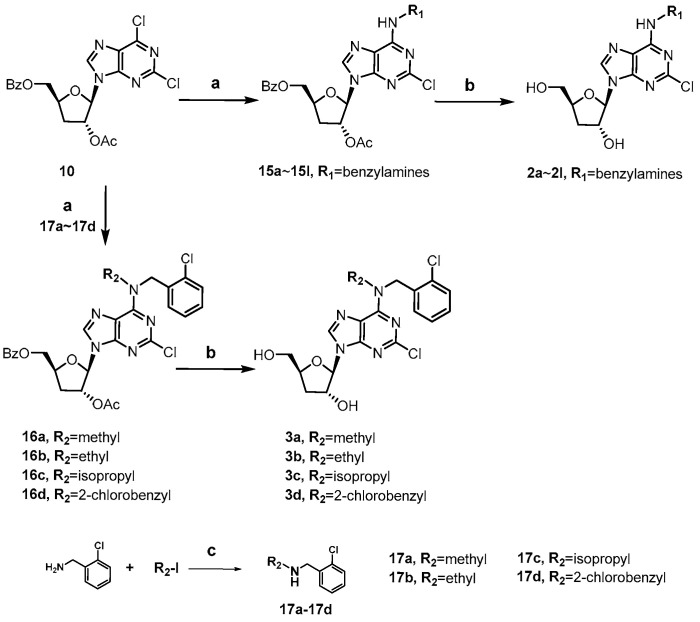
Synthesis of compounds **2a**~**2l** and **3a**~**3d**. Reagents and conditions: (**a**) various substituted benzylamines, TEA, ethanol, 60 °C; (**b**) 7N NH_3_/MeOH, 50 °C; (**c**) N,N-diisopropyl-ethylamine, ACN, 90 °C, iodomethane for **17a**, iodoethane for **17b**, 2-iodopropane for **17c**, 2-chlorobenzyl bromide for **17d**.

The synthesis of 2-chloro, N6-substituted cordycepin derivatives **2a**~**2l** and **3a**~**3d** was achieved in two steps from intermediate **10** as depicted in Scheme 3. The N6-substituted benzylamine derivatives **2a**~**2l** were obtained following the same approach employed for compounds **1e**~**1h**. Preparation of N,N-disubstituted derivatives **3a**~**3b** was synthesized analogously, with pre-formed secondary amines **17a**~**17d** in place of the primary benzylamines.

### 2.2. SAR Studies of Cordycepin Derivatives Inhibiting HSCs Activation

The overexpression of α-SMA is recognized as a key indicator of HSC activation and serves as a reliable biomarker for liver fibrosis [37]. Thus, the inhibitory activity against α-SMA expression could be used for assessing the anti-fibrotic potential. In this study, preliminary screening of these cordycepin derivatives was performed by evaluating the inhibition rates of α-SMA mRNA expression at concentrations of 5 μM and 10 μM in LX-2 hepatic stellate cells. LX-2 cells were treated with the test compounds and 10 ng/mL TGF-β for 24 h. Subsequently, α-SMA mRNA transcript levels were quantified by qPCR, and the percentage inhibition of α-SMA transcription was calculated. The structures of compounds and their α-SMA inhibiting activities are summarized in Table 1, Table 2 and Table 3.

Initially, we modified the C6-NH_2_ of cordycepin by incorporating four different alkyl groups (methyl, isopropyl, cyclopentyl, and ortho-chlorobenzyl) at this site, obtaining compounds **1a**~**1d**. As shown in Table 1, the four compounds did not exhibit any activity at the concentration of 5 μM, while cordycepin presented potent activity with 42.3% and 52.9% α-SMA mRNA inhibition rates at 5 μM and 10 μM, respectively. A similar result was observed for compound **1e**, a cordycepin analog with a C2-chlorine atom. It seemed that structural modifications at the C6-NH_2_ or the C2 position of the cordycepin nucleobase were not tolerated, and even minor alterations abolished the activity. Despite this, we proceeded to synthesize three compounds (**1f**~**1h**) bearing substituents at the two positions. Interestingly, they were found to be active in inhibiting α-SMA mRNA expression, particularly compound **1h** with a combination of C6-ortho-chlorobenzylamino and C2-chlorine groups. This compound was more potent than cordycepin at a concentration of 10 μM, with an inhibition rate of 62.3%. The result also indicated that the C6-benzylamino group (**1h**) favored the activity compared to the alkylamino groups (**1f** and **1g**). Along this line, another four compounds were synthesized by changing the C2-chlorine of **1h** to fluorine (**1i**), iodine (**1j**), hydroxyl group (**1k**), and amino group (**1l**). It was found that the two halogen-substituted compounds retained the α-SMA-inhibiting activities, but **1k** and **1l** exhibited obviously decreased activities with inhibition rates below 25% at 10 μM.

Subsequently, taking compound **1h** as the lead, we explored the SAR of the cordycepin derivatives, focusing on the N6-benzyl moiety, and the result is shown in Table 2. Removing the chloro atom on the benzene ring of **1h** from the ortho-site to meta- or para-site did not significantly affect the activity (**2a** and **2b**). Compounds **2c**~**2i** were designed by introducing various groups at the ortho position of the benzene ring. Except for the fluoro substitution, all the substituents were found to be well tolerated with α-SMA-inhibition rate ranging from 40.2% to 55.9% at 10 μM, indicating that the fluoro substituent with small size disfavored the activity. Relatively, the bromo (**2d**), trifluoromethyl (**2f**), and methoxyl (**2i**) substitutions led to better activities than the iodo (**2e**), cyano (**2g**), and hydroxyl (**2h**) substituted groups. The influence of substituents at the benzyl position on the activity was also investigated. It was found that increasing the steric hindrance by introducing a methyl group (**2j** and **2k**) or forming a cyclopropyl group (**2l**) at this site was positive for the activity.

Further structural optimizations of compound **1h** were performed by adding a second substituent at the N6 site, and four N,N-disubstituted cordycepin derivatives were synthesized. To our delight, the four compounds with an additional N6-methyl (**3a**), ethyl (**3b**), isopropyl (**3c**), and ortho-chlorobenzyl (**3d**) group demonstrated similar and enhanced α-SMA-inhibiting activities compared to **1h** or cordycepin, as illustrated in Table 3. Notably, the increase in the inhibition rate of these compounds from 5 μM to 10 μM was not evident. We did not choose a higher concentration for activity testing due to the concern of potential cytotoxicity that may impact the accuracy of test results.

### 2.3. α-SMA Inhibition Activity and In Vitro Metabolic Stability of ***3a*** and ***3b***

Upon the preliminary screening results, compounds **3a** and **3b** were selected for further evaluation. To quantitatively compare the anti-fibrotic potency of our synthetic analogs, we determined the half-maximal inhibitory concentration (IC_50_) of **3a** and cordycepin for the suppression of TGF-β-induced α-SMA mRNA expression in LX-2 cells. As shown in Table 4, analog **3a** exhibited promising α-SMA inhibitory activity (IC_50_ = 7.69 ± 5.1 µM), comparable to cordycepin (IC_50_ = 8.77 ± 2.3 µM). For compound **3b**, a reliable α-SMA IC_50_ was not determined due to its higher cytotoxicity in the LX-2 model (CC_50_ = 15.70 ± 2.4 µM), which limited the concentration range for a clear inhibitory dose–response. However, in a preliminary assessment using primary mouse hepatocytes (MPH), both **3a** and **3b** did not exert any toxicity at the concentration of 100 μM, differing from cordycepin, which reduced cell viability to 53%.

The metabolic stability of compounds **3a** and **3b** was assessed using human and mouse liver microsomes. As summarized in Table 5, compound **3a** displayed better metabolic stability with extended half-lives in both human liver microsome (T_1/2_, human = 35.57 min) and mouse liver microsome (T_1/2_, mouse = 26.50 ± 2.78 min) than cordycepin (T_1/2_, human and mouse = 27.21 ± 2.86 and 15.54 ± 0.20 min) and **3b** (T_1/2_, human and mouse= 18.32 ± 0.83 and 19.79 ± 2.28 min).

Cordycepin has been reported to be very susceptible to adenosine deaminase metabolism to be converted into 3′-deoxyinosine, which is considered a major cause for its short in vivo half-time [38,39]. For compounds **3a** and **3b**, though the C6-NH2 was modified, the alkylamino groups could undergo deamination or dealkylation, as evidenced by some previous studies [40]. In addition, the C-N glycosidic bond, mainly cleaved by nucleoside phosphorylases, is also a metabolic site [41]. Accordingly, it would be advantageous to use non-natural purine-like bases and the stable C–C glycosidic bonds for further structural modification of **3a** or cordycepin.

### 2.4. In Vitro Anti-Fibrotic Effects and AMPK Activating Effects of ***3a***

**Figure 3:**
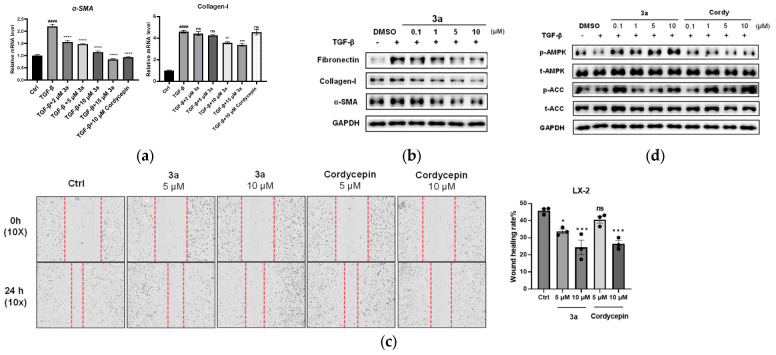
Compound **3a** inhibited TGF-β-induced LX-2 cells. (**a**). **3a** inhibited the mRNA expression of fibrogenic genes in LX-2 cells, including α-SMA, collagen-I; ** *p* < 0.01; *** *p* < 0.001, **** *p* < 0.0001, significantly different from the TGF-β treatment group; #### *p* < 0.0001, significantly different from the control group, ns: not significant. (**b**). **3a** effectively inhibited the α-SMA, collagen-I, and fibronectin expression on the protein level in LX-2 cells. (**c**) Cell scratch test to detect the effect of **3a** on the migration ability of LX-2 cells. * *p* < 0.05 vs. untreated control, *** *p* < 0.0005, ns: not significant. vs. the control. (**d**) Compound **3a** activates the AMPK signaling pathway.

Considering the cytotoxicity and metabolic stability, compound **3a** was selected for further evaluation in LX-2 cells for its anti-fibrotic effects by qPCR and Western blot assay. LX-2 cells were activated by 10 ng/mL TGF-β, and then treated with **3a** at different concentrations (2, 5, 10, and 15 μM) or cordycepin (10 μM). The qPCR analysis revealed that TGF-β significantly induced α-SMA mRNA expression, and this induction was dose-dependently suppressed by **3a** (Figure 3a). Similarly, TGF-β caused a marked increase in the collagen-I mRNA level, an indicator reflecting excessive ECM deposition in the fibrotic state. Compound **3a** could inhibit the collagen-I mRNA expression, but not as significantly as α-SMA mRNA (Figure 3a). The Western blot result showed that in TGF-β-activated LX-2 cells, **3a** treatment led to an obvious reduction in the expression of fibrotic proteins, including fibronectin, collagen I, and α-SMA at concentrations of 5 and 10 μM (Figure 3b), consistent with the qPCR results.

Activated hepatic stellate cells (HSCs) exhibit a migratory phenotype that contributes to fibrogenesis. To evaluate whether compound **3a** could inhibit this profibrotic behavior, we assessed its effect on HSC migration using a cell scratch test assay, with cordycepin as a positive control. The results demonstrated that **3a** significantly impeded LX-2 cell migration in a dose-dependent manner, with an effect greater than that of cordycepin (Figure 3c).

Adenosine monophosphate-activated protein kinase (AMPK) plays a crucial role in regulating energy metabolism that is closely related to the progression of liver fibrosis [42]. It has been reported that AMPK activation can inhibit HSC activation and reduce liver fibrosis [43]. Cordycepin has also been reported to ameliorate hepatic fibrosis in models of nonalcoholic steatohepatitis (NASH) through AMPK pathway activation [27]. We therefore preliminarily investigated whether our synthetic analog **3a** might interact with this key pathway, including cordycepin as a control. We found that treatment with **3a** effectively increased phosphorylation of AMPK and its downstream target ACC (Figure 3d). While this demonstrates an interaction with the AMPK pathway, the extent to which this specific interaction contributes to its anti-fibrotic activity remains to be determined.

## 3. Materials and Methods

### 3.1. Compound Synthesis and Characterization

All the chemical reagents and solvents from commercial sources were used without further purification unless otherwise stated. All reactions were performed under a nitrogen atmosphere using oven-dried glassware, and the solvents were dried before use. Reactions were monitored using analytic thin-layer chromatography on precoated silica plates (HSGF254, 0.2 mm ± 0.03 mm thickness). TLC plates were visualized with UV light (254 nm), 10% H_2_SO_4_-ethanol treatment, or iodine stain. Products were purified using a flash column with silica gel (300–400 mesh) and characterized by their NMR and MS spectra. 1H and 13C spectra were recorded on a Bruker Avance Neo 400 Spectrometer at 400 MHz and 101 MHz with Me4Si as internal standard, respectively. Low-resolution MS experiments were conducted with a mass detector (Agilent 1260 Infinity II), and high-resolution MS experiments were recorded on AB SCIEX TRIPLE QUAD 7500 + QTR.

#### 3.1.1. ((3aR,5R,6S,6aR)-6-Hydroxy-2,2-dimethyltetrahydrofuro[2,3-d][1,3]dioxol-5-yl)methyl Benzoate (**5**)

To a solution of 1,2-O-Isopropylidene-α-D-xylofuranose **4** (40.0 g, 210.5 mmol, 1 eq) in pyridine (400 mL) was added benzoyl chloride (32.4 g, 231.4 mmol, 1.1 eq) dropwise under a nitrogen atmosphere at −20 °C. The mixture was stirred for 2 h and then concentrated under reduced pressure. The residue was diluted with ethyl acetate and 1.0 M HCl aq, then the organic layer was separated, washed, dried, and concentrated. The crude product **5** was obtained and directly used in the next step without further purification.

#### 3.1.2. ((3aR,5S,6aR)-2,2-Dimethyltetrahydrofuro[2,3-d][1,3]dioxol-5-yl)methyl Benzoate (**7**)

To a solution of the crude intermediate **5** (210.5 mmol, 1 eq) in dichloromethane (500 mL) was added anhydrous pyridine (30.9 g, 315.31 mmol, 1.5 eq) and trifluoromethylsulfonic anhydride (66.0 g, 251.91 mmol, 1.2 eq) under a nitrogen atmosphere at −15 °C. After stirring for 1 h, the mixture was poured into ice water and extracted with dichloromethane. The organic phase was separated, washed with 1.0 M HCl aq and brine, and dried with Na_2_SO_4_. Removal of dichloromethane under vacuum gave triflate intermediate **6**, which was directly used in the next reaction without purification.

To a solution of yellow syrup **6** (210.5 mmol, 1 eq) in toluene (1000 mL) was added tetrabutylammonium borohydride (160 g, 600 mmol, 3 eq) portion-wise at room temperature. The mixture was stirred under a nitrogen atmosphere at 80 °C for 14 h. After completion of the reaction, the mixture was cooled to room temperature, then poured into ice water and stirred for two hours to release the gas. The residue was portioned between water and ethyl acetate, and the organic phase was washed, dried, and concentrated. The residue was purified by flash column to furnish **7** (20.0 g, 34.2% yield for three steps) as a colorless oil. ^1^H NMR (400 MHz, DMSO) δ 8.01–7.93 (m, 2H), 7.72–7.63 (m, 1H), 7.59–7.50 (m, 2H), 5.80 (d, *J* = 3.7 Hz, 1H), 4.78 (t, *J* = 4.2 Hz, 1H), 4.50–4.44 (m, 1H), 4.44–4.37 (m, 1H), 4.29 (dd, *J* = 11.7, 5.6 Hz, 1H), 2.06 (dd, *J* = 13.4, 4.4 Hz, 1H), 1.75 (ddd, *J* = 13.3, 10.8, 4.8 Hz, 1H), 1.41 (s, 3H), 1.25 (s, 3H). LRMS (ESI^+^) *m*/*z*: calcd for C_15_H_18_O_5_ [M + 23]^+^, 278.8; found, 301.7.

#### 3.1.3. (2R,3R,5S)-5-((Benzoyloxy)methyl)tetrahydrofuran-2,3-diyl Diacetate (**8**)

To a solution of **7** (13.0 g, 40.37 mmol, 1 eq) in a mixture of acetic anhydride (20 mL) and acetic acid (100 mL) was added conc. H_2_SO_4_ (1 mL) dropwise under a nitrogen atmosphere at 0 °C. The reaction was stirred at room temperature for 10 h. After condensation, the residue was poured into ethyl acetate and washed with saturated aqueous NaHCO_3_ solution, water, and brine. The organic phase was dried, concentrated, and purified to obtain **8** (11.4 g, 75.6% yield) as a colorless oil. ^1^H NMR (400 MHz, DMSO) δ 8.04–7.95 (m, 2H), 7.72–7.63 (m, 1H), 7.62–7.50 (m, 2H), 6.01 (s, 1H), 5.14 (dd, *J* = 4.6, 1.3 Hz, 1H), 4.64 (m, *J* = 9.2, 5.6, 2.9 Hz, 1H), 4.52 (dd, *J* = 12.1, 3.1 Hz, 1H), 4.34–4.22 (m, 1H), 2.24–2.19 (m, 2H), 2.06 (s, 3H), 1.86 (s, 3H). LRMS (ESI^+^) *m*/*z*: calcd for C_16_H_18_O_7_[M + 1]^+^, 322.3; found, 323.5.

#### 3.1.4. ((2S,4R,5R)-4-Acetoxy-5-(6-chloro-9H-purin-9-yl)tetrahydrofuran-2-yl)methyl Benzoate (**9**)

To a suspension of 6-chloropurine (2.5 g, 16.2 mmol, 1 eq) in acetonitrile (25 mL) was added N, O-Bis(trimethylsilyl)acetamide (6.7 g, 33.0 mmol, 2 eq). The mixture was stirred and refluxed under a nitrogen atmosphere at 80 °C for 1 h. After cooling to room temperature, a solution of **8** (5.3 g, 16.5 mmol, 1eq) dissolved in acetonitrile (25 mL) was added, followed by trimethylsilyl trifluoromethanesulfonate (7.3 g, 32.9 mmol, 2 eq). After refluxing for 2 h, the mixture was cooled to room temperature and partitioned between water and ethyl acetate. The organic phase was washed with aqueous saturated NaHCO_3_ and brine, dried over anhydrous Na_2_SO_4_, and evaporated in vacuum. The residue was purified through flash column to afford the product **9** (3.1 g, 45.2% yield) as white foam. ^1^H NMR (400 MHz, DMSO) δ 8.82 (s, 1H), 8.73 (s, 1H), 7.87–7.80 (m, 2H), 7.69–7.60 (m, 1H), 7.52–7.42 (m, 2H), 6.30 (d, *J* = 1.5 Hz, 1H), 5.84 (dt, *J* = 6.1, 1.5 Hz, 1H), 4.71 (m, *J* = 10.8, 5.5, 2.8 Hz, 1H), 4.61 (dd, *J* = 12.2, 2.9 Hz, 1H), 4.47 (dd, *J* = 12.2, 5.4 Hz, 1H), 2.81 (ddd, *J* = 14.0, 10.5, 6.2 Hz, 1H), 2.34 (ddd, *J* = 14.0, 5.7, 1.4 Hz, 1H), 2.11 (s, 3H). LRMS (ESI^+^) *m*/*z*: calcd for C_19_H_17_ClN_4_O_5_ [M + 1]^+^, 416.8; found, 418.0.

#### 3.1.5. ((2S,4R,5R)-4-Acetoxy-5-(2,6-dichloro-9H-purin-9-yl)tetrahydrofuran-2-yl)methyl Benzoate (**10**)

To a suspension of 2,6-dichloropurine (1.1 g, 5.82 mmol, 1 eq) in acetonitrile (20 mL) was added N, O-Bis(trimethylsilyl)acetamide (1.4 g, 6.90 mmol, 1.2 eq). The mixture was stirred and refluxed under a nitrogen atmosphere at 65 °C for 0.5 h. After cooling to room temperature, a solution of **8** (1.90 g, 5.90 mmol, 1eq) dissolved in acetonitrile (25 mL) was added, followed by trimethylsilyl trifluoromethanesulfonate (2.0 g, 9.01 mmol, 1.5 eq). After heating for 2 h, the mixture was cooled to room temperature and partitioned between water and ethyl acetate. The organic phase was washed with aqueous saturated NaHCO_3_ and brine, dried over anhydrous Na_2_SO_4_, and evaporated in vacuum. The residue was purified through flash column to afford the product **10** (1.8 g, 45.2% yield) as white foam. ^1^H NMR (400 MHz, DMSO) δ 8.85 (s, 1H), 7.87–7.80 (m, 2H), 7.69–7.60 (m, 1H), 7.54–7.43 (m, 2H), 6.27 (d, *J* = 1.4 Hz, 1H), 5.78 (dt, *J* = 5.9, 1.4 Hz, 1H), 4.71 (m, *J* = 10.8, 5.6, 2.8 Hz, 1H), 4.61 (dd, *J* = 12.2, 2.9 Hz, 1H), 4.49 (dd, *J* = 12.2, 5.6 Hz, 1H), 2.72 (ddd, *J* = 14.1, 10.5, 6.1 Hz, 1H), 2.34 (ddd, *J* = 14.0, 5.7, 1.4 Hz, 1H), 2.12 (s, 3H). C_19_H_16_Cl_2_N_4_O_5_ [M + 1]^+^, 451.3; found, 452.2.

#### 3.1.6. General Procedure A for the Synthesis of Compounds **11**, **13**, **15**, and **16**

To a solution of **9** or **10** (200 mg, 1 eq) and triethylamine (2 eq) in ethanol (2 mL) was added an appropriate amine (1.5 eq) under N_2_ at room temperature. The mixture was heated to 60 °C and stirred for 16 h. After completion of the reaction, the mixture was concentrated under reduced pressure to obtain the crude products **11**, **13**, **15**, and **16**, which were directly used in the next reaction.

#### 3.1.7. General Procedure B for the Synthesis of Compounds **1**, **2**, and **3**

A solution of the crude **11**, **13**, **15**, or **16** in 7N methanolic ammonia (5 mL) in a 15 mL sealed tube was stirred for 12 h at 50 °C. After cooling to room temperature, the mixture was concentrated under reduced pressure. The residue was purified through flash column chromatography to obtain **1**, **2**, and **3**.

#### 3.1.8. (2R,3R,5S)-5-(Hydroxymethyl)-2-(6-(methylamino)-9H-purin-9-yl)tetrahydrofuran-3-ol (**1a**)

A total of 97 mg, 71.5% yield over two steps: ^1^H NMR (400 MHz, DMSO) δ 8.34 (s, 1H), 8.23 (s, 1H), 7.75 (s, 1H), 5.88 (d, *J* = 2.5 Hz, 1H), 5.66 (d, *J* = 4.3 Hz, 1H), 5.15 (t, *J* = 5.6 Hz, 1H), 4.57 (tt, *J* = 5.9, 3.1 Hz, 1H), 4.35 (tt, *J* = 9.2, 3.8 Hz, 1H), 3.69 (ddd, *J* = 12.0, 5.2, 3.3 Hz, 1H), 3.51 (ddd, *J* = 12.0, 6.1, 3.9 Hz, 1H), 2.95 (s, 3H), 2.25 (ddd, *J* = 13.9, 8.6, 5.8 Hz, 1H), 1.92 (ddd, *J* = 13.0, 6.4, 3.2 Hz, 1H). ^13^C NMR (101 MHz, DMSO) δ 155.00, 152.45, 138.77, 90.78, 80.69, 74.63, 62.60, 34.06. HR-ESI-MS: [M + H]^+^ calcd for C_11_H_16_N_5_O_3_, 266.1253, found 266.1255.

#### 3.1.9. (2R,3R,5S)-5-(Hydroxymethyl)-2-(6-(isopropylamino)-9H-purin-9-yl)tetrahydrofuran-3-ol (**1b**)

A total of 70 mg, 50.5% yield over two steps: ^1^H NMR (400 MHz, DMSO) δ 8.34 (s, 1H), 8.19 (s, 1H), 7.55 (d, *J* = 8.4 Hz, 1H), 5.87 (d, *J* = 2.4 Hz, 1H), 5.65 (d, *J* = 4.3 Hz, 1H), 5.16 (t, *J* = 5.6 Hz, 1H), 4.58 (qd, *J* = 4.8, 2.9 Hz, 1H), 4.48–4.42 (m, 1H), 4.35 (ddt, *J* = 9.7, 6.8, 3.6 Hz, 1H), 3.69 (ddd, *J* = 12.2, 5.2, 3.4 Hz, 1H), 3.51 (ddd, *J* = 12.0, 6.1, 4.0 Hz, 1H), 2.25 (ddd, *J* = 13.8, 8.6, 5.7 Hz, 1H), 1.92 (ddd, *J* = 13.1, 6.4, 3.2 Hz, 1H), 1.21 (d, *J* = 6.4 Hz, 6H). ^13^C NMR (101 MHz, DMSO) δ 152.39, 138.68, 90.79, 80.68, 74.59, 62.61, 34.04, 22.31. HR-ESI-MS: [M + H]^+^ calcd for C_13_H_20_N_5_O_3_, 294.1566, found 294.1570.

#### 3.1.10. (2R,3R,5S)-2-(6-(Cyclopentylamino)-9H-purin-9-yl)-5-(hydroxymethyl)tetrahydrofuran-3-ol (**1c**)

A total of 83 mg, 59.1% yield over two steps: ^1^H NMR (400 MHz, DMSO) δ 8.35 (s, 1H), 8.19 (s, 1H), 7.69 (d, *J* = 7.7 Hz, 1H), 5.87 (d, *J* = 2.4 Hz, 1H), 5.65 (d, *J* = 4.2 Hz, 1H), 5.15 (t, *J* = 5.6 Hz, 1H), 4.57 (tt, *J* = 5.9, 3.1 Hz, 1H), 4.35 (ddt, *J* = 9.7, 6.7, 3.5 Hz, 1H), 3.69 (ddd, *J* = 12.0, 5.2, 3.3 Hz, 1H), 3.51 (ddd, *J* = 12.0, 6.1, 3.9 Hz, 1H), 2.25 (ddd, *J* = 13.9, 8.6, 5.7 Hz, 1H), 1.92 (ddt, *J* = 13.0, 6.2, 3.5 Hz, 3H), 1.75–1.65 (m, 2H), 1.61–1.47 (m, 4H). ^13^C NMR (101 MHz, DMSO) δ 152.32, 138.67, 90.78, 80.68, 74.59, 62.60, 34.04, 23.46. HR-ESI-MS: [M + H]^+^ calcd for C_15_H_22_N_5_O_3_, 320.1723, found 320.1732.

#### 3.1.11. (2R,3R,5S)-2-(6-((2-Chlorobenzyl)amino)-9H-purin-9-yl)-5-(hydroxymethyl)tetrahydrofuran-3-ol (**1d**)

A total of 71 mg, 42.9% yield over two steps: ^1^H NMR (400 MHz, DMSO) δ 8.42 (m, 2H), 8.20 (s, 1H), 7.44 (dd, *J* = 6.6, 3.0 Hz, 1H), 7.26 (t, *J* = 3.7 Hz, 4H), 5.90 (d, *J* = 2.4 Hz, 1H), 5.69 (d, *J* = 4.3 Hz, 1H), 5.14 (t, *J* = 5.6 Hz, 1H), 4.76 (s, 2H), 4.59 (s, 1H), 4.40–4.31 (m, 1H), 3.74–3.65 (m, 1H), 3.52 (ddd, *J* = 12.0, 6.0, 4.0 Hz, 1H), 2.26 (dt, *J* = 14.1, 7.8 Hz, 1H), 1.93 (ddd, *J* = 13.0, 6.3, 3.1 Hz, 1H). ^13^C NMR (101 MHz, DMSO) δ 152.37, 139.33, 131.70, 129.05, 128.28, 127.92, 127.09, 90.81, 80.80, 74.66, 62.55, 34.09. HR-ESI-MS: [M + H]^+^ calcd for C_17_H_19_ClN_5_O_3_, 376.1176, found 376.1199.

#### 3.1.12. (2R,3R,5S)-2-(6-Amino-2-chloro-9H-purin-9-yl)-5-(hydroxymethyl)tetrahydrofuran-3-ol (**1e**)

A total of 116 mg, 92.3% yield: ^1^H NMR (400 MHz, DMSO) δ 8.39 (s, 1H), 7.80 (s, 2H), 5.81 (d, *J* = 2.1 Hz, 1H), 5.68 (d, *J* = 4.1 Hz, 1H), 5.02 (t, *J* = 5.5 Hz, 1H), 4.52 (tt, *J* = 5.5, 2.8 Hz, 1H), 4.36 (ddt, *J* = 9.6, 6.7, 3.7 Hz, 1H), 3.70 (ddd, *J* = 12.1, 5.3, 3.4 Hz, 1H), 3.57–3.48 (m, 1H), 2.22 (ddd, *J* = 14.2, 9.2, 5.6 Hz, 1H), 1.90 (ddd, *J* = 13.1, 6.2, 2.8 Hz, 1H). ^13^C NMR (101 MHz, DMSO) δ 156.77, 152.97, 149.94, 139.43, 118.07, 90.61, 80.97, 74.82, 62.34, 33.86. HR-ESI-MS: [M + H]^+^ calcd for C_10_H_13_ClN_5_O_3_, 286.0707, found 286.0713.

#### 3.1.13. (2R,3R,5S)-2-(2-Chloro-6-(methylamino)-9H-purin-9-yl)-5-(hydroxymethyl)tetrahydrofuran-3-ol (**1f**)

A total of 73 mg, 55.5% yield over two steps: ^1^H NMR (400 MHz, DMSO) δ 8.38 (s, 1H), 8.25 (d, *J* = 5.0 Hz, 1H), 5.82 (d, *J* = 2.2 Hz, 1H), 5.69 (d, *J* = 4.1 Hz, 1H), 5.02 (t, *J* = 5.5 Hz, 1H), 4.54–4.49 (m, 1H), 4.36 (dq, *J* = 9.2, 4.1 Hz, 1H), 3.70 (dt, *J* = 11.9, 4.2 Hz, 1H), 3.53 (dt, *J* = 11.7, 4.7 Hz, 1H), 2.92 (d, *J* = 5.0 Hz, 3H), 2.22 (ddd, *J* = 14.1, 9.0, 5.6 Hz, 1H), 1.90 (ddd, *J* = 13.3, 6.3, 2.7 Hz, 1H). ^13^C NMR (101 MHz, DMSO) δ 155.49, 153.24, 148.82, 139.12, 118.57, 90.61, 81.00, 74.87, 62.31, 33.83, 27.18. HR-ESI-MS: [M + H]^+^ calcd for C_11_H_15_ClN_5_O_3_, 300.0863, found 300.0870.

#### 3.1.14. (2R,3R,5S)-2-(2-Chloro-6-(isopropylamino)-9H-purin-9-yl)-5-(hydroxymethyl)tetrahydrofuran-3-ol (**1g**)

A total of 79 mg, 54.9% yield over two steps: ^1^H NMR (400 MHz, DMSO) δ 8.38 (s, 1H), 8.14 (d, *J* = 8.3 Hz, 1H), 5.81 (d, *J* = 2.1 Hz, 1H), 5.68 (d, *J* = 4.1 Hz, 1H), 5.02 (t, *J* = 5.5 Hz, 1H), 4.51 (tt, *J* = 5.3, 2.6 Hz, 1H), 4.41–4.31 (m, 2H), 3.70 (ddd, *J* = 12.1, 5.3, 3.4 Hz, 1H), 3.53 (dt, *J* = 12.1, 4.7 Hz, 1H), 2.21 (ddd, *J* = 13.9, 9.0, 5.6 Hz, 1H), 1.89 (ddd, *J* = 13.1, 6.2, 2.7 Hz, 1H), 1.20 (d, *J* = 6.9 Hz, 6H). ^13^C NMR (101 MHz, DMSO) δ 171.49, 154.20, 153.21, 149.10, 139.01, 118.32, 90.61, 80.99, 74.84, 62.31, 41.74, 33.81, 22.04. HR-ESI-MS: [M + H]^+^ calcd for C_13_H_19_ClN_5_O_3_, 328.1176, found 328.1182.

#### 3.1.15. (2R,3R,5S)-2-(2-Chloro-6-((2-chlorobenzyl)amino)-9H-purin-9-yl)-5-(hydroxymethyl)tetrahydrofuran-3-ol (**1h**)

A total of 88 mg, 48.9% yield over two steps: ^1^H NMR (400 MHz, DMSO) δ 8.87 (d, *J* = 6.3 Hz, 1H), 8.46 (s, 1H), 7.46 (dd, *J* = 6.1, 3.3 Hz, 1H), 7.35–7.25 (m, 3H), 5.84 (s, 1H), 5.69 (d, *J* = 4.2 Hz, 1H), 5.03 (t, *J* = 5.6 Hz, 1H), 4.75–4.68 (m, 2H), 4.54 (s, 1H), 4.37 (dd, *J* = 9.7, 5.5 Hz, 1H), 3.71 (dt, *J* = 9.3, 4.3 Hz, 1H), 3.54 (dt, *J* = 11.2, 4.7 Hz, 1H), 2.22 (dd, *J* = 7.2, 6.3 Hz, 1H), 1.90 (dd, *J* = 13.5, 6.0 Hz, 1H). ^13^C NMR (101 MHz, DMSO) δ 154.98, 153.00, 149.36, 139.63, 136.00, 131.88, 129.16, 128.57, 128.31, 127.18, 118.58, 90.69, 81.06, 74.85, 62.26, 33.79. HR-ESI-MS: [M + H]^+^ calcd for C_17_H_18_Cl_2_N_5_O_3_, 410.0787, found 410.0806.

#### 3.1.16. ((2S,4R,5R)-4-Acetoxy-5-(2-amino-6-chloro-9H-purin-9-yl)tetrahydrofuran-2-yl)methyl Benzoate (**12**)

To a suspension of 6-chloroguanine (3.0 g, 17.6 mmol, 1 eq) in acetonitrile (15 mL) was added N,O-Bis(trimethylsilyl)acetamide (10.7 g, 52.7 mmol, 3 eq). The mixture was stirred and refluxed under a nitrogen atmosphere at 80 °C for 1 h. After cooling to room temperature, a solution of **8** (6.3 g, 19.5 mmol, 1.1 eq) dissolved in acetonitrile (15 mL) was added, followed by trimethylsilyl trifluoromethanesulfonate (7.8 g, 35.1 mmol, 2 eq). After refluxing for 2 h, the mixture was cooled to room temperature and partitioned between water and ethyl acetate. The organic phase was washed with aqueous saturated NaHCO_3_ and brine, dried over anhydrous Na_2_SO_4_, and evaporated in vacuum. The residue was purified through flash column to give the product **12** (6.1 g, 80.4% yield) as white foam. ^1^H NMR (400 MHz, DMSO) δ 8.27 (s, 1H), 7.93–7.82 (m, 2H), 7.63 (td, *J* = 7.3, 1.4 Hz, 1H), 7.48 (t, *J* = 7.8 Hz, 2H), 7.05 (s, 2H), 6.05 (d, *J* = 1.6 Hz, 1H), 5.76–5.69 (m, 1H), 4.68–4.54 (m, 2H), 4.42 (dd, *J* = 12.0, 5.5 Hz, 1H), 2.90 (ddd, *J* = 14.0, 10.3, 6.2 Hz, 1H), 2.33–2.23 (m, 1H), 2.10 (s, 3H). ESI-LR: [M + 1]^+^ calcd for C_19_H_18_ClN_5_O_5_, 431.8, found 432.7.

#### 3.1.17. ((2S,4R,5R)-4-Acetoxy-5-(2-amino-6-((2-chlorobenzyl) amino)-9H-purin-9-yl)tetrahydrofuran-2-yl)methyl Benzoate (**13**)

To a solution of **10** (1.6 g, 3.7 mmol, 1 eq) and triethylamine (1.1 g, 10.9 mmol, 3 eq) in ethanol (20 mL) was added 2-chlorobenzylamine (1.0 g, 7.1 mmol, 2 eq) at room temperature. The mixture was heated at 60 °C and stirred for 16 h. After completion of the reaction, the mixture was concentrated and purified with a flash column to give compound **11** (1.5 g, 75.2% yield) as white foam. ^1^H NMR (400 MHz, DMSO) δ 7.94–7.88 (m, 3H), 7.67–7.58 (m, 1H), 7.49 (t, *J* = 7.7 Hz, 2H), 7.45–7.41 (m, 1H), 7.32–7.20 (m, 3H), 6.02–5.96 (m, 3H), 5.69 (d, *J* = 6.1 Hz, 1H), 4.69 (s, 2H), 4.56 (dd, *J* = 11.9, 2.6 Hz, 1H), 4.39 (dd, *J* = 12.0, 5.6 Hz, 1H), 3.00–2.86 (m, 1H), 2.24 (dd, *J* = 14.0, 5.5 Hz, 1H), 2.10 (s, 3H). ESI-LR: [M + 1]^+^ calcd for C_26_H_25_ClN_6_O_5_, 536.2, found 537.0.

#### 3.1.18. ((2S,4R,5R)-4-Acetoxy-5-(6-((2-chlorobenzyl)amino)-2-fluoro-9H-purin-9-yl)tetrahydrofuran-2-yl)methyl Benzoate (**14a**)

To a solution of intermediate **13** (250 mg, 0.47 mmol, 1 eq) in 70% pyridine hydrofluoride (3 mL), tert-butyl nitrite (190 mg, 1.9 mmol, 4 eq) was added dropwise at −40 °C. After stirring for 1 h, the mixture was poured into ice water and extracted with ethyl acetate. The organic phase was washed, dried, and concentrated. The residue was purified with a flash column to give intermediate **14a** (185 mg, 72.9% yield) as a yellow syrup. ^1^H NMR (400 MHz, DMSO) δ 9.01 (t, *J* = 5.8 Hz, 1H), 8.33 (s, 1H), 7.91–7.82 (m, 2H), 7.63 (t, *J* = 7.4 Hz, 1H), 7.50–7.42 (m, 3H), 7.28 (p, *J* = 3.9, 3.5 Hz, 3H), 6.10 (d, *J* = 1.6 Hz, 1H), 5.72 (d, *J* = 6.1 Hz, 1H), 4.69 (m, 3H), 4.57 (dd, *J* = 12.2, 2.8 Hz, 1H), 4.44 (dd, *J* = 12.2, 5.3 Hz, 1H), 2.80 (ddd, *J* = 13.8, 10.4, 6.3 Hz, 1H), 2.30 (dt, *J* = 13.8, 6.1 Hz, 1H), 2.11 (s, 3H). ESI-LR: [M + 1]^+^ calcd for C_26_H_23_ClFN_5_O_5_, 539.1, found 540.0.

#### 3.1.19. ((2S,4R,5R)-4-Acetoxy-5-(6-((2-chlorobenzyl)amino)-2-iodo-9H-purin-9-yl) tetrahydrofuran-2-yl)methyl Benzoate (**14b**)

To a mixture of **13** (250 mg, 0.47 mmol, 1 eq), iodine (120 mg, 0.47 mmol, 1 eq), diiodomethane (131 mg, 0.47 mmol, 1 eq) and cuprous iodide (95 mg, 0.52 mmol, 1.1 eq) in tetrahydrofuran (5 mL) was added tert-butyl nitrite (190 mg, 1.88 mmol, 4 eq) dropwise. The mixture was heated to 80 °C and stirred for 4 h. After cooling to room temperature, the mixture was filtered and evaporated under reduced pressure. The residue was purified by flash column to afford intermediate **14b** (153 mg, 50.3% yield) as white foam. ^1^H NMR (400 MHz, DMSO) δ 8.94 (t, *J* = 6.1 Hz, 1H), 8.37 (s, 1H), 7.87–7.83 (m, 2H), 7.65–7.60 (m, 1H), 7.46 (td, *J* = 6.7, 5.6, 2.8 Hz, 3H), 7.28 (dt, *J* = 5.4, 3.2 Hz, 3H), 6.13 (d, *J* = 1.5 Hz, 1H), 5.73 (d, *J* = 6.0 Hz, 1H), 4.74–4.63 (m, 3H), 4.58 (dd, *J* = 12.1, 3.0 Hz, 1H), 4.44 (dd, *J* = 12.0, 5.3 Hz, 1H), 2.79 (ddd, *J* = 14.2, 10.5, 6.2 Hz, 1H), 2.33–2.25 (m, 1H), 2.11 (s, 3H). ESI-LR: [M + 1]+ calcd for C_26_H_23_ClIN_5_O_5_, 647.9, found 649.0.

#### 3.1.20. ((2S,4R,5R)-4-Acetoxy-5-(6-((2-chlorobenzyl)amino)-2-hydroxy-9H-purin-9-yl)tetrahydrofuran-2-yl)methyl Benzoate (**14c**)

To a solution of intermediate **13** (300 mg, 0.56 mmol, 1 eq) in a mixture of water (1 mL) and isopropanol (1 mL) was added tert-butyl nitrite (290 mg, 2.82 mmol, 5 eq) under a nitrogen atmosphere at 0 °C. The mixture was stirred for 6 h at room temperature, then partitioned between water and ethyl acetate. The organic phase was washed, dried, and concentrated. The residue was purified by flash column to give intermediate **14c** (120 mg, 39% yield) as white foam. ^1^H NMR (400 MHz, DMSO) δ 10.84 (s, 1H), 8.37 (s, 1H), 8.02 (s, 1H), 7.96–7.86 (m, 2H), 7.63 (t, *J* = 7.4 Hz, 1H), 7.53–7.42 (m, 3H), 7.27 (d, *J* = 5.1 Hz, 3H), 6.00 (s, 1H), 5.67 (d, *J* = 6.0 Hz, 1H), 4.72–4.68 (m, 2H), 4.66–4.52 (m, 2H), 4.41 (dd, *J* = 12.0, 5.4 Hz, 1H), 2.87–2.82 (m, 1H), 2.25 (dd, *J* = 14.0, 5.4 Hz, 1H), 2.10 (s, 3H). ESI-LR: [M + 1]^+^ calcd for C_26_H_24_ClN_5_O_6_, 538.0, found 538.9.

#### 3.1.21. (2R,3R,5S)-2-(6-((2-Chlorobenzyl)amino)-2-fluoro-9H-purin-9-yl)-5-(hydroxymethyl)tetrahydrofuran-3-ol (**1i**)

A total of 85 mg, 65.5% yield: ^1^H NMR (400 MHz, DMSO) δ 8.95 (t, *J* = 6.3 Hz, 1H), 8.43 (s, 1H), 7.46 (dt, *J* = 6.7, 3.0 Hz, 1H), 7.31–7.26 (m, 3H), 5.81 (d, *J* = 2.0 Hz, 1H), 5.70 (d, *J* = 4.2 Hz, 1H), 5.03 (t, *J* = 5.5 Hz, 1H), 4.70 (dd, *J* = 5.9, 2.6 Hz, 2H), 4.55 (d, *J* = 4.9 Hz, 1H), 4.41–4.32 (m, 1H), 3.71 (dt, *J* = 8.6, 4.1 Hz, 1H), 3.54 (dt, *J* = 12.2, 4.8 Hz, 1H), 2.23 (ddd, *J* = 14.2, 9.1, 5.7 Hz, 1H), 1.91 (ddd, *J* = 13.4, 6.2, 2.5 Hz, 1H). ^13^C NMR (101 MHz, DMSO) δ 156.06, 155.86, 149.55 (d, *J* = 20.3 Hz), 139.51, 135.94, 131.83, 129.17, 128.56, 128.16, 127.20, 117.90, 90.79, 81.06, 74.83, 62.30, 41.27, 33.83. HR-ESI-MS: [M + H]^+^ calcd for C_17_H_18_ClFN_5_O_3_, 394.1082, found 394.1089.

#### 3.1.22. (2R,3R,5S)-2-(6-((2-Chlorobenzyl)amino)-2-iodo-9H-purin-9-yl)-5-(hydroxymethyl)tetrahydrofuran-3-ol (**1j**)

A total of 62 mg, 53.9% yield: ^1^H NMR (400 MHz, DMSO) δ 8.88 (t, *J* = 6.3 Hz, 1H), 8.46 (s, 1H), 7.46 (dt, *J* = 7.2, 4.0 Hz, 1H), 7.29 (q, *J* = 5.8, 4.2 Hz, 2H), 5.84 (s, 1H), 5.69 (d, *J* = 4.1 Hz, 1H), 5.02 (t, *J* = 5.7 Hz, 1H), 4.79–4.64 (m, 2H), 4.55 (s, 1H), 4.38 (d, *J* = 10.5 Hz, 1H), 3.70 (dd, *J* = 10.9, 6.2 Hz, 1H), 3.54 (dt, *J* = 11.8, 4.7 Hz, 1H), 2.29–2.17 (m, 1H), 1.91 (q, *J* = 6.9, 6.2 Hz, 1H). ^13^C NMR (101 MHz, DMSO) δ 154.97, 153.00, 149.35, 139.63, 135.99, 131.87, 129.16, 128.57, 128.30, 127.18, 90.68, 81.05, 74.84, 62.26, 45.76, 33.78, 8.69. HR-ESI-MS: [M + H]^+^ calcd for C_17_H_18_ClIN_5_O_3_, 502.0143, found 502.0169.

#### 3.1.23. 6-((2-Chlorobenzyl)amino)-9-((2R,3R,5S)-3-hydroxy-5-(hydroxymethyl)tetra hydrofuran-2-yl)-9H-purin-2-ol (**1k**)

A total of 23 mg, 26.7% yield: ^1^H NMR (400 MHz, DMSO) δ 8.32 (s, 1H), 8.05 (s, 1H), 7.46 (dd, *J* = 6.8, 2.8 Hz, 1H), 7.39–7.22 (m, 3H), 5.71 (s, 1H), 5.61 (s, 1H), 4.70 (s, 1H), 4.48 (s, 1H), 4.38–4.27 (m, 1H), 3.67 (dd, *J* = 12.0, 3.2 Hz, 1H), 3.50 (dd, *J* = 12.1, 3.7 Hz, 1H), 2.21 (dt, *J* = 13.6, 7.0 Hz, 1H), 1.96–1.83 (m, 1H). ^13^C NMR (101 MHz, DMSO) δ 136.52, 131.77, 129.14, 128.45, 128.06, 127.21, 119.42, 90.60, 80.43, 74.43, 62.56, 34.01. HR-ESI-MS: [M + H]^+^ calcd for C_17_H_19_ClN_5_O_4_, 392.1126, found 392.1137.3.1.24.

#### 3.1.24. (2R,3R,5S)-2-(2-Amino-6-((2-chlorobenzyl)amino)-9H-purin-9-yl)-5-(hydroxymethyl)tetrahydrofuran-3-ol (**1l**)

A total of 44 mg, 29.7% yield: ^1^H NMR (400 MHz, DMSO) δ 7.96 (s, 1H), 7.84–7.79 (m, 1H), 7.46–7.39 (m, 1H), 7.33–7.21 (m, 3H), 5.86 (s, 2H), 5.72 (d, *J* = 2.6 Hz, 1H), 5.55 (d, *J* = 4.3 Hz, 1H), 5.11 (t, *J* = 5.6 Hz, 1H), 4.70 (s, 2H), 4.51 (d, *J* = 5.4 Hz, 1H), 4.30 (ddt, *J* = 10.2, 7.7, 3.9 Hz, 1H), 3.65 (dt, *J* = 12.7, 4.1 Hz, 1H), 3.55–3.45 (m, 1H), 2.24 (ddd, *J* = 13.8, 8.4, 5.9 Hz, 1H), 1.91 (ddd, *J* = 13.0, 6.5, 3.4 Hz, 1H). ^13^C NMR (101 MHz, DMSO) δ 160.58, 155.32, 137.82, 136.08, 132.12, 129.42, 128.63, 128.52, 127.52, 113.92, 90.48, 80.69, 74.93, 63.30, 34.97. HR-ESI-MS: [M + H]^+^ calcd for C_17_H_20_ClN_6_O_3_, 391.1285, found 391.1309.

#### 3.1.25. (2R,3R,5S)-2-(2-Chloro-6-((3-chlorobenzyl)amino)-9H-purin-9-yl)-5-(hydroxymethyl)tetrahydrofuran-3-ol (**2a**)

A total of 92 mg, 51.1% yield over two steps: ^1^H NMR (400 MHz, DMSO) δ 8.91 (t, *J* = 6.3 Hz, 1H), 8.44 (s, 1H), 7.41–7.38 (m, 1H), 7.38–7.27 (m, 3H), 5.83 (d, *J* = 2.0 Hz, 1H), 5.68 (d, *J* = 4.1 Hz, 1H), 5.02 (t, *J* = 5.5 Hz, 1H), 4.64 (t, *J* = 5.6 Hz, 2H), 4.53 (s, 1H), 4.36 (ddt, *J* = 9.5, 6.6, 3.5 Hz, 1H), 3.70 (ddd, *J* = 12.2, 5.3, 3.4 Hz, 1H), 3.58–3.48 (m, 1H), 2.22 (ddd, *J* = 14.0, 9.1, 5.5 Hz, 1H), 1.89 (ddd, *J* = 13.1, 6.1, 2.7 Hz, 1H). ^13^C NMR (101 MHz, DMSO) δ 154.78, 152.98, 149.31, 141.82, 139.57, 132.92, 130.22, 127.17, 126.83, 126.02, 118.50, 90.69, 81.06, 74.86, 62.24, 42.66, 33.75. HR-ESI-MS: [M + H]^+^ calcd for C_17_H_18_Cl_2_N_5_O_3_, 410.0787, found 410.0796.

#### 3.1.26. (2R,3R,5S)-2-(2-Chloro-6-((4-chlorobenzyl)amino)-9H-purin-9-yl)-5-(hydroxymethyl)tetrahydrofuran-3-ol (**2b**)

A total of 105 mg, 58.3% yield over two steps: ^1^H NMR (400 MHz, DMSO) δ 8.90 (t, *J* = 6.3 Hz, 1H), 8.43 (s, 1H), 7.36 (d, *J* = 2.8 Hz, 4H), 5.85–5.80 (m, 1H), 5.69 (s, 1H), 5.02 (dt, *J* = 8.2, 4.5 Hz, 1H), 4.62 (t, *J* = 5.2 Hz, 1H), 4.52 (s, 1H), 4.36 (dq, *J* = 9.3, 4.1 Hz, 1H), 3.70 (dt, *J* = 12.5, 4.2 Hz, 1H), 3.53 (dt, *J* = 12.1, 4.7 Hz, 1H), 2.21 (ddd, *J* = 14.0, 9.1, 5.5 Hz, 1H), 1.90 (ddd, *J* = 13.2, 6.2, 2.7 Hz, 1H). ^13^C NMR (101 MHz, DMSO) δ 154.80, 153.02, 149.28, 139.52, 138.26, 131.40, 129.17, 128.25, 118.49, 90.67, 81.05, 74.85, 62.25, 42.54, 33.77. HR-ESI-MS: [M + H]^+^ calcd for C_17_H_18_C_l2_N_5_O_3_, 410.0787, found 410.0807.

#### 3.1.27. (2R,3R,5S)-2-(2-Chloro-6-((2-fluorobenzyl)amino)-9H-purin-9-yl)-5-(hydroxymethyl)tetrahydrofuran-3-ol (**2c**)

A total of 83 mg, 47.8% yield over two steps: ^1^H NMR (400 MHz, DMSO) δ 8.86 (s, 1H), 8.43 (s, 1H), 7.32 (dt, *J* = 21.1, 7.1 Hz, 2H), 7.16 (dt, *J* = 15.1, 8.5 Hz, 2H), 5.82 (d, *J* = 2.0 Hz, 1H), 5.69 (d, *J* = 4.1 Hz, 1H), 5.02 (t, *J* = 5.5 Hz, 1H), 4.70 (d, *J* = 5.4 Hz, 2H), 4.52 (s, 1H), 4.36 (d, *J* = 7.7 Hz, 1H), 3.70 (d, *J* = 12.2 Hz, 1H), 3.53 (dt, *J* = 11.1, 4.7 Hz, 1H), 2.22 (ddd, *J* = 14.0, 8.6, 5.4 Hz, 1H), 1.89 (ddd, *J* = 13.1, 6.1, 2.7 Hz, 1H). ^13^C NMR (101 MHz, DMSO) δ 154.91, 152.99, 149.33, 139.55, 129.27, 128.88 (d, *J* = 8.0 Hz), 125.77 (d, *J* = 15.3 Hz), 124.30, 118.55, 115.09 (d, *J* = 21.0 Hz), 90.68, 81.06, 74.85, 62.26, 37.08, 33.78. HR-ESI-MS: [M + H]^+^ calcd for C_17_H_18_ClFN_5_O_3_, 394.1082, found 394.1092.

#### 3.1.28. (2R,3R,5S)-2-(6-((2-Bromobenzyl)amino)-2-chloro-9H-purin-9-yl)-5-(hydroxymethyl)tetrahydrofuran-3-ol (**2d**)

A total of 75 mg, 37.5% yield over two steps: ^1^H NMR (400 MHz, DMSO) δ 8.88 (t, *J* = 6.0 Hz, 1H), 8.47 (s, 1H), 7.62 (d, *J* = 7.9 Hz, 1H), 7.37–7.24 (m, 2H), 7.21 (t, *J* = 7.5 Hz, 1H), 5.85 (s, 1H), 5.70 (d, *J* = 4.2 Hz, 1H), 5.03 (t, *J* = 5.6 Hz, 1H), 4.75–4.64 (m, 1H), 4.56 (s, 1H), 4.38 (dd, *J* = 9.7, 5.3 Hz, 1H), 3.72 (dt, *J* = 12.5, 4.3 Hz, 1H), 3.55 (dt, *J* = 11.6, 4.7 Hz, 1H), 2.24 (ddd, *J* = 14.2, 9.2, 5.6 Hz, 1H), 1.96–1.86 (m, 1H). ^13^C NMR (101 MHz, DMSO) δ 154.97, 153.03, 149.38, 139.66, 137.47, 132.40, 128.87, 128.26, 127.74, 122.13, 118.60, 90.71, 81.08, 74.87, 62.28, 43.70, 33.79. HR-ESI-MS: [M + H]^+^ calcd for C_17_H_18_BrClN_5_O_3_, 454.0282, found 456.0278.

#### 3.1.29. (2R,3R,5S)-2-(2-Chloro-6-((2-iodobenzyl)amino)-9H-purin-9-yl)-5-(hydroxymethyl)tetrahydrofuran-3-ol (**2e**)

A total of 127 mg, 57.6% yield over two steps: ^1^H NMR (400 MHz, DMSO) δ 8.89 (t, *J* = 6.0 Hz, 1H), 8.47 (s, 1H), 7.91–7.84 (m, 1H), 7.34 (td, *J* = 7.5, 1.3 Hz, 1H), 7.20 (d, *J* = 7.7 Hz, 1H), 7.07–6.98 (m, 1H), 5.84 (d, *J* = 2.0 Hz, 1H), 5.70 (d, *J* = 4.1 Hz, 1H), 5.04 (t, *J* = 5.5 Hz, 1H), 4.65–4.52 (m, 2H), 4.42–4.32 (m, 1H), 3.76–3.67 (m, 1H), 3.54 (dt, *J* = 12.1, 4.8 Hz, 1H), 2.29–2.18 (m, 1H), 1.91 (ddd, *J* = 13.1, 6.2, 2.7 Hz, 1H). ^13^C NMR (101 MHz, DMSO) δ 154.91, 153.03, 149.38, 140.22, 139.66, 138.94, 128.98, 128.35, 127.56, 118.57, 98.27, 90.70, 81.06, 74.85, 62.27, 48.58, 33.80. HR-ESI-MS: [M + H]^+^ calcd for C_17_H_18_IClN_5_O_3_, 502.0143, found 502.0171.

#### 3.1.30. (2R,3R,5S)-2-(2-Chloro-6-((2-(trifluoromethyl)benzyl) amino)-9H-purin-9-yl)-5-(hydroxymethyl)tetrahydrofuran-3-ol (**2f**)

A total of 88 mg, 45.1% yield over two steps: ^1^H NMR (400 MHz, DMSO) δ 8.94 (t, *J* = 6.1 Hz, 1H), 8.48 (s, 1H), 7.75 (d, *J* = 7.8 Hz, 1H), 7.62 (t, *J* = 7.6 Hz, 1H), 7.48 (d, *J* = 7.3 Hz, 2H), 5.85 (d, *J* = 2.2 Hz, 1H), 5.70 (d, *J* = 4.1 Hz, 1H), 5.03 (t, *J* = 5.5 Hz, 1H), 4.86 (d, *J* = 4.6 Hz, 2H), 4.55 (s, 1H), 4.38 (dd, *J* = 9.4, 5.3 Hz, 1H), 3.72 (dt, *J* = 12.8, 4.2 Hz, 1H), 3.55 (dt, *J* = 11.8, 4.5 Hz, 1H), 2.24 (ddd, *J* = 14.2, 9.1, 5.6 Hz, 1H), 1.96–1.86 (m, 1H). ^13^C NMR (101 MHz, DMSO) δ 154.98, 153.00, 149.43, 139.72, 137.42, 132.70, 127.82, 127.31, 125.88, 123.16, 118.58, 90.69, 81.07, 74.85, 62.26, 33.79. HR-ESI-MS: [M + H]^+^ calcd for C_18_H_18_ClF_3_N_5_O_3_, 444.1050, found 444.1081.

#### 3.1.31. 2-(((2-Chloro-9-((2R,3R,5S)-3-hydroxy-5-(hydroxymethyl)tetrahydrofuran-2-yl)-9H-purin-6-yl)amino)methyl)benzonitr-ile (**2g**)

A total of 78 mg, 44.3% yield over two steps: ^1^H NMR (400 MHz, DMSO) δ 10.56 (s, 1H), 8.79 (s, 1H), 7.86 (d, *J* = 7.6 Hz, 1H), 7.72–7.62 (m, 2H), 7.52 (d, *J* = 7.7 Hz, 1H), 5.97 (s, 1H), 5.79 (s, 1H), 5.52 (s, 2H), 5.11 (s, 1H), 4.59 (s, 1H), 4.43 (s, 1H), 3.77 (d, *J* = 12.0 Hz, 1H), 3.58 (d, *J* = 12.3 Hz, 1H), 2.25 (s, 1H), 1.96–1.88 (m, 1H). ^13^C NMR (101 MHz, DMSO) δ 158.32, 152.71, 152.55, 151.71, 142.00, 140.31, 133.84, 132.29, 128.66, 123.77, 123.20, 120.47, 91.35, 81.97, 75.49, 62.38, 54.50, 33.94. HR-ESI-MS: [M + H]+ calcd for C_18_H_18_ClN_6_O_3_, 401.1129, found 401.1137.

#### 3.1.32. (2R,3R,5S)-2-(2-Chloro-6-((2-hydroxybenzyl)amino)-9H-purin-9-yl)-5-(hydroxy methyl)tetrahydrofuran-3-ol (**2h**)

A total of 63 mg, 36.6% yield over two steps: ^1^H NMR (400 MHz, DMSO) δ 9.60 (s, 1H), 8.60 (t, *J* = 5.9 Hz, 1H), 8.43 (s, 1H), 7.07 (t, *J* = 7.8 Hz, 2H), 6.82 (d, *J* = 7.9 Hz, 1H), 6.73 (t, *J* = 7.4 Hz, 1H), 5.83 (d, *J* = 2.1 Hz, 1H), 5.69 (d, *J* = 4.1 Hz, 1H), 5.03 (t, *J* = 5.5 Hz, 1H), 4.63–4.51 (m, 3H), 4.36 (dt, *J* = 9.4, 5.1 Hz, 1H), 3.71 (dt, *J* = 9.3, 3.7 Hz, 1H), 3.54 (dt, *J* = 12.2, 4.8 Hz, 1H), 2.23 (ddd, *J* = 14.1, 9.0, 5.6 Hz, 1H), 1.90 (ddd, *J* = 13.1, 6.1, 2.6 Hz, 1H). ^13^C NMR (101 MHz, DMSO) δ 155.11, 154.74, 153.04, 139.43, 127.75, 127.61, 124.86, 118.82, 114.97, 90.69, 81.04, 74.85, 62.28, 33.80. HR-ESI-MS: [M + H]^+^ calcd for C_17_H_19_ClN_5_O_4_, 392.1129, found 392.1152.

#### 3.1.33. (2R,3R,5S)-2-(2-Chloro-6-((2-methoxybenzyl)amino)-9H-purin-9-yl)-5-(hydroxymethyl)tetrahydrofuran-3-ol (**2i**)

A total of 91 mg, 51.1% yield over two steps: ^1^H NMR (400 MHz, DMSO) δ 8.64 (t, *J* = 6.2 Hz, 1H), 8.43 (s, 1H), 7.27–7.18 (m, 1H), 7.11 (d, *J* = 7.4 Hz, 1H), 6.99 (d, *J* = 8.2 Hz, 1H), 6.86 (t, *J* = 7.4 Hz, 1H), 5.83 (d, *J* = 2.1 Hz, 1H), 5.69 (d, *J* = 4.0 Hz, 1H), 5.03 (t, *J* = 5.6 Hz, 1H), 4.62 (dd, *J* = 6.3, 3.0 Hz, 1H), 4.54 (s, 1H), 4.37 (q, *J* = 8.8, 6.9 Hz, 1H), 3.83 (s, 3H), 3.71 (dt, *J* = 12.2, 4.3 Hz, 1H), 3.54 (dt, *J* = 12.4, 4.8 Hz, 1H), 2.23 (ddd, *J* = 14.2, 8.9, 5.5 Hz, 1H), 1.90 (ddd, *J* = 13.2, 6.2, 2.7 Hz, 1H). ^13^C NMR (101 MHz, DMSO) δ 156.57, 155.19, 153.11, 149.21, 139.42, 127.94, 126.87, 126.44, 120.12, 118.54, 110.44, 90.68, 81.03, 74.84, 62.29, 55.34, 38.46, 33.81. HR-ESI-MS: [M + H]^+^ calcd for C_18_H_21_ClN_5_O_4_, 406.1282, found 406.1300.

#### 3.1.34. (2R,3R,5S)-2-(2-Chloro-6-(((S)-1-(2-chlorophenyl)ethyl) amino)-9H-purin-9-yl)-5-(hydroxymethyl)tetrahydrofuran-3-ol (**2j**)

A total of 81 mg, 43.5% yield over two steps: ^1^H NMR (400 MHz, DMSO) δ 9.01 (d, *J* = 8.0 Hz, 1H), 8.45 (s, 1H), 7.57 (d, *J* = 7.8 Hz, 1H), 7.41 (d, *J* = 7.7 Hz, 1H), 7.33–7.20 (m, 2H), 5.81 (d, *J* = 2.0 Hz, 1H), 5.67 (d, *J* = 4.2 Hz, 1H), 5.03 (t, *J* = 5.6 Hz, 1H), 4.49 (s, 1H), 4.35 (q, *J* = 6.0 Hz, 1H), 3.75–3.66 (m, 1H), 3.53 (dd, *J* = 11.2, 6.0 Hz, 1H), 2.20 (ddd, *J* = 14.0, 9.0, 5.4 Hz, 1H), 1.88 (ddd, *J* = 13.2, 6.1, 2.5 Hz, 1H), 1.50 (d, *J* = 6.9 Hz, 3H). ^13^C NMR (101 MHz, DMSO) δ 153.90, 152.87, 149.43, 142.10, 139.41, 131.65, 129.20, 128.40, 127.42, 127.08, 118.42, 90.64, 81.03, 74.86, 62.23, 46.61, 33.71, 20.92. HR-ESI-MS: [M + H]^+^ calcd for C_18_H_20_Cl_2_N_5_O_3_, 424.0943, found 424.0965.

#### 3.1.35. (2R,3R,5S)-2-(2-Chloro-6-(((R)-1-(2-chlorophenyl)ethyl) amino)-9H-purin-9-yl)-5-(hydroxymethyl)tetrahydrofuran-3-ol (**2k**)

A total of 87 mg, 46.7% yield over two steps: ^1^H NMR (400 MHz, DMSO) δ 8.99 (d, *J* = 7.9 Hz, 1H), 8.44 (s, 1H), 7.58 (d, *J* = 7.8 Hz, 1H), 7.41 (d, *J* = 7.8 Hz, 1H), 7.27 (dt, *J* = 24.2, 7.4 Hz, 2H), 5.81 (s, 1H), 5.67 (q, *J* = 11.3, 9.2 Hz, 1H), 5.01 (t, *J* = 5.5 Hz, 1H), 4.53 (s, 1H), 4.40–4.31 (m, 1H), 3.70 (dt, *J* = 12.4, 4.6 Hz, 1H), 3.52 (dt, *J* = 12.1, 5.0 Hz, 1H), 2.22 (ddd, *J* = 14.2, 9.1, 5.5 Hz, 1H), 1.90 (ddd, *J* = 5.9 Hz, 1H), 1.50 (d, *J* = 6.9 Hz, 3H). ^13^C NMR (101 MHz, DMSO) δ 153.93, 152.87, 149.45, 142.00, 139.45, 131.67, 129.21, 128.41, 127.43, 127.09, 118.45, 90.66, 81.01, 74.79, 62.28, 54.91, 46.62, 33.81, 20.91. HR-ESI-MS: [M + H]^+^ calcd for C_18_H_20_Cl_2_N_5_O_3_, 424.0943, found 424.0967.

#### 3.1.36. (2R,3R,5S)-2-(2-Chloro-6-((1-(2-chlorophenyl)cyclopropyl)amino)-9H-purin-9-yl)-5-(hydroxymethyl)tetrahydrofuran-3-ol (**2l**)

A total of 53 mg, 27.7% yield over two steps: ^1^H NMR (400 MHz, DMSO) δ 8.84 (s, 1H), 8.40 (s, 1H), 7.91 (d, *J* = 7.6 Hz, 1H), 7.35 (d, *J* = 7.8 Hz, 1H), 7.32–7.19 (m, 2H), 5.78 (s, 1H), 5.64 (d, *J* = 4.1 Hz, 1H), 5.00 (t, *J* = 5.5 Hz, 1H), 4.48 (q, *J* = 4.7, 3.7 Hz, 1H), 4.34 (dq, *J* = 9.2, 4.1 Hz, 1H), 3.69 (dq, *J* = 12.6, 4.1, 3.4 Hz, 1H), 3.56–3.46 (m, 1H), 2.23–2.12 (m, 1H), 1.86 (ddd, *J* = 13.2, 6.0, 2.6 Hz, 1H), 1.42–1.30 (m, 2H), 1.30–1.10 (m, 2H). ^13^C NMR (101 MHz, DMSO) δ 154.92, 152.43, 149.32, 139.44, 138.57, 134.97, 133.81, 129.07, 128.87, 125.93, 118.72, 90.67, 81.04, 74.81, 62.20, 35.25, 33.66, 14.52. HR-ESI-MS: [M + H]^+^ calcd for C_19_H_20_Cl_2_N_5_O_3_, 436.0943, found 436.0963.

#### 3.1.37. General Procedure C for the Synthesis of **16a**~**16d**

A solution of 2-chlorobenzylamine (400 mg, 2.83 mg, 1 eq), N,N-diisopropyl-ethylamine (730 mg, 5.66 mmol, 2 eq), and the appropriate iodoalkane (1.5 eq) in acetonitrile (2 mL) was heated to 90 °C in a sealed tube. After stirring for 20 h, the mixture was cooled to room temperature and then concentrated under reduced pressure. The residue was partitioned between water and ethyl acetate. The organic phase was washed, dried, and concentrated. The residue was used directly in the next step without purification.

#### 3.1.38. (2R,3R,5S)-2-(2-Chloro-6-((2-chlorobenzyl)(methyl) amino)-9H-purin-9-yl)-5-(hydroxymethyl)tetrahydrofuran-3-ol (**3a**)

A total of 43 mg, 23.0% yield over two steps: ^1^H NMR (400 MHz, DMSO) δ 8.38 (s, 1H), 7.49 (dd, *J* = 7.1, 2.0 Hz, 1H), 7.33–7.25 (m, 2H), 7.13 (s, 1H), 5.86 (s, 1H), 5.71 (d, *J* = 4.1 Hz, 1H), 5.60 (s, 1H), 5.01 (s, 2H), 4.51 (s, 1H), 4.37 (s, 1H), 3.69 (s, 2H), 3.53 (s, 1H), 3.18 (s, 2H), 2.20 (dt, *J* = 13.7, 7.4 Hz, 1H), 1.89 (ddd, *J* = 13.2, 6.0, 2.6 Hz, 1H). ^13^C NMR (101 MHz, DMSO) δ 154.68, 152.53, 138.74, 129.56, 128.82, 127.55, 118.50, 90.58, 81.13, 74.90, 62.17, 54.90, 33.74. HR-ESI-MS: [M + H]^+^ calcd for C_18_H_20_Cl_2_N_5_O_3_, 424.0943, found 424.0966.

#### 3.1.39. (2R,3R,5S)-2-(2-Chloro-6-((2-chlorobenzyl)(ethyl)amino)-9H-purin-9-yl)-5-(hydroxymethyl)tetrahydrofuran-3-ol (**3b**)

A total of 51 mg, 26.5% yield over two steps: ^1^H NMR (400 MHz, DMSO) δ 8.44 (d, *J* = 59.1 Hz, 1H), 7.49 (dd, *J* = 7.4, 1.8 Hz, 1H), 7.35–7.25 (m, 2H), 7.18 (s, 1H), 5.85 (s, 1H), 5.69 (d, *J* = 4.1 Hz, 1H), 5.65–5.45 (m, 1H), 5.00–4.95 (m, 2H), 4.51 (s, 1H), 4.36 (s, 1H), 4.19 (s, 1H), 3.71–3.66 (m, 2H), 3.55–3.51 (m, 1H), 2.23–2.18 (m, 1H), 1.89 (ddd, *J* = 13.0 Hz, 1H), 1.20 (t, *J* = 8.3, 7.1 Hz, 3H). ^13^C NMR (101 MHz, DMSO) δ 153.67, 152.08, 150.45, 138.58, 138.20, 131.62, 128.88, 128.21, 127.81, 127.25, 126.86, 117.76, 89.98, 80.51, 74.27, 69.19, 61.59, 33.14, 28.42. HR-ESI-MS: [M + H]^+^ calcd for C_19_H_22_Cl_2_N_5_O_3_, 438.1100, found 438.1145.

#### 3.1.40. (2R,3R,5S)-2-(2-Chloro-6-((2-chlorobenzyl)(isopropyl) amino)-9H-purin-9-yl)-5-(hydroxymethyl)tetrahydrofuran-3-ol (**3c**)

A total of 47 mg, 23.7% yield over two steps: ^1^H NMR (400 MHz, DMSO) δ 8.40 (d, *J* = 95.4 Hz, 1H), 7.46 (dt, *J* = 7.7, 1.9 Hz, 1H), 7.29–7.15 (m, 2H), 7.13–7.06 (m, 1H), 6.15 (s, 1H), 5.85 (s, 1H), 5.69 (d, *J* = 4.4 Hz, 1H), 5.47–5.09 (m, 1H), 4.99 (d, *J* = 15.4 Hz, 1H), 4.84 (s, 1H), 4.51 (s, 1H), 4.36 (s, 1H), 3.75–3.60 (m, 1H), 3.57–3.47 (m, 1H), 2.20 (t, *J* = 13.3 Hz, 1H), 1.93–1.86 (m, 2H), 1.19 (d, *J* = 7.4 Hz, 6H). ^13^C NMR (101 MHz, DMSO) δ 152.42, 151.16, 138.76, 131.12, 129.14, 128.18, 127.66, 127.43, 127.08, 90.53, 81.08, 74.83, 62.18, 33.74, 20.28, 19.36. HR-ESI-MS: [M + H]^+^ calcd for C_20_H_24_Cl_2_N_5_O_3_, 452.1256, found 452.1301.

#### 3.1.41. (2R,3R,5S)-2-(2-Chloro-6-((2-chlorobenzyl)(2-fluoroethyl)amino)-9H-purin-9-yl)-5-(hydroxymethyl)tetrahydrofuran-3-ol (**3d**)

A total of 84 mg, 35.8% yield over two steps: ^1^H NMR (400 MHz, DMSO) δ 8.45 (s, 1H), 7.56–7.39 (m, 2H), 7.34–7.25 (m, 6H), 5.86 (d, *J* = 1.9 Hz, 1H), 5.70 (d, *J* = 4.2 Hz, 1H), 5.69–5.48 (m, 2H), 5.06–4.89 (m, 3H), 4.53 (tt, *J* = 4.9, 2.3 Hz, 1H), 4.36 (ddt, *J* = 9.6, 6.5, 3.7 Hz, 1H), 3.69 (ddd, *J* = 12.0, 5.6, 3.4 Hz, 1H), 3.52 (ddd, *J* = 12.1, 5.5, 4.0 Hz, 1H), 2.20 (ddd, *J* = 13.2, 9.2, 5.5 Hz, 1H), 1.90 (ddd, *J* = 13.1, 6.1, 2.6 Hz, 1H). ^13^C NMR (101 MHz, DMSO) δ 155.24, 152.92, 151.86, 139.87, 135.03, 134.23, 132.88, 129.97, 129.40, 128.80, 128.66, 128.01, 118.97, 91.08, 81.63, 75.32, 62.65, 50.43, 48.66, 34.23. HR-ESI-MS: [M + H]^+^ calcd for C_24_H_23_Cl_3_N_5_O_3_, 534.0866, found 534.0878.

### 3.2. Biological Assays

#### 3.2.1. Cell Culture

The human hepatic stellate cell line LX-2 was obtained from Procell Life Science & Technology Co., Ltd. (Wuhan, China), and primary mouse hepatocytes (MPH) were isolated from C57BL/6J mice. LX-2 cells were maintained in high-glucose DMEM (DMEM-HG) supplemented with 2% fetal bovine serum (FBS) and 1% penicillin–streptomycin. MPH cells were cultured in low-glucose DMEM (DMEM-LG) containing 10% FBS and 1% penicillin-streptomycin. All cells were incubated at 37 °C in a humidified atmosphere of 5% CO_2_ and were passaged upon reaching 80% confluence.

#### 3.2.2. Cell Treatment

Digest confluent LX-2 cells using 0.25% trypsin-EDTA, followed by centrifugation at 300× *g* for 3 min. Resuspend the cell pellet in complete medium (DMEM-HG supplemented with 2% FBS) and perform cell counting using a hemocytometer or automated cell counter. Seed the cell suspension into 12-well culture plates at a density of 2 × 10^4^ cells/mL (1 mL/well) in DMEM-HG containing 2% FBS. After 12 h of incubation to ensure cell adhesion, replace the medium with serum-reduced starvation medium (DMEM-HG containing 0.5% FBS) for an additional 12 h to synchronize cell cycle progression. Prepare a 10 ng/mL solution of TGF-β in starvation medium. Dilute test compounds to the desired concentrations in the same medium. Add the compound solutions to respective wells, ensuring the following controls are included: DMSO solvent control (Vehicle, with TGF-β); untreated control (Control, without TGF-β). Maintain cells at 37 °C in a 5% CO_2_ humidified incubator for 24 h before harvesting for downstream analyses.

#### 3.2.3. Quantitative Real-Time Polymerase Chain Reaction

Extract total RNA from treated LX-2 cells using TRIzol reagent (Invitrogen) following the manufacturer’s protocol. Reverse transcribe 1 µg of total RNA into cDNA using ABScript III RT Master Mix (Abclonal), including a gDNA removal step. Perform qPCR amplification using 2X Universal SYBR Green Fast qPCR Mix (Abclonal). The transcript level was normalized with GAPDH mRNA in the same samples. Primer sequences are provided in Supplementary Materials Table S1. The α-SMA inhibition rate was subsequently calculated using the formula:

Inhibition rate (%) = [1 − (Relative expression of treated group/Relative expression of control)] × 100%.

#### 3.2.4. Western Blot Analysis

Aspirate the medium and lyse cells on ice using RIPA buffer supplemented with 1× protease and phosphatase inhibitors for 30 min. Collect lysates, sonicate briefly to ensure complete lysis, and mix with 4× Laemmli loading buffer. Denature samples at 95 °C for 10 min. Separate proteins via SDS-PAGE, transfer to PVDF membranes, and probe with primary antibodies against target proteins. Detect protein bands using chemiluminescent substrate and visualize with an imaging system.

#### 3.2.5. CTG Assay

Plate LX-2 cells or MPH cells in 96-well white plates at 5 × 10^4^ cells/mL or 20 × 10^4^ cells/mL (100 µL/well) and incubate overnight for adherence. Dilute test compounds to the desired concentrations, and add the compound solutions to respective wells. After treatment, equilibrate plates to room temperature, add 100 µL of CellTiter-Glo^®^ reagent (Promega) per well, and agitate for 2 min to induce cell lysis. Allow the reaction to stabilize for 10 min at room temperature, then measure luminescence using a microplate reader. Cell viability for a single compound concentration is expressed as a percentage relative to the vehicle-treated control (set at 100% viability), calculated as follows:

Viability (%) = (Mean Luminescence of Test Well/Mean Luminescence of Control Wells) × 100.

The CC_50_ values were determined from full dose–response curves. The dose–response data were then fitted using a four-parameter logistic nonlinear regression model in GraphPad Prism software (version 8.0), from which the CC_50_ value and its 95% confidence interval were derived.

#### 3.2.6. Cell Scratch Test

LX-2 cells were seeded in a 96-well plate with 8 × 10^5^ cells/mL and starved with serum-free medium for 24 h. Linear wounds were created in the monolayers by scratching with a sterilized pipette tip, and then the floating cells were gently washed with PBS. Conditioned medium (DMEM with 10% FBS + DMSO, and DMEM with 10% FBS+ different concentrations of **3a** and cordycepin) was added, and the scratch width was measured and recorded at 0 and 24 h. The wound healing rate was calculated as the percentage of closure using the formula:

Wound healing rate% = [(Area_0h_ − Area_24h_)/Area_0h_] × 100.

#### 3.2.7. Microsome Stability Measurements

The compound (1 µM) was incubated with liver microsomes (0.5 mg/mL) in the presence of an NADPH-regenerating system at 37 °C. Aliquots were taken at predetermined time points over 60 min, and the reactions were quenched with cold acetonitrile. The concentration of the parent compound remaining at each time point was quantified using liquid chromatography–tandem mass spectrometry (LC-MS/MS). The in vitro half-life (T_1_/_2_) and intrinsic clearance (CLint) were then calculated from the natural logarithm of the remaining compound percentage versus time plot, allowing for a direct comparison of metabolic clearance rates between the two species.

#### 3.2.8. Statistic

All results are presented as the mean ± SEM. A *p*-value of less than 0.05 was considered statistically significant. All statistical analyses were performed using GraphPad Prism 8.0.

## 4. Conclusions

In summary, we designed and synthesized a novel series of twenty-eight cordycepin derivatives. The SAR results indicated that the introduction of suitable substituents on the C6-NH_2_ and C2 positions might be beneficial for activity. Among these, compound **3a** emerged as a promising analog, maintaining the anti-fibrotic activity of cordycepin while demonstrating slightly improved metabolic stability in liver microsomes and, notably, no observable toxicity in primary hepatocytes at 100 µM. Furthermore, **3a** was found to increase the phosphorylation of AMPK and its downstream target ACC, pointing to a plausible interaction with this pathway and establishing a basis for further causal validation. Future efforts will focus on optimizing **3a**’s metabolic stability, investigating its potential mechanism, and evaluating the in vivo efficacy to develop more potent anti-fibrotic agents with favorable drug-like properties.

## Figures and Tables

**Table 1:** The inhibition rates of compounds **1a**~**1l** on activated LX-2 cells.

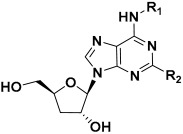				
Compounds			α-SMA Inhibition (%) ^a^	
No	R_1_	R_2_	10 μM	5 μM
**1a**	-CH_3_	H	6.0 ± 3.4	<5
**1b**	-CH(CH_3_)_2_	H	<5	<5
**1c**	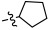	H	<5	<5
**1d**	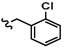	H	13.9 ± 2.7	<5
**1e**	H	Cl	8.4 ± 1.8	<5
**1f**	-CH_3_	Cl	8.7 ± 2.7	16.6 ± 3.4
**1g**	-CH(CH_3_)_2_	Cl	23.3 ± 3.0	<5
**1h**	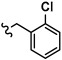	Cl	62.3 ± 0.6	35.7 ± 5.3
**1i**	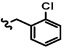	F	51.4 ± 1.1	31.7 ± 1.6
**1j**	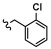	I	56.6 ± 3.5	46.9 ± 2.0
**1k**	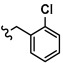	OH	8.2 ± 3.7	19.8 ± 1.0
**1l**	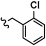	NH_2_	23.6 ± 0.9	17.7 ± 1.8
**Cordycepin**			52.9 ± 1.0	42.3 ± 2.4

^a^ Means the results were expressed as the mean value of three experiments.

**Table 2:** The inhibition rates of compounds **2a**~**2l** on activated LX-2 cells.

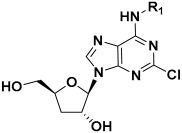			
Compounds		α-SMA Inhibition (%) ^a^	
No	R_1_	10 μM	5 μM
**2a**	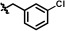	49.6 ± 1.0	51.9 ± 1.0
**2b**	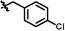	57.7 ± 1.2	52.6 ± 1.2
**2c**	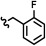	25.8 ± 5.7	18.4 ± 3.7
**2d**	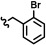	52.7 ± 0.3	55.7 ± 3.3
**2e**	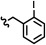	40.2 ± 1.9	46.5 ± 3.9
**2f**	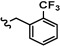	55.9 ± 4.8	46.8 ± 2.2
**2g**	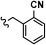	51.0 ± 2.0	24.4 ± 5.6
**2h**	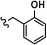	41.6 ± 3.3	22.5 ± 5.4
**2i**	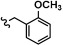	54.2 ± 2.8	47.7 ± 3.2
**2j**	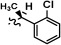	57.8 ± 0.8	51.9 ± 1.0
**2k**	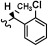	59.4 ± 1.2	48.6 ± 4.2
**2l**	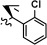	50.8 ± 1.9	46.2 ± 4.2
**1h**		62.3 ± 0.6	35.7 ± 5.3

^a^ Means the results were expressed as the mean value of three experiments.

**Table 3:** The inhibition rates of compounds **3a**~**3d** on activated LX-2 cells.

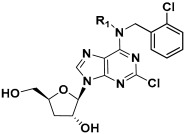			
Compounds		α-SMA Inhibition (%) ^a^	
No	R_1_	10 μM	5 μM
**3a**	-CH_3_	61.6 ± 0.7	58.3 ± 4.8
**3b**	-CH_2_CH_3_	64.4 ± 1.2	57.9 ± 1.2
**3c**	-CH(CH_3_)_2_	60.0 ± 1.7	58.8 ± 1.9
**3d**	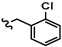	69.7 ± 2.3	54.0 ± 3.4
**1h**	H	62.3 ± 0.6	35.7 ± 5.3

^a^ Means the results were expressed as the mean value of three experiments.

**Table 4:** α-SMA inhibition activity and cytotoxicity ^a^ of the selected compounds.

Compounds	α-SMA IC_50_ (μM) in LX-2 Cell	CC_50_ (μM) in LX-2 Cell	Cell Viability @100 μM in MPH ^b^
Cordycepin	8.77 ± 2.3	50.96 ± 7.2	55.2 ± 1.4%
**3a**	7.69 ± 5.1	33.41 ± 3.7	107.5 ± 2.6%
**3b**	N.D ^c^	15.70 ± 2.4	111.0 ± 3.6%

^a^ The cytotoxicity experiments were assayed by CellTiter-Glo. ^b^ MPH means primary mouse hepatocytes. ^c^ N.D means Not Determined.

**Table 5:** Metabolic stability of cordycepin, **3a**, and **3b**.

Compounds	Liver Microsomes	T_1/2_ (min)	CL_int (liver)_ (mL/min/kg)
**3a**	human	35.57 ± 1.2	48.87 ± 1.6
	mouse	26.50 ± 2.8	205.92 ± 21.2
**3b**	human	18.32 ± 0.8	94.87 ± 4.3
	mouse	19.79 ± 2.3	275.71 ± 31.2
Cordycepin	human	27.21 ± 2.9	63.88 ± 6.6
	mouse	15.54 ± 0.2	351.26 ± 4.6

## Data Availability

The data supporting this article are contained within the article and Supplementary Materials. Further inquiries can be directed to the corresponding authors.

## Supplementary Materials

The following supporting information can be downloaded at: https://www.mdpi.com/article/10.3390/molecules31020264/s1. Table S1: qPCR Primer Sequence; Figure S1: Western blot gel raw images; Figures S2–S37: NMR spectrums and HR-MS.

## References

[1] Kisseleva T., Brenner D. (2021). Molecular and cellular mechanisms of liver fibrosis and its regression. Nat. Rev. Gastroenterol. Hepatol..

[2] Henderson N.C., Rieder F., Wynn T.A. (2020). Fibrosis: From mechanisms to medicines. Nature.

[3] Rockey D.C., Bell P.D., Hill J.A. (2015). Fibrosis—A common pathway to organ injury and failure. N. Engl. J. Med..

[4] Horn P., Tacke F. (2024). Metabolic reprogramming in liver fibrosis. Cell Metab..

[5] Ginès P., Krag A., Abraldes J.G., Solà E., Fabrellas N., Kamath P.S. (2021). Liver cirrhosis. Lancet.

[6] Nozaki A., Chuma M., Hara K., Moriya S., Fukuda H., Numata K., Tanaka K., Morimoto M., Sakamaki K., Yamanaka T. (2021). Sofosbuvir-based therapies associated with regression of liver fibrosis in patients with hepatitis C virus infection: A prospective observational study. Medicine.

[7] Wang X., Wang L., Geng L., Tanaka N., Ye B. (2023). Resmetirom Ameliorates NASH-Model Mice by Suppressing STAT3 and NF-κB Signaling Pathways in an RGS5-Dependent Manner. Int. J. Mol. Sci..

[8] Higashi T., Friedman S.L., Hoshida Y. (2017). Hepatic stellate cells as key target in liver fibrosis. Adv. Drug Deliv. Rev..

[9] Tsuchida T., Friedman S.L. (2017). Mechanisms of hepatic stellate cell activation. Nat. Rev. Gastroenterol. Hepatol..

[10] Puche J.E., Saiman Y., Friedman S.L. (2013). Hepatic stellate cells and liver fibrosis. Compr. Physiol..

[11] Baghaei K., Mazhari S., Tokhanbigli S., Parsamanesh G., Alavifard H., Schaafsma D., Ghavami S. (2022). Therapeutic potential of targeting regulatory mechanisms of hepatic stellate cell activation in liver fibrosis. Drug Discov. Today.

[12] Tuli H.S., Sharma A.K., Sandhu S.S., Kashyap D. (2013). Cordycepin: A bioactive metabolite with therapeutic potential. Life Sci..

[13] Yang D., Peng N., Zhang H., Qiu Z., Xu L., Pan M. (2025). Cordycepin ameliorates autoimmunity by promoting STING degradation via autophagy pathway. Br. J. Pharmacol..

[14] Imamura K., Asai M., Sugamoto K., Matsumoto T., Yamasaki Y., Kamei I., Hattori T., Kishimoto M., Niisaka S., Kubo M. (2015). Suppressing effect of cordycepin on the lipopolysaccharide-induced nitric oxide production in RAW 264.7 cells. Biosci. Biotechnol. Biochem..

[15] Han N.-R., Moon P.-D., Kim H.-M., Jeong H.-J. (2018). Cordycepin ameliorates skin inflammation in a DNFB-challenged murine model of atopic dermatitis. Immunopharmacol. Immunotoxicol..

[16] Wang Z., Wu X., Liang Y.-N., Wang L., Song Z.-X., Liu J.-L., Tang Z.-S. (2016). Cordycepin Induces Apoptosis and Inhibits Proliferation of Human Lung Cancer Cell Line H1975 via Inhibiting the Phosphorylation of EGFR. Molecules.

[17] Cui L., Zhao L., Shen G., Yu D., Yuan T., Zhang Y., Yang B. (2024). Antitumor Mechanism and Therapeutic Potential of Cordycepin Derivatives. Molecules.

[18] Lee H.K., Na Y.-J., Seong S.-M., Ahn D., Choi K.-C. (2024). Cordycepin Enhanced Therapeutic Potential of Gemcitabine against Cholangiocarcinoma via Downregulating Cancer Stem-Like Properties. Biomol. Ther..

[19] Lee Y.-P., Yu C.-K., Wong T.-W., Chen L.-C., Huang B.-M. (2024). Cordycepin Inhibits Enterovirus A71 Replication and Protects Host Cell from Virus-Induced Cytotoxicity through Adenosine Action Pathway. Viruses.

[20] Rottenberg M.E., Masocha W., Ferella M., Petitto-Assis F., Goto H., Kristensson K., McCaffrey R., Wigzell H. (2005). Treatment of African trypanosomiasis with cordycepin and adenosine deaminase inhibitors in a mouse model. J. Infect. Dis..

[21] Wei H.-P., Ye X.-L., Chen Z., Zhong Y.-J., Li P.-M., Pu S.-C., Li X.-G. (2009). Synthesis and pharmacokinetic evaluation of novel N-acyl-cordycepin derivatives with a normal alkyl chain. Eur. J. Med. Chem..

[22] Serpi M., Ferrari V., McGuigan C., Ghazaly E., Pepper C. (2022). Synthesis and Characterization of NUC-7738, an Aryloxy Phosphoramidate of 3′-Deoxyadenosine, as a Potential Anticancer Agent. J. Med. Chem..

[23] Wang M., Han Z., Fan B., Qu K., Zhang W., Li W., Li J., Li L., Li J., Li H. (2024). Discovery of Oral AMP-Activated Protein Kinase Activators for Treating Hyperlipidemia. J. Med. Chem..

[24] Hulpia F., Campagnaro G.D., Alzahrani K.J., Alfayez I.A., Ungogo M.A., Mabille D., Maes L., Koning H.P.d., Caljon G., van Calenbergh S. (2020). Structure-Activity Relationship Exploration of 3′-Deoxy-7-deazapurine Nucleoside Analogues as Anti-Trypanosoma brucei Agents. ACS Infect. Dis..

[25] Qu S., Wang Q., Wang Y., Li L., Zhu L., Kuang X., Wang X., Li H., Zhao L., Dai H. (2023). Design, synthesis, antibacterial/antitumor activity and in vitro stability of novel cordycepin derivatives with unsaturated fatty acid chain. Eur. J. Pharm. Sci..

[26] Liu Y., Sheng M., Jia L., Zhu M., Yu W. (2023). Protective effects of cordycepin pretreatment against liver ischemia/reperfusion injury in mice. Immun. Inflamm. Dis..

[27] Lan T., Yu Y., Zhang J., Li H., Weng Q., Jiang S., Tian S., Xu T., Hu S., Yang G. (2021). Cordycepin Ameliorates Nonalcoholic Steatohepatitis by Activation of the AMP-Activated Protein Kinase Signaling Pathway. Hepatology.

[28] Chen S., Suo J., Wang Y., Tang C., Ma B., Li J., Hou Y., Yan B., Shen T., Zhang Q. (2024). Cordycepin alleviates diabetes mellitus-associated hepatic fibrosis by inhibiting SOX9-mediated Wnt/β-catenin signal axis. Bioorg. Chem..

[29] Liang Z., Zhang K., Guo H., Tang X., Chen M., Shi J., Yang J. (2024). Cordycepin alleviates hepatic fibrosis in association with the inhibition of glutaminolysis to promote hepatic stellate cell senescence. Int. Immunopharmacol..

[30] Xu L., Hui A.Y., Albanis E., Arthur M.J., O’Byrne S.M., Blaner W.S., Mukherjee P., Friedman S.L., Eng F.J. (2005). Human hepatic stellate cell lines, LX-1 and LX-2: New tools for analysis of hepatic fibrosis. Gut.

[31] Mort C.J.W., Migaud M.E., Galione A., Potter B.V.L. (2004). Aplysia californica mediated cyclisation of novel 3′-modified NAD+ analogues: A role for hydrogen bonding in the recognition of cyclic adenosine 5′-diphosphate ribose. Bioorg. Med. Chem..

[32] Da Paixão Soares F., Silva M.J.E., Doboszewski B. (2013). Deoxygenation at the C3 position of D- and L-arabinofuranose: Stereospecific access to enantiomeric cordycepose derivatives. Carbohydr. Res..

[33] Ramasamy K.S., Tam R.C., Bard J., Averett D.R. (2000). Monocyclic L-nucleosides with type 1 cytokine-inducing activity. J. Med. Chem..

[34] Vorbrüggen H., Höfle G. (1981). Nucleoside Syntheses, XXIII 1) On the Mechanism of Nucleoside Synthesis. Chem. Ber..

[35] Piecyk K., Davis R.E., Jankowska-Anyszka M. (2012). Synthesis of N^2^-modified 7-methylguanosine 5′-monophosphates as nematode translation inhibitors. Bioorg. Med. Chem..

[36] Taniguchi Y., Sagara I., Nagata Y., Kikukawa Y., Sasaki S. (2019). Effects of the 2-substituted adenosine-1,3-diazaphenoxazine 5′-triphosphate derivatives on the single nucleotide primer extension reaction by DNA polymerase. Chem. Pharm. Bull..

[37] Xu F., Liu C., Zhou D., Zhang L. (2016). TGF-β/SMAD Pathway and Its Regulation in Hepatic Fibrosis. J. Histochem. Cytochem..

[38] Lee J.B., Radhi M., Cipolla E., Gandhi R.D., Sarmad S., Zgair A., Kim T.H., Feng W., Qin C., Adrower C. (2019). A novel nucleoside rescue metabolic pathway may be responsible for therapeutic effect of orally administered cordycepin. Sci. Rep..

[39] Tsai Y.-J., Lin L.-C., Tsai T.-H. (2010). Pharmacokinetics of adenosine and cordycepin, a bioactive constituent of Cordyceps sinensis in rat. J. Agric. Food Chem..

[40] Murakami E., Bao H., Mosley R.T., Du J., Sofia M.J., Furman P.A. (2011). Adenosine deaminase-like protein 1 (ADAL1): Characterization and substrate specificity in the hydrolysis of N(6)- or O(6)-substituted purine or 2-aminopurine nucleoside monophosphates. J. Med. Chem..

[41] Bzowska A., Kulikowska E., Shugar D. (2000). Purine nucleoside phosphorylases: Properties, functions, and clinical aspects. Pharmacol. Ther..

[42] Garcia D., Hellberg K., Chaix A., Wallace M., Herzig S., Badur M.G., Lin T., Shokhirev M.N., Pinto A.F.M., Ross D.S. (2019). Genetic Liver-Specific AMPK Activation Protects against Diet-Induced Obesity and NAFLD. Cell Rep..

[43] Zhang J., Muise E.S., Han S., Kutchukian P.S., Costet P., Zhu Y., Kan Y., Zhou H., Shah V., Huang Y. (2020). Molecular Profiling Reveals a Common Metabolic Signature of Tissue Fibrosis. Cell Rep. Med..

