# Supplementary material for: Design, Synthesis, and Anti-Hepatic Fibrosis Evaluation of Cordycepin Derivatives
Source: Molecules. 2026 Jan 12;31(2):264. doi: 10.3390/molecules31020264 (PMC12844307; doi:10.3390/molecules31020264)
Supplement: Supplementary file 1 [file molecules-31-00264-s001.zip › molecules-4063178-supplementary.pdf]

Supplementary Materials

Design, synthesis and anti-hepatic fibrosis evaluation of cordycepin derivatives

Wenfang Pan[1,2,†], Siqi Liu[1,3,4,†], Yuanchen Zhong[1,2], Bixi Tang[1], Yi Zang[1,*], Yuanchao Xie[1,*]

[1] Lingang Laboratory, Shanghai 200031, China
[2] School of Physical Science and Technology, Shanghai Tech University, Shanghai 201210, China
[3] School of Chinese Materia Medica, Nanjing University of Chinese Medicine, Nanjing, 210023, China.
[4] Stake Key Laboratory of Chemical Biology, Shanghai Institute of Materia Medica, Chinese Academy of Sciences, Shanghai, 201203, China
* Correspondence: yzang@lglab.ac.cn(Y.Z); xieyuanchao@lglab.ac.cn(Y.X)
† These authors contributed equally to this work.

Table of Contents

Table S1 qPCR Primer Sequence 4

Figure S1. Western blot gel raw images 4

^{1}H NMR、^{13}C NMR and Mass spectra of the representative compounds 5

Figure S2. ^{1}H NMR spectrum of compound 6 5

Figure S3. ^{1}H NMR spectrum of compound 8-1 5

Figure S4. ^{1}H NMR spectrum of compound 8-2 6

Figure S5. ^{1}H NMR and ^{13}C NMR spectrum of compound 1a 7

Figure S6. ^{1}H NMR，^{13}C NMR and Mass spectra spectrum of compound 1b 8

Figure S7. ^{1}H NMR，^{13}C NMR and Mass spectra spectrum of compound 1c 10

Figure S8. ^{1}H NMR，^{13}C NMR and Mass spectra spectrum of compound 1d 11

Figure S9. ^{1}H NMR，^{13}C NMR and Mass spectra spectrum of compound 1e 13

Figure S10. ^{1}H NMR，^{13}C NMR and Mass spectra spectrum of compound 1f 14

Figure S11. ^{1}H NMR，^{13}C NMR and Mass spectra spectrum of compound 1g 16

Figure S12. ^{1}H NMR，^{13}C NMR and Mass spectra spectrum of compound 1h 17

Figure S13. ^{1}H NMR spectrum of compound 10 17

Figure S14. ^{1}H NMR spectrum of compound 11 18

Figure S15. ^{1}H NMR spectrum of compound 12a 18

Figure S16. ^{1}H NMR spectrum of compound 12b 19

Figure S17. ^{1}H NMR spectrum of compound 12c 19

Figure S18. ^{1}H NMR，^{13}C NMR and Mass spectra spectrum of compound 1i 21

Figure S19. ^{1}H NMR，^{13}C NMR and Mass spectra spectrum of compound 1j 22

Figure S20. ^{1}H NMR，^{13}C NMR and Mass spectra spectrum of compound 1k 23

Figure S21. ^{1}H NMR，^{13}C NMR and Mass spectra spectrum of compound 1l 25

Figure S22. ^{1}H NMR，^{13}C NMR and Mass spectra spectrum of compound 2a 26

Figure S23. ^{1}H NMR，^{13}C NMR and Mass spectra spectrum of compound 2b 28

Figure S24. ^{1}H NMR，^{13}C NMR and Mass spectra spectrum of compound 2c 29

Figure S25. ^{1}H NMR，^{13}C NMR and Mass spectra spectrum of compound 2d 31

Figure S26. ^{1}H NMR，^{13}C NMR and Mass spectra spectrum of compound 2e 32

Figure S27. ^{1}H **NMR，** ^{13}C **NMR and Mass spectra spectrum of compound 2f........... 34**
Figure S28. ^{1}H **NMR，** ^{13}C **NMR and Mass spectra spectrum of compound 2g.......... 35**
Figure S29. ^{1}H **NMR，** ^{13}C **NMR and Mass spectra spectrum of compound 2h.......... 37**
Figure S30. ^{1}H **NMR，** ^{13}C **NMR and Mass spectra spectrum of compound 2i........... 38**
Figure S31. ^{1}H **NMR，** ^{13}C **NMR and Mass spectra spectrum of compound 2j........... 40**
Figure S32. ^{1}H **NMR，** ^{13}C **NMR and Mass spectra spectrum of compound 2k.......... 41**
Figure S33. ^{1}H **NMR，** ^{13}C **NMR and Mass spectra spectrum of compound 2l........... 43**
Figure S34. ^{1}H **NMR，** ^{13}C **NMR and Mass spectra spectrum of compound 3a.......... 44**
Figure S35. ^{1}H **NMR，** ^{13}C **NMR and Mass spectra spectrum of compound 3b.......... 46**
Figure S36. ^{1}H **NMR，** ^{13}C **NMR and Mass spectra spectrum of compound 3c.......... 47**
Figure S37. ^{1}H **NMR，** ^{13}C **NMR and Mass spectra spectrum of compound 3d.......... 49**

Table S1 qPCR Primer Sequence

Primer	Sequence
h-GAPDH-F	GTCTCCTCTGACTTCAACAGCG
h-GAPDH-R	ACCACCCTGTTGCTGTAGCCAA
h-ACTA2-F	CTATGCCTCTGGACGCACAACT
h-ACTA2-R	CAGATCCAGACGCATGATGGCA

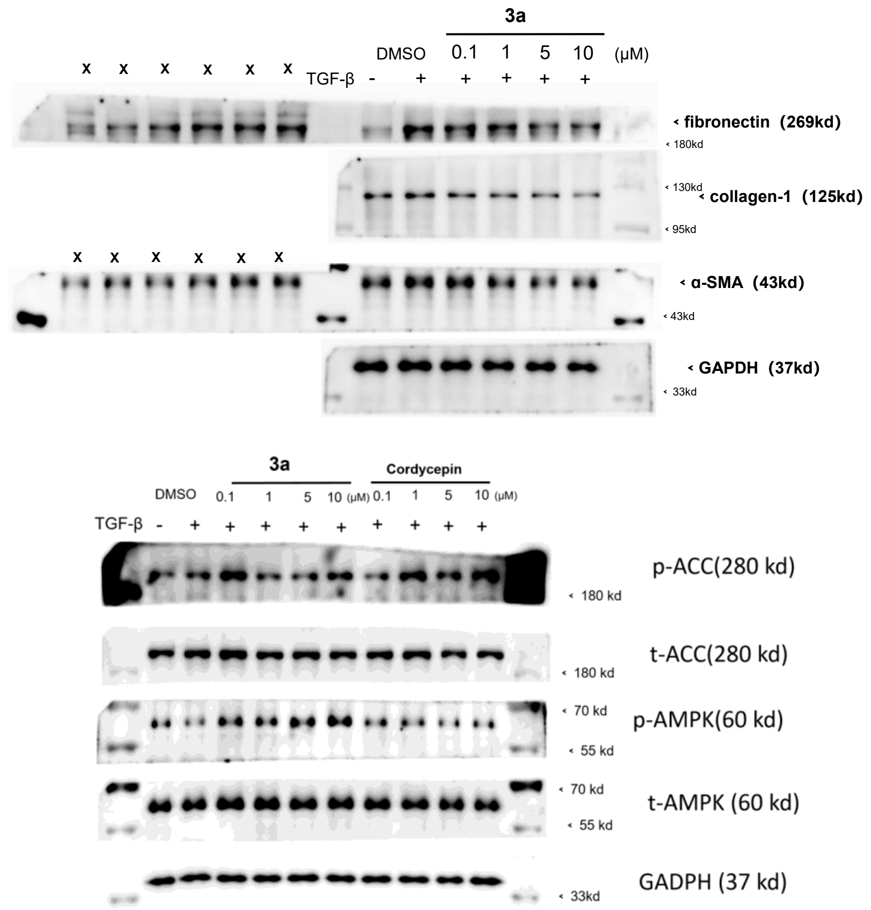

Figure S1. Western blot gel raw images

^{1}H NMR、^{13}C NMR and Mass spectra of the representative compounds

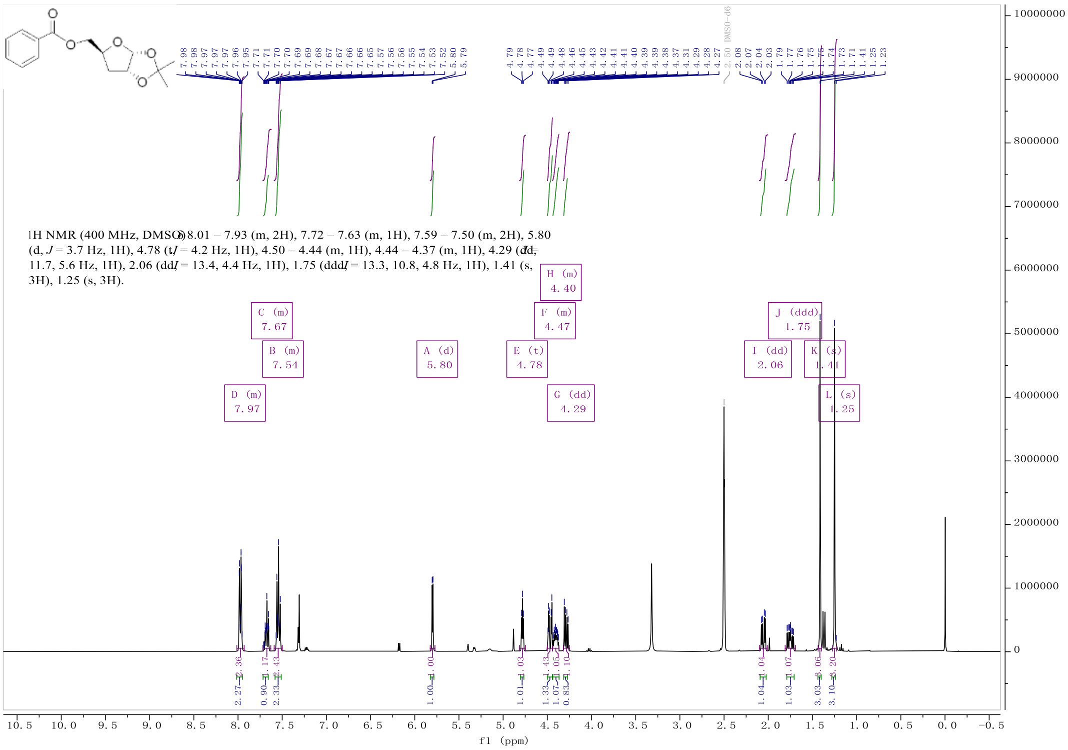

Figure S2. ^{1}**H NMR** spectrum of compound **7**

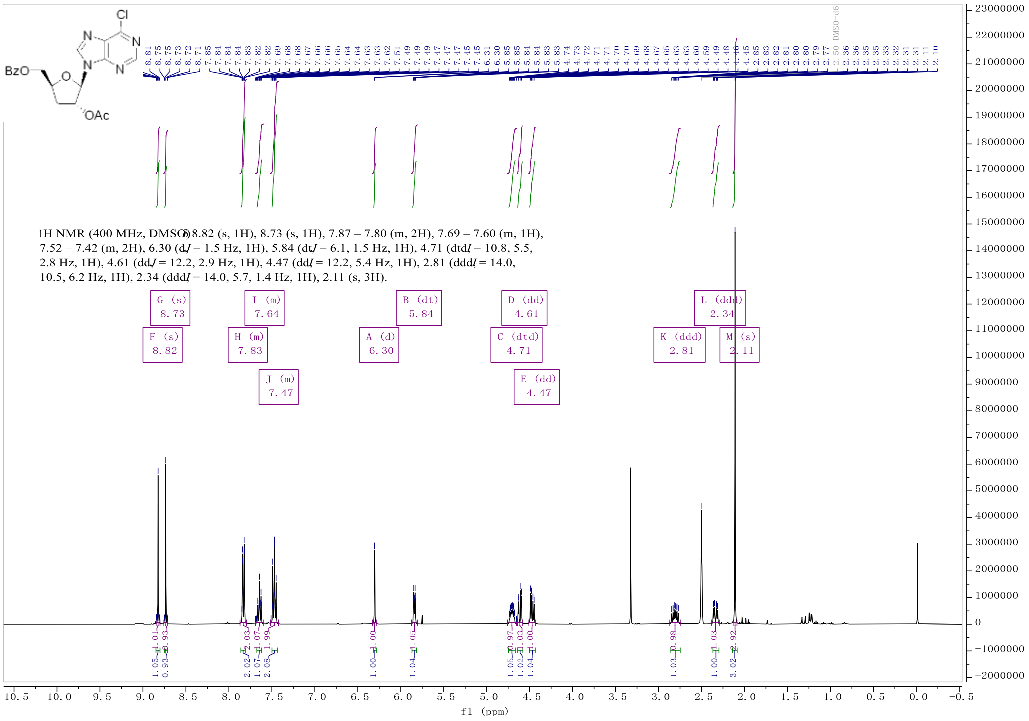

Figure S3. ^{1}**H NMR** spectrum of compound **9**

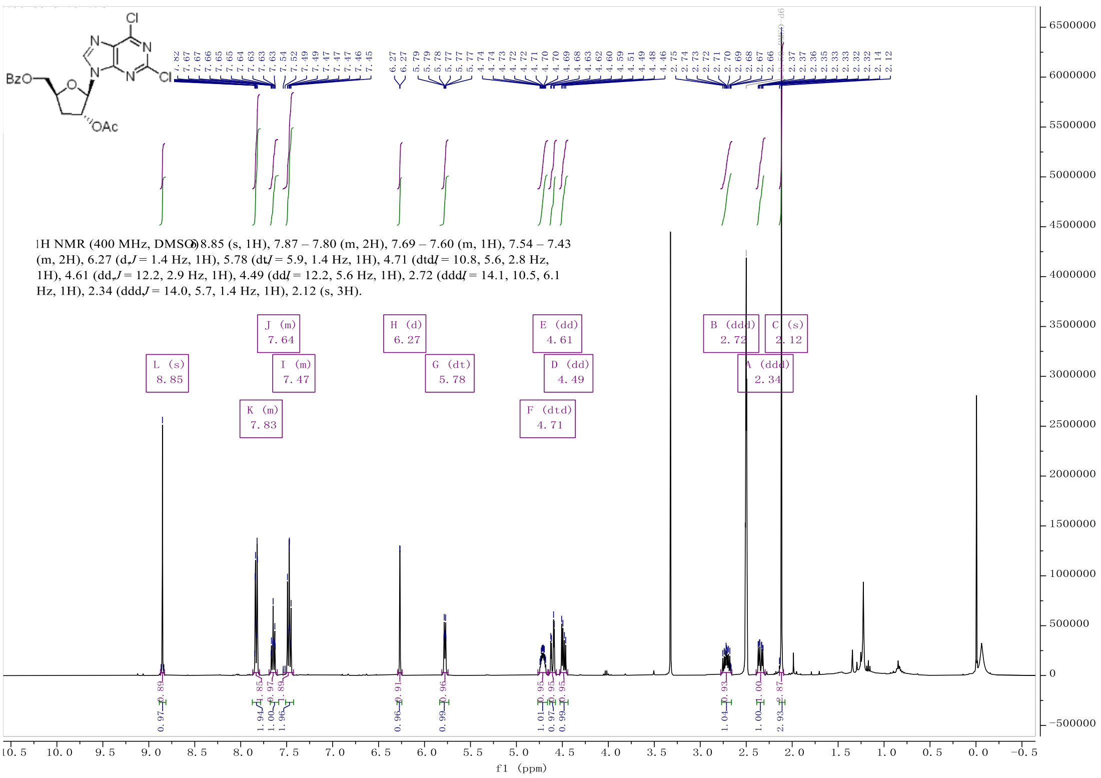

Figure S4. **^{1}H NMR** spectrum of compound **10**

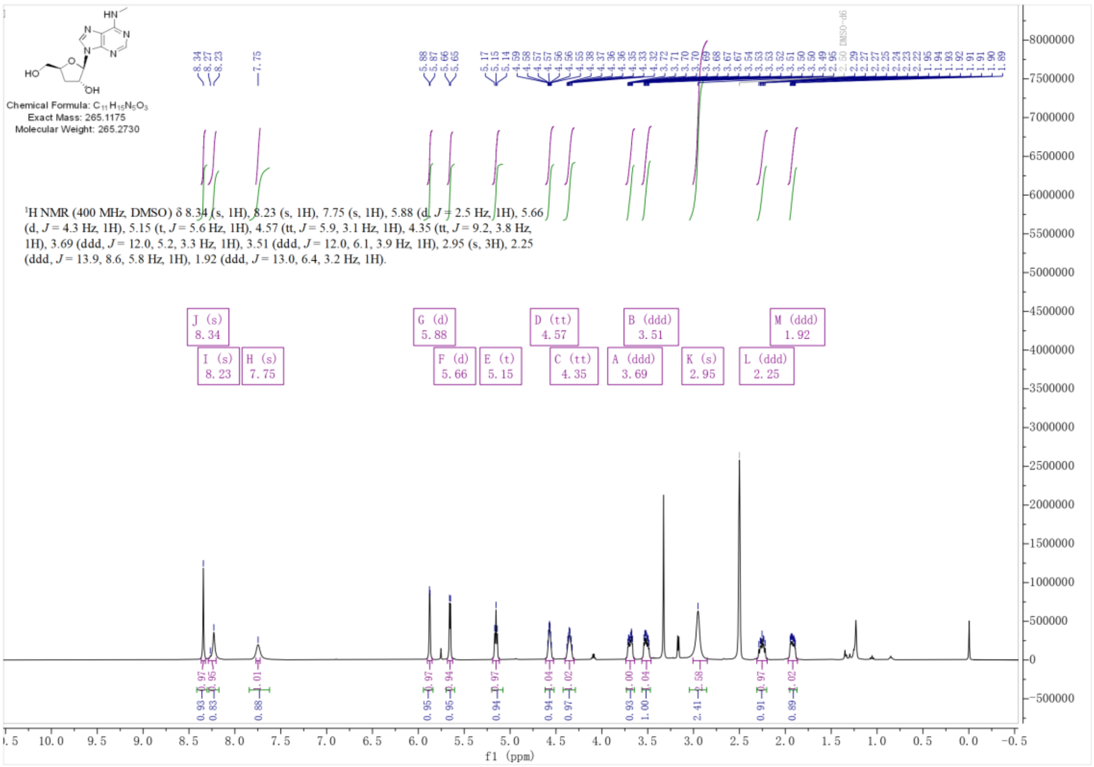

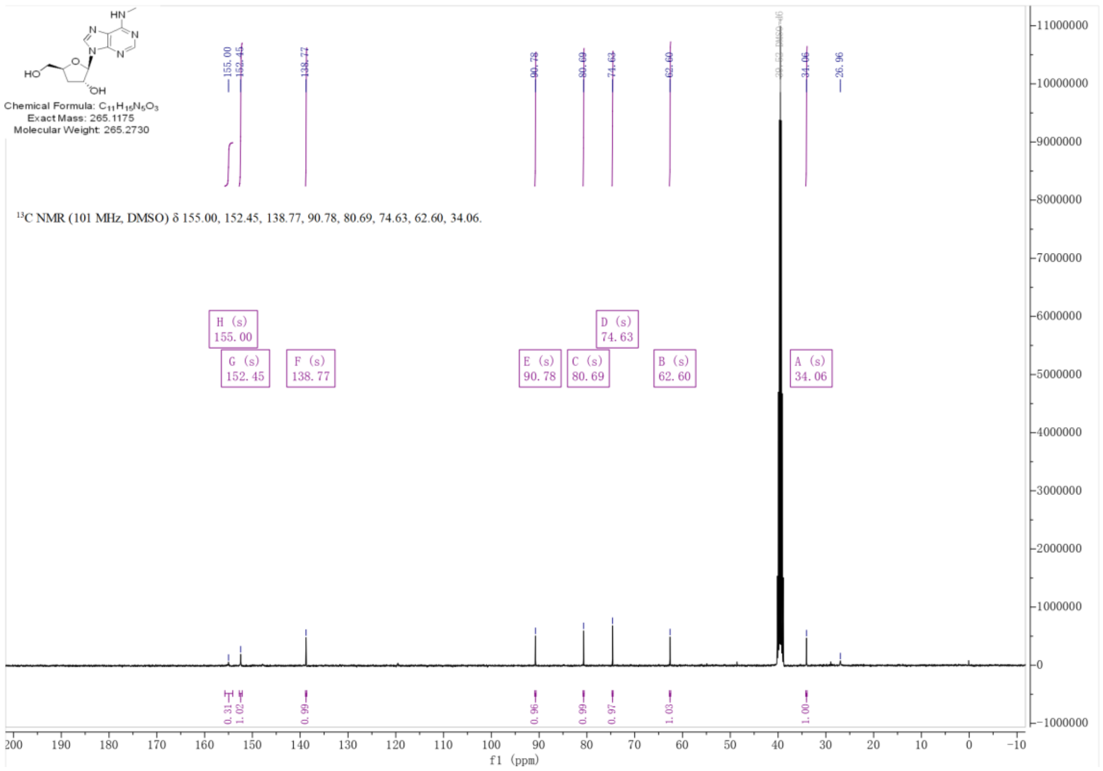

Figure S5. **^{1}H NMR** and **^{13}C NMR** spectrum of compound **1a**

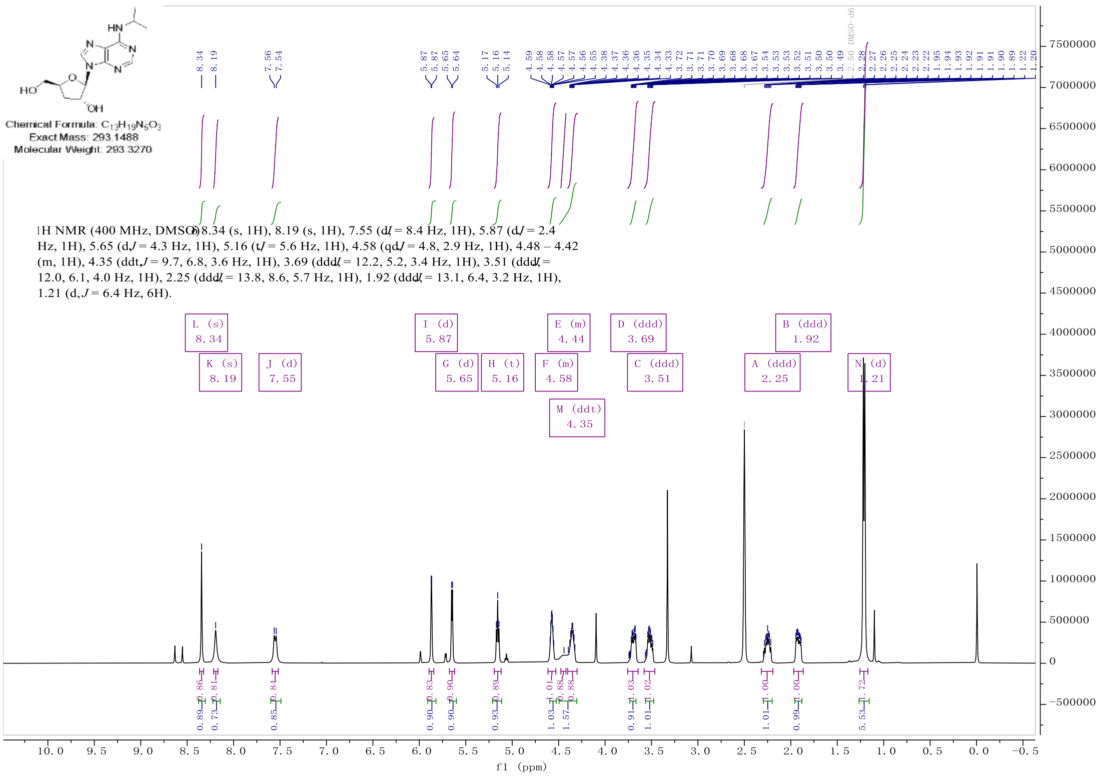

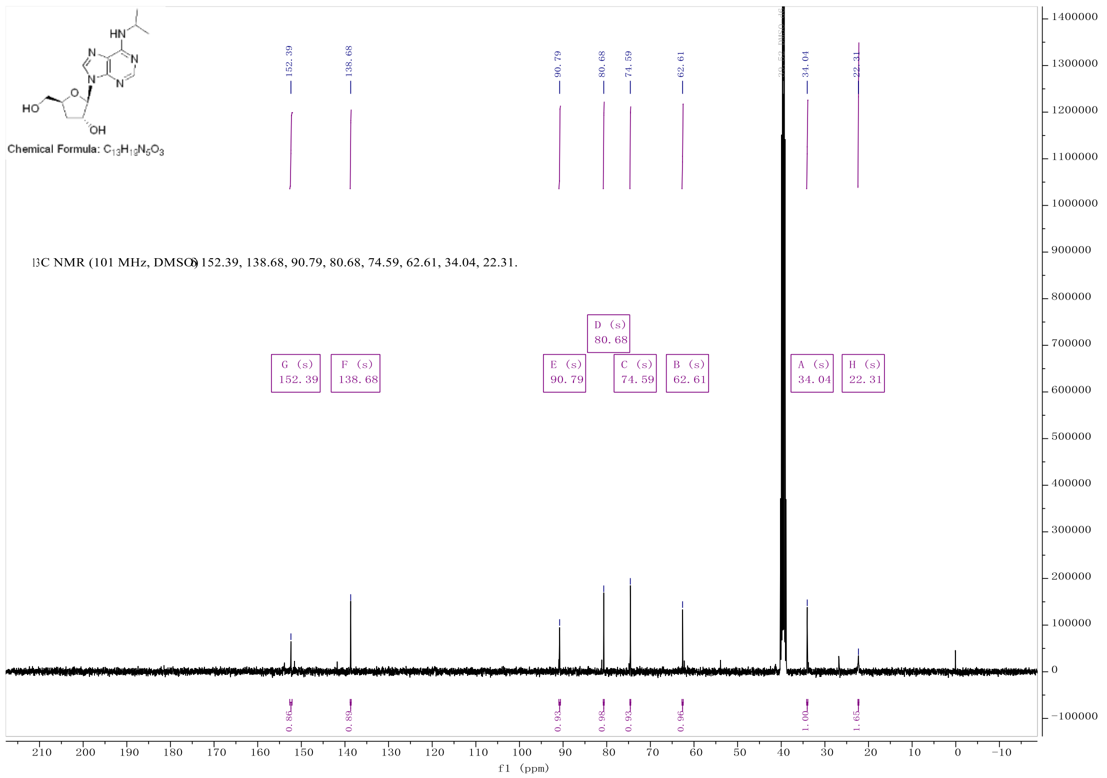

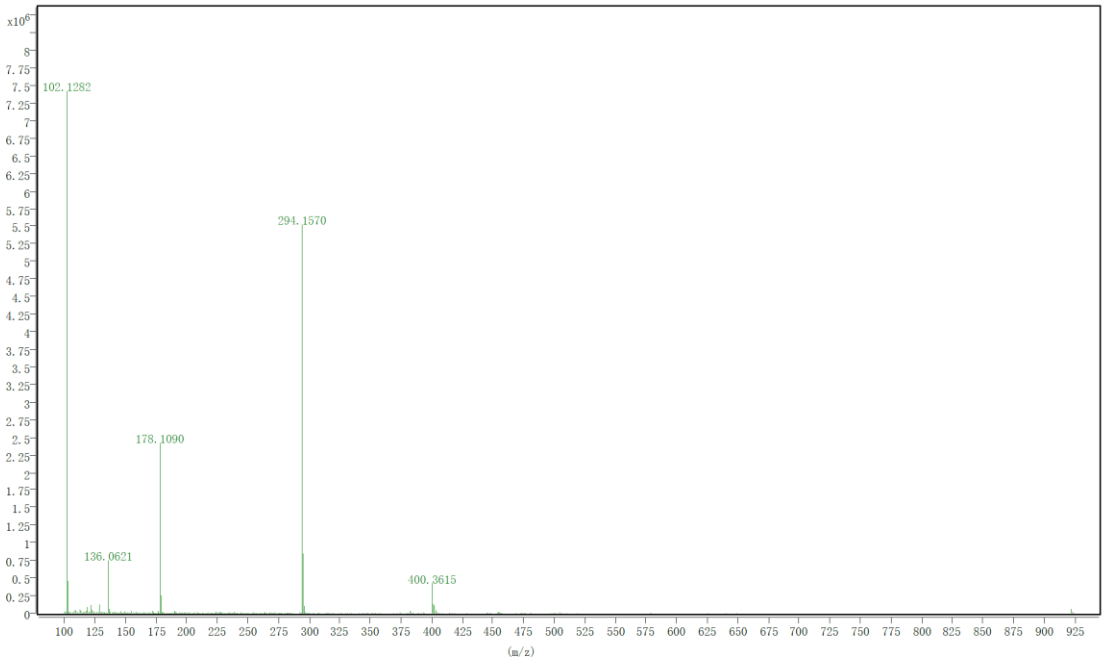

Figure S6. **^{1}H NMR**, **^{13}C NMR** and Mass spectra spectrum of compound **1b**

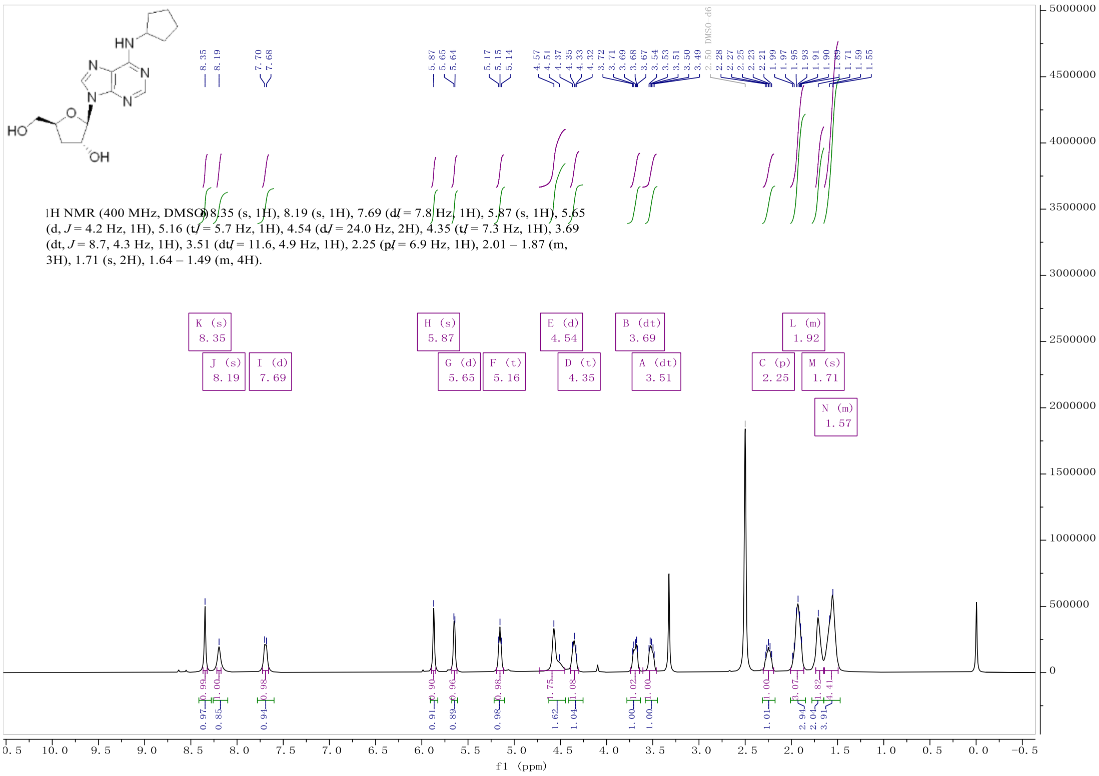
HN
N
N
N
N
HO
O
OH
1H NMR (400 MHz, DMSO) 8.35 (s, 1H), 8.19 (s, 1H), 7.69 (d, J = 7.8 Hz, 1H), 5.87 (s, 1H), 5.65 (d, J = 4.2 Hz, 1H), 5.16 (t, J = 5.7 Hz, 1H), 4.54 (d, J = 24.0 Hz, 2H), 4.35 (t, J = 7.3 Hz, 1H), 3.69 (dt, J = 8.7, 4.3 Hz, 1H), 3.51 (dt, J = 11.6, 4.9 Hz, 1H), 2.25 (p, J = 6.9 Hz, 1H), 2.01 – 1.87 (m, 3H), 1.71 (s, 2H), 1.64 – 1.49 (m, 4H).
K (s) 8.35
J (s) 8.19
I (d) 7.69
H (s) 5.87
G (d) 5.65
F (t) 5.16
E (d) 4.54
D (t) 4.35
B (dt) 3.69
A (dt) 3.51
C (p) 2.25
L (m) 1.92
M (s) 1.71
N (m) 1.57
DMSO-d6
f1 (ppm)

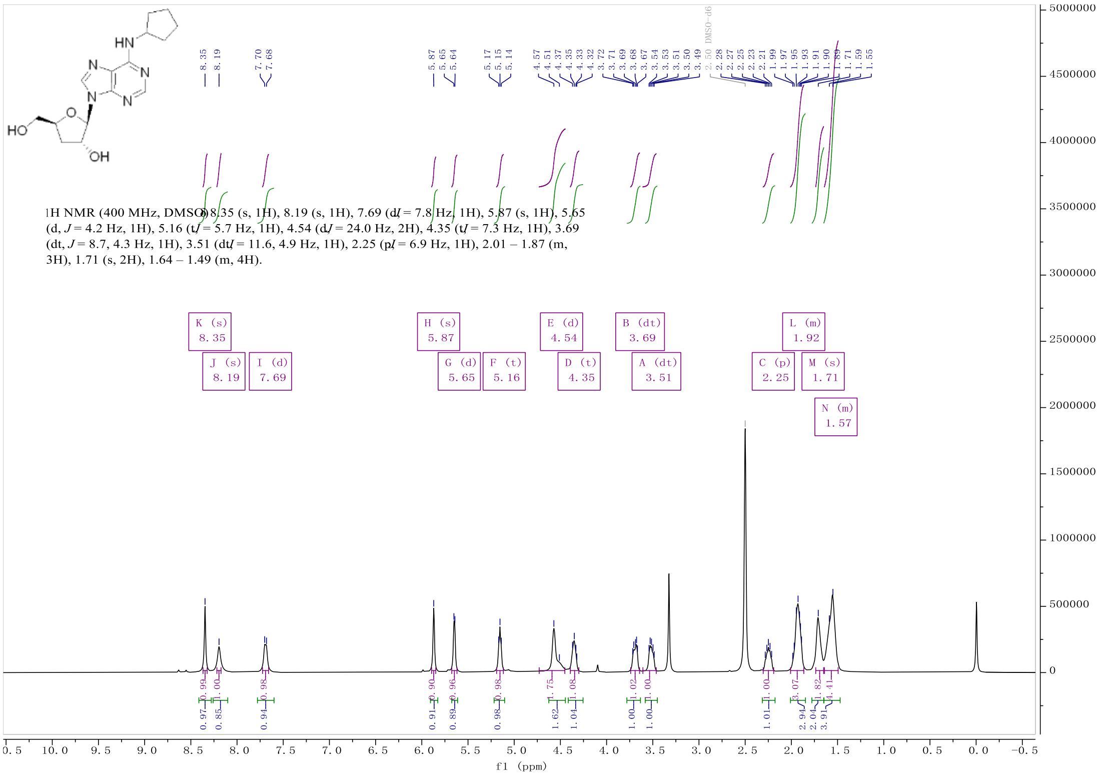
HN
N
N
N
N
HO
O
OH
1H NMR (400 MHz, DMSO) 8.35 (s, 1H), 8.19 (s, 1H), 7.69 (d, J = 7.8 Hz, 1H), 5.87 (s, 1H), 5.65 (d, J = 4.2 Hz, 1H), 5.16 (t, J = 5.7 Hz, 1H), 4.54 (d, J = 24.0 Hz, 2H), 4.35 (t, J = 7.3 Hz, 1H), 3.69 (dt, J = 8.7, 4.3 Hz, 1H), 3.51 (dt, J = 11.6, 4.9 Hz, 1H), 2.25 (p, J = 6.9 Hz, 1H), 2.01 – 1.87 (m, 3H), 1.71 (s, 2H), 1.64 – 1.49 (m, 4H).
K (s) 8.35
J (s) 8.19
I (d) 7.69
H (s) 5.87
G (d) 5.65
F (t) 5.16
E (d) 4.54
D (t) 4.35
B (dt) 3.69
A (dt) 3.51
C (p) 2.25
L (m) 1.92
M (s) 1.71
N (m) 1.57
DMSO-d6
f1 (ppm)

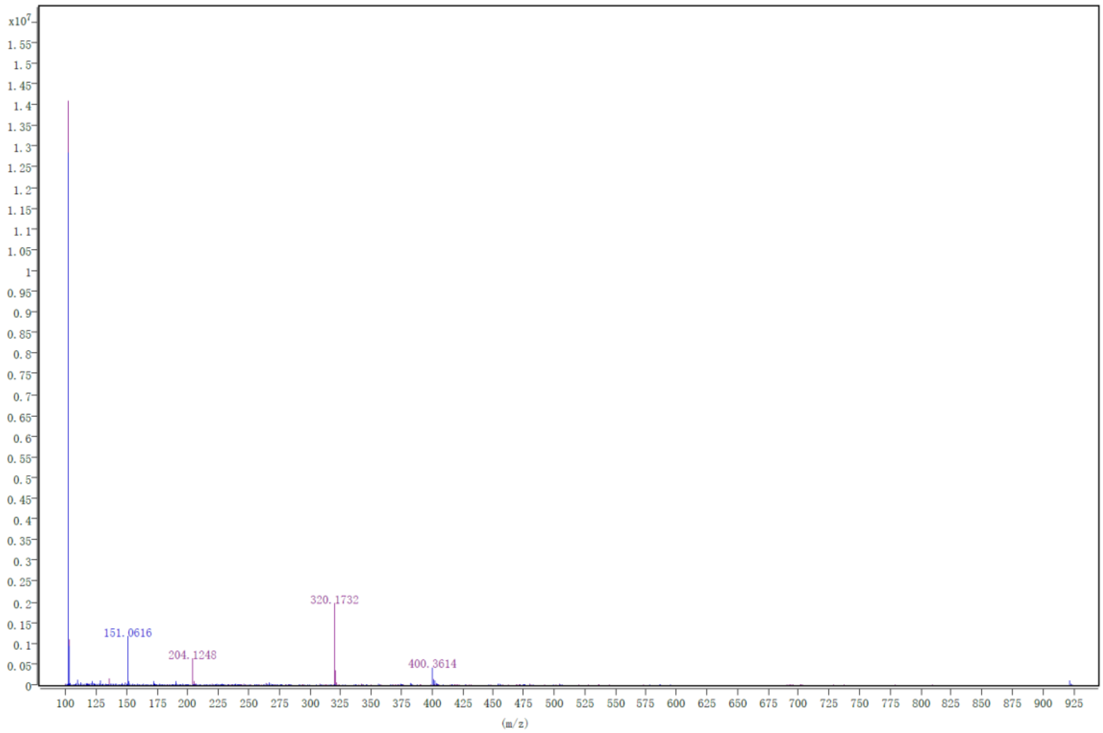

Figure S7. [1]**H NMR**, [13]**C NMR** and Mass spectra spectrum of compound **1c**

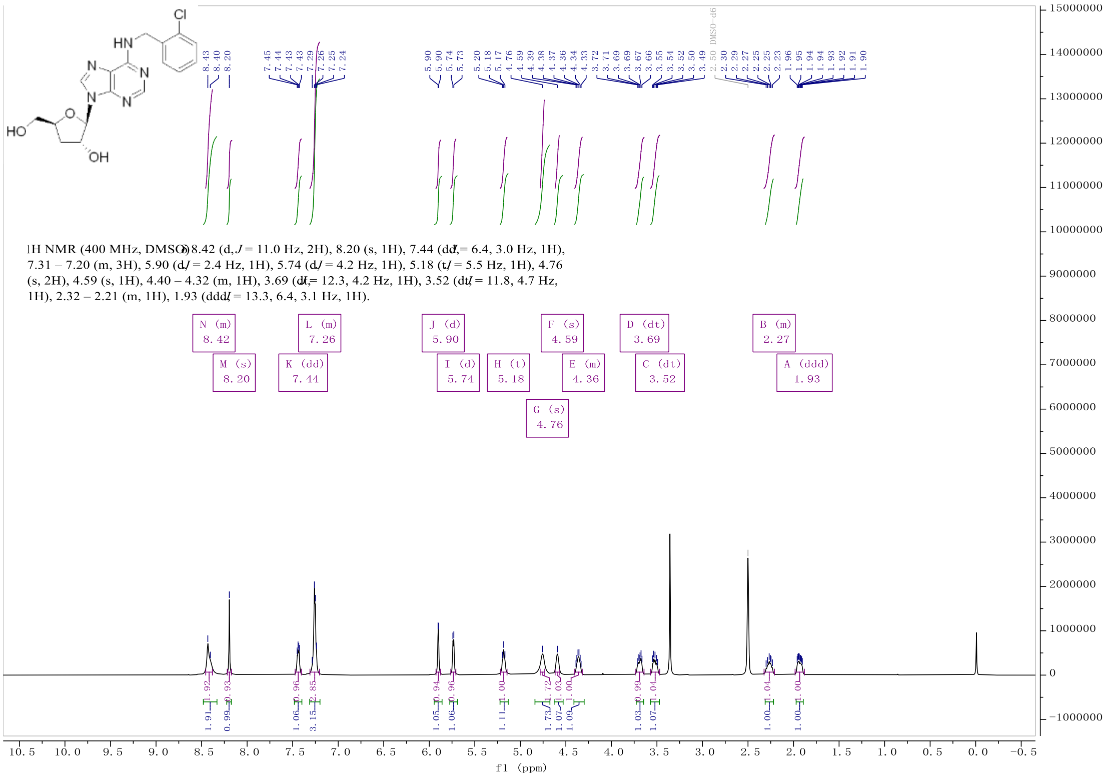

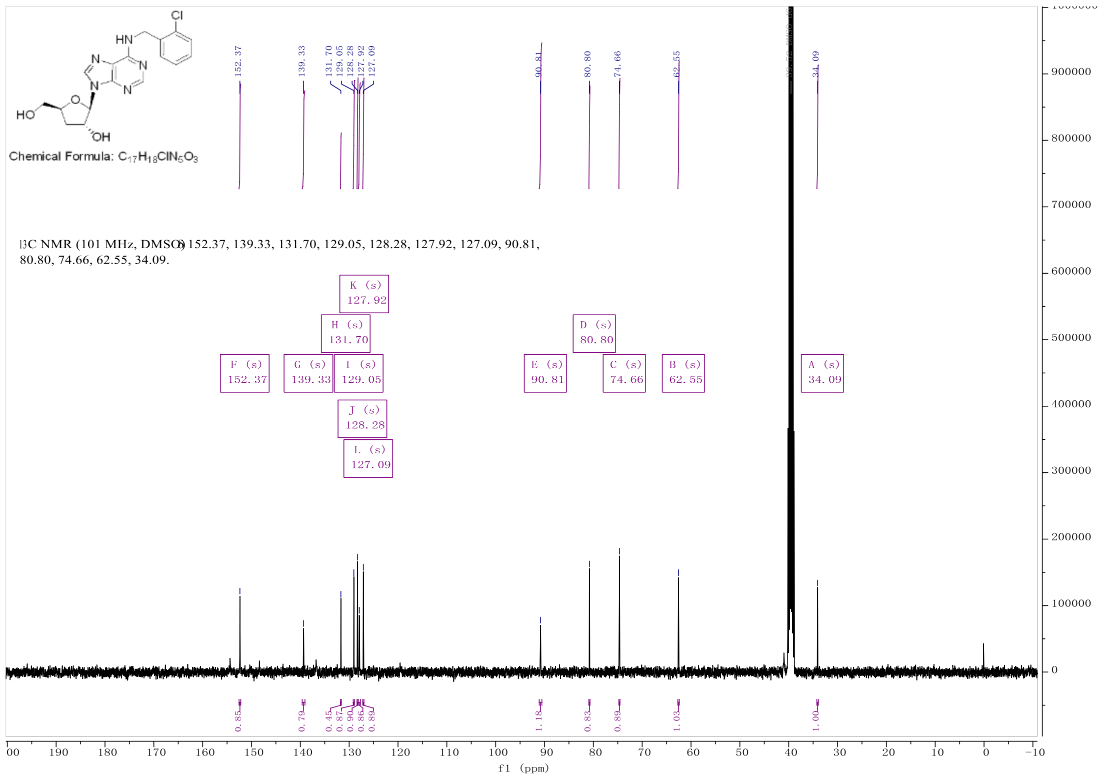

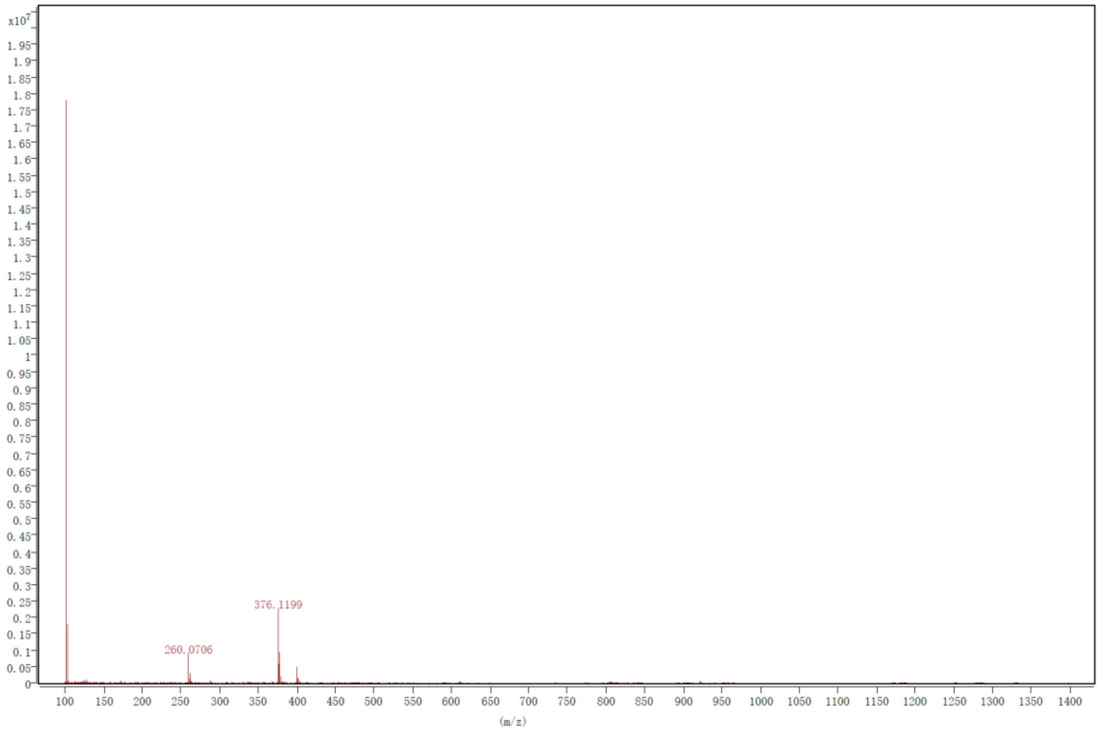

Figure S8. **^{1}H NMR**, **^{13}C NMR** and Mass spectra spectrum of compound **1d**

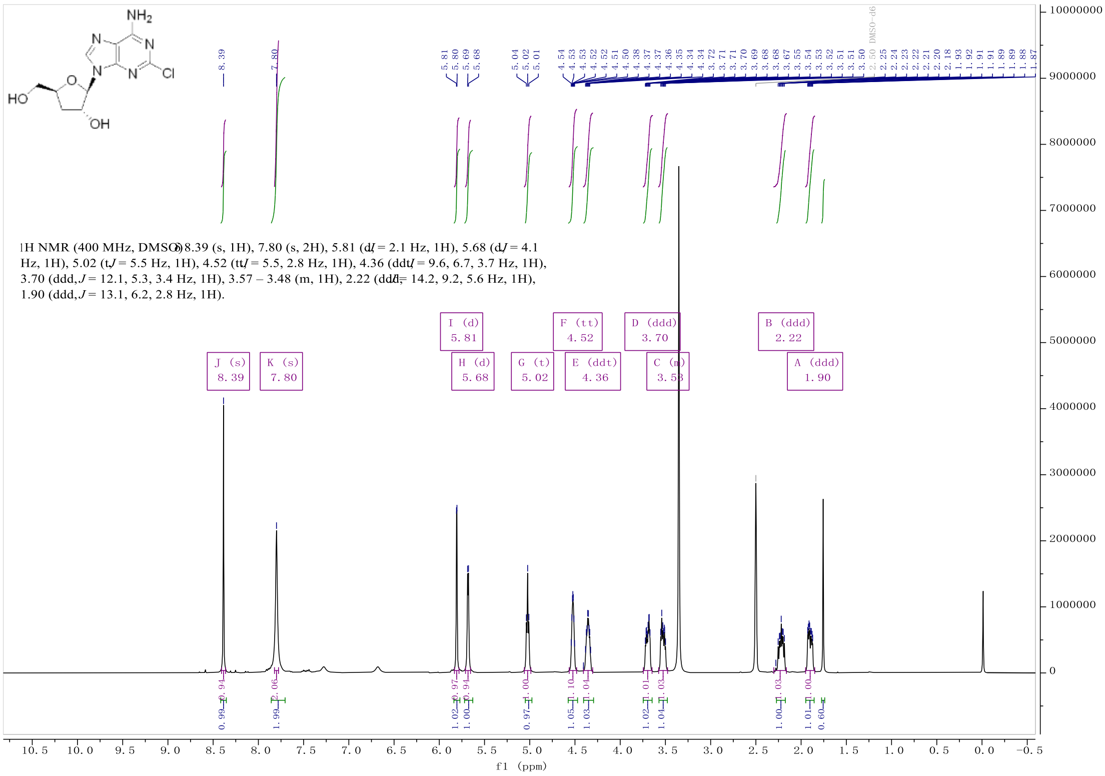
NH2
N
N
N
N
Cl
HO
O
OH
1H NMR (400 MHz, DMSO) δ 8.39 (s, 1H), 7.80 (s, 2H), 5.81 (d, J = 2.1 Hz, 1H), 5.68 (d, J = 4.1 Hz, 1H), 5.02 (t, J = 5.5 Hz, 1H), 4.52 (tt, J = 5.5, 2.8 Hz, 1H), 4.36 (ddt, J = 9.6, 6.7, 3.7 Hz, 1H), 3.70 (ddd, J = 12.1, 5.3, 3.4 Hz, 1H), 3.57 – 3.48 (m, 1H), 2.22 (ddd, J = 14.2, 9.2, 5.6 Hz, 1H), 1.90 (ddd, J = 13.1, 6.2, 2.8 Hz, 1H).
f1 (ppm)

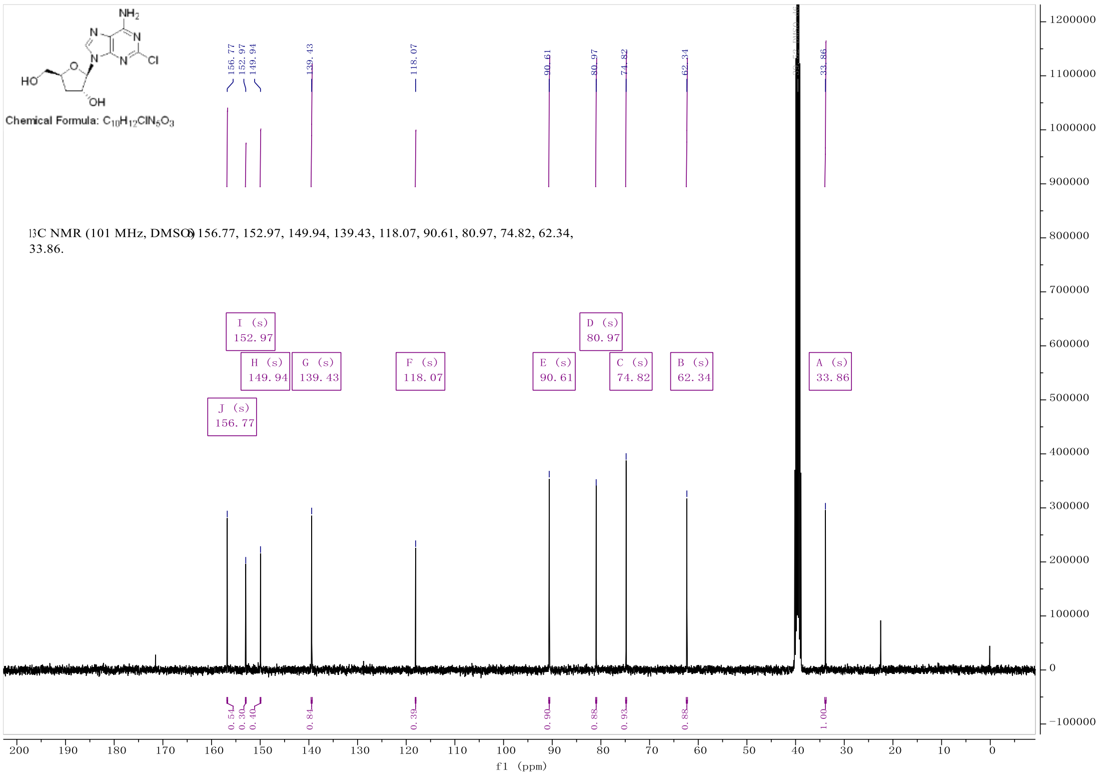
NH2
N
N
N
N
Cl
HO
O
OH
Chemical Formula: C10H12ClN5O3
13C NMR (101 MHz, DMSO) δ 156.77, 152.97, 149.94, 139.43, 118.07, 90.61, 80.97, 74.82, 62.34, 33.86.
f1 (ppm)

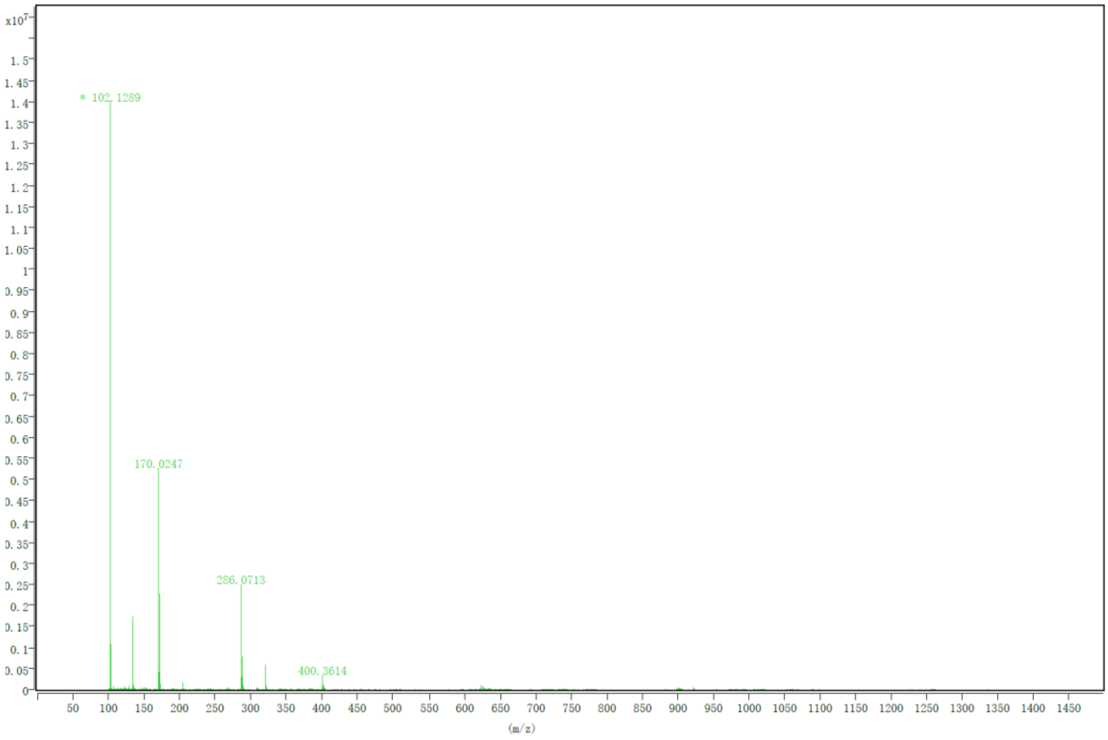

Figure S9. **^{1}H NMR**, **^{13}C NMR** and Mass spectra spectrum of compound **1e**

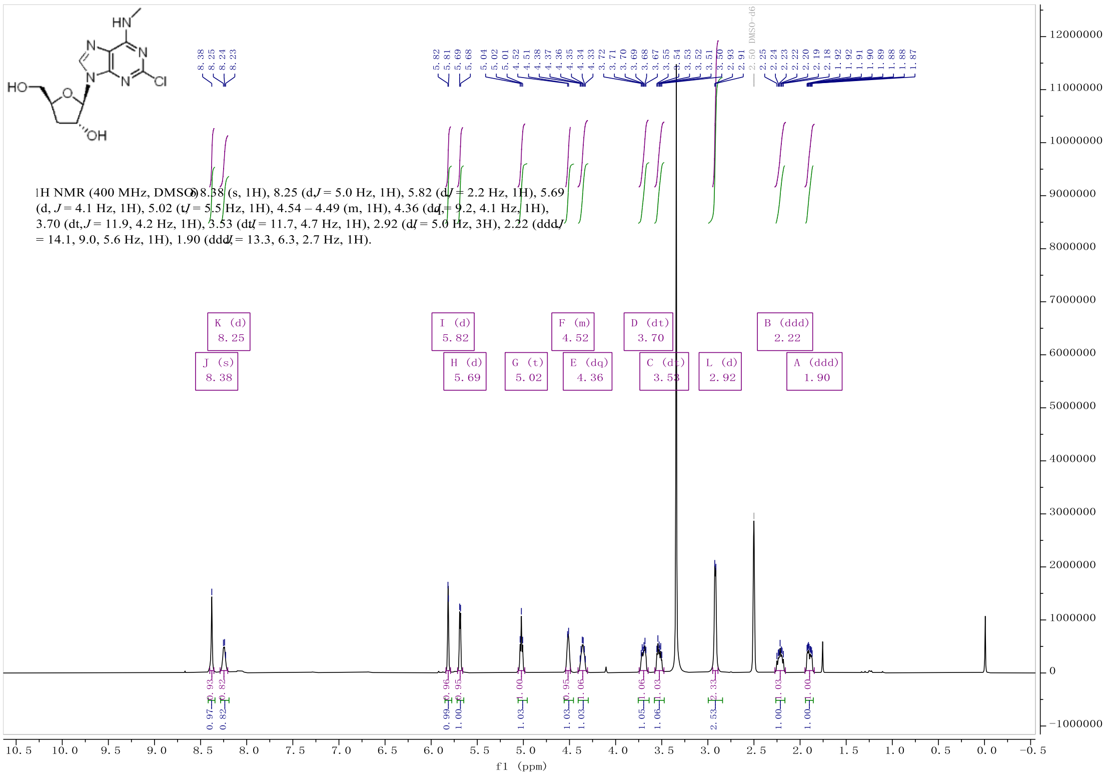

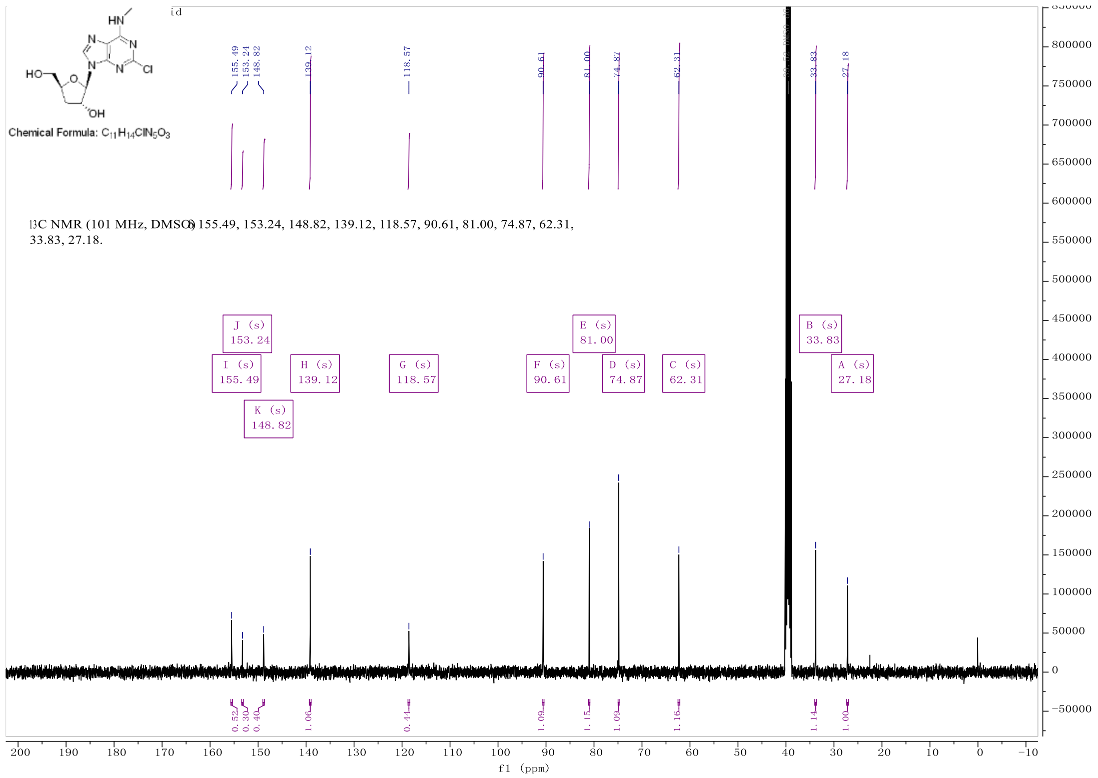

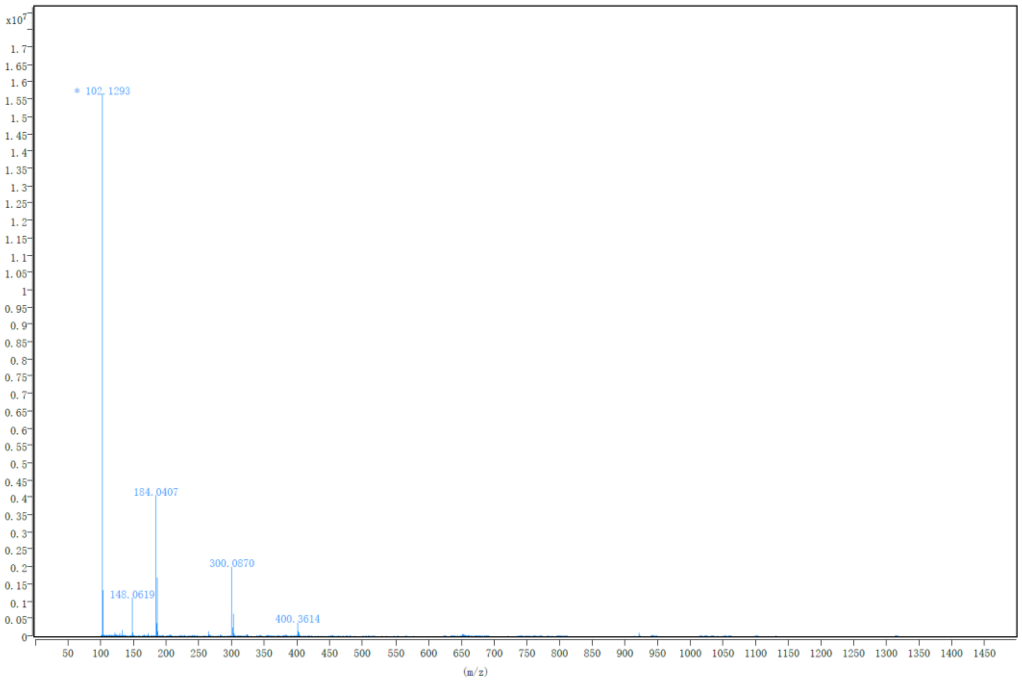

Figure S10. ^{1}**H NMR**, ^{13}**C NMR** and Mass spectra spectrum of compound **1f**

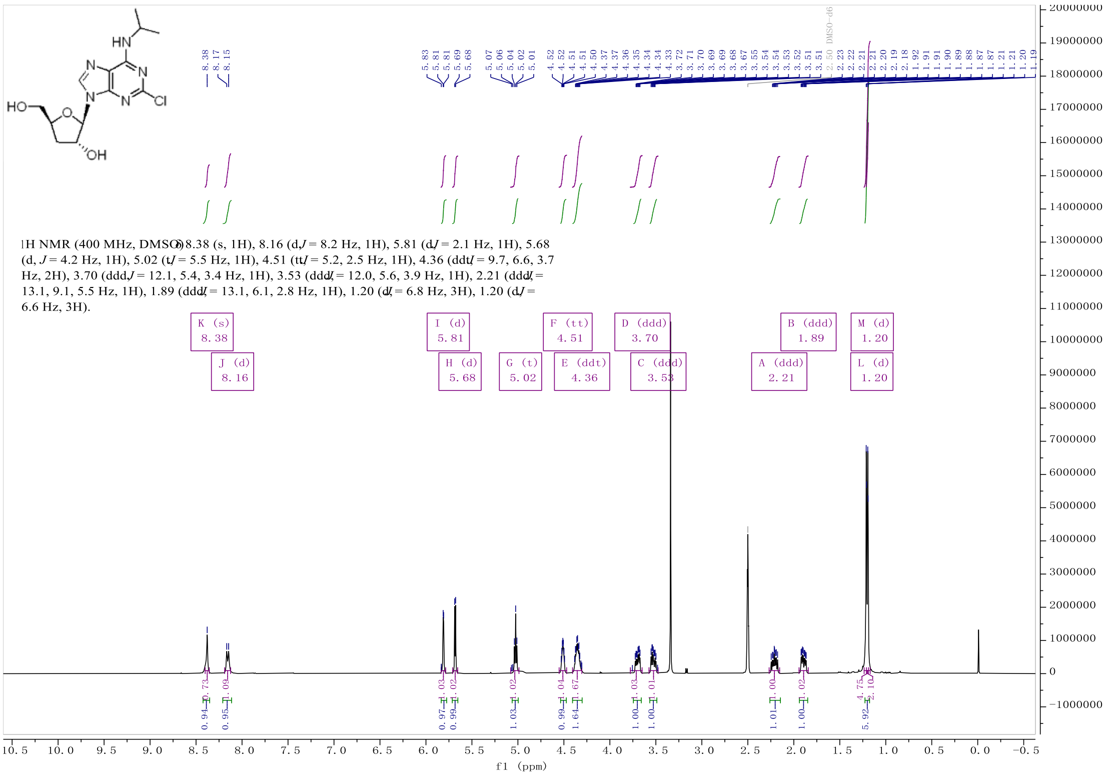
HN
N
N
N
N
Cl
HO
O
OH
DMSO-d6
1H NMR (400 MHz, DMSO) δ 8.38 (s, 1H), 8.16 (d, J = 8.2 Hz, 1H), 5.81 (d, J = 2.1 Hz, 1H), 5.68 (d, J = 4.2 Hz, 1H), 5.02 (t, J = 5.5 Hz, 1H), 4.51 (tt, J = 5.2, 2.5 Hz, 1H), 4.36 (ddt, J = 9.7, 6.6, 3.7 Hz, 2H), 3.70 (ddd, J = 12.1, 5.4, 3.4 Hz, 1H), 3.53 (ddd, J = 12.0, 5.6, 3.9 Hz, 1H), 2.21 (ddd, J = 13.1, 9.1, 5.5 Hz, 1H), 1.89 (ddd, J = 13.1, 6.1, 2.8 Hz, 1H), 1.20 (d, J = 6.8 Hz, 3H), 1.20 (d, J = 6.6 Hz, 3H).
K (s) 8.38
J (d) 8.16
I (d) 5.81
H (d) 5.68
G (t) 5.02
F (tt) 4.51
E (ddt) 4.36
D (ddd) 3.70
C (ddd) 3.53
A (ddd) 2.21
B (ddd) 1.89
M (d) 1.20
L (d) 1.20
f1 (ppm)

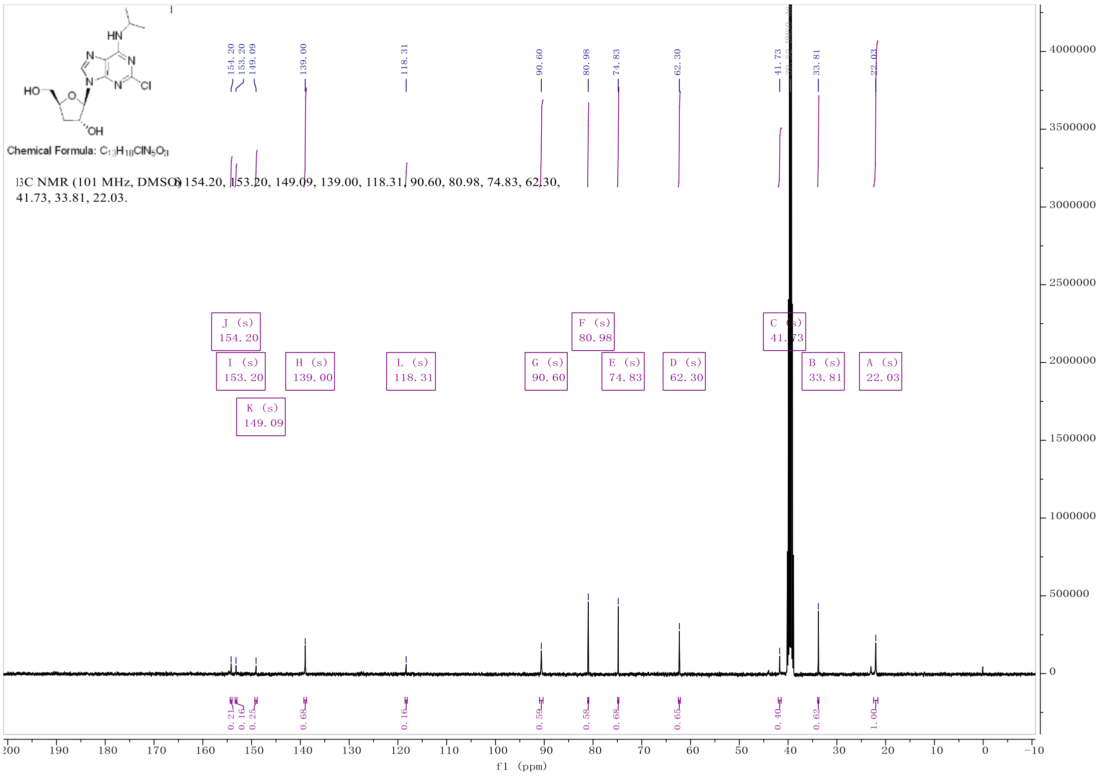
HN
N
N
N
N
Cl
HO
O
OH
Chemical Formula: C13H18ClN5O3
13C NMR (101 MHz, DMSO) δ 154.20, 153.20, 149.09, 139.00, 118.31, 90.60, 80.98, 74.83, 62.30, 41.73, 33.81, 22.03.
J (s) 154.20
I (s) 153.20
K (s) 149.09
H (s) 139.00
L (s) 118.31
G (s) 90.60
F (s) 80.98
E (s) 74.83
D (s) 62.30
C (s) 41.73
B (s) 33.81
A (s) 22.03
f1 (ppm)

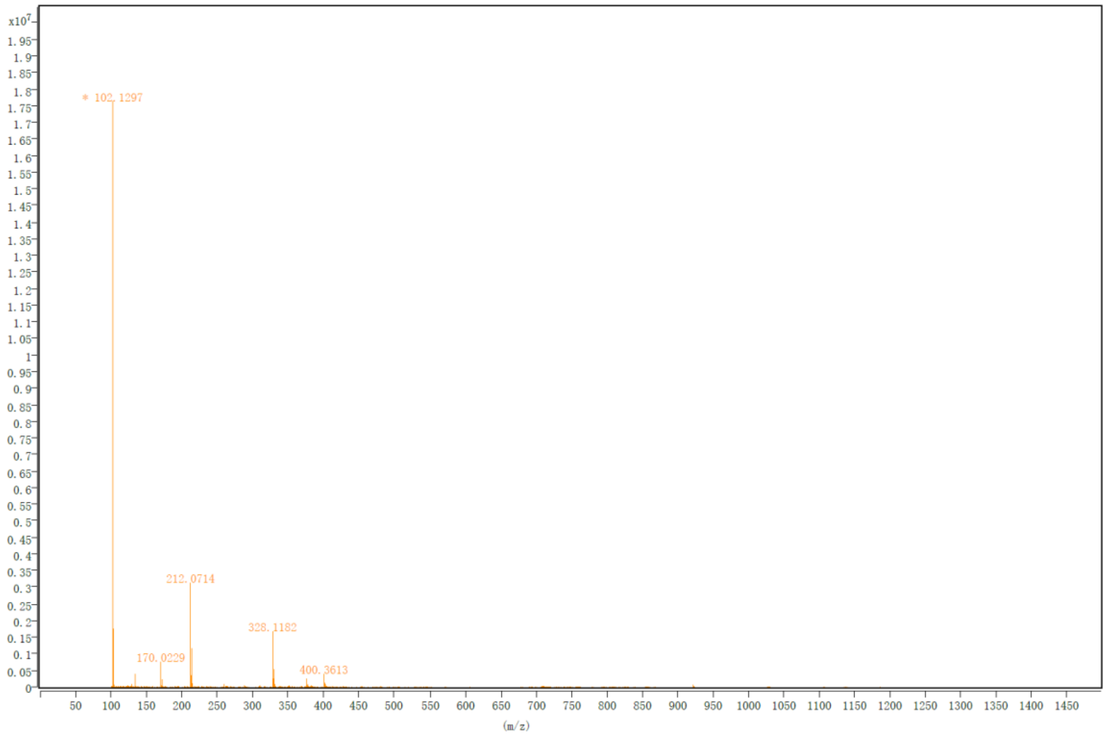

Figure S11. **^{1}H NMR**, **^{13}C NMR** and Mass spectra spectrum of compound **1g**

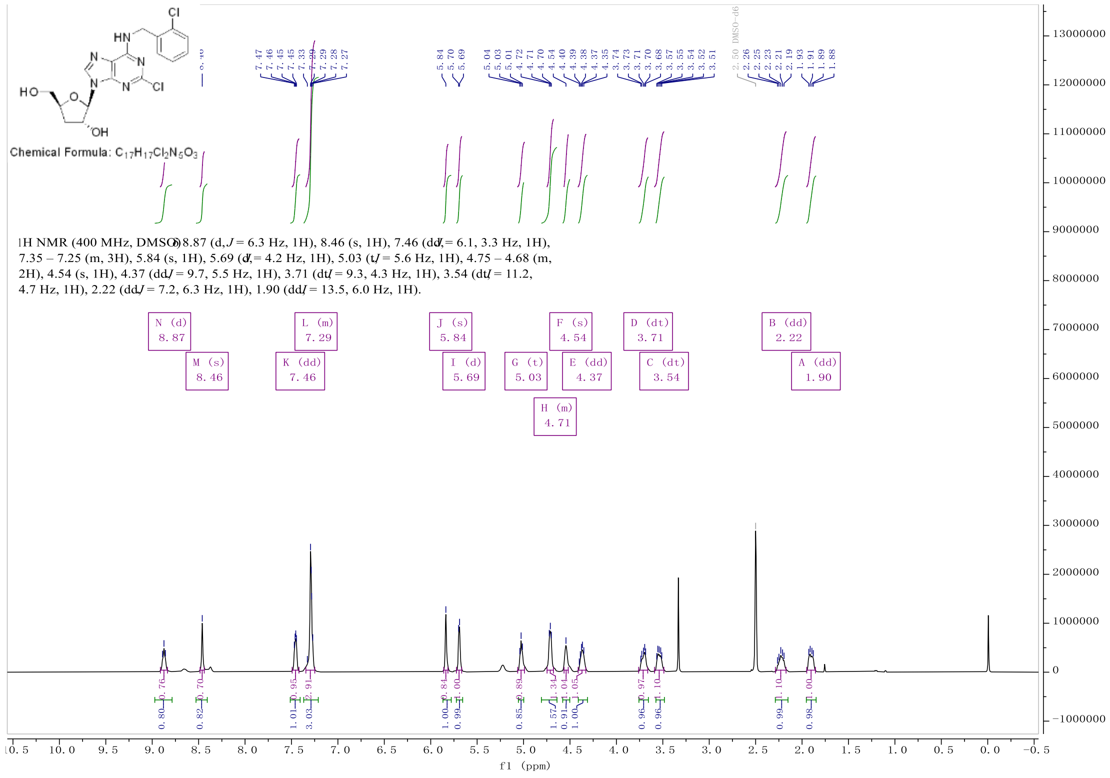

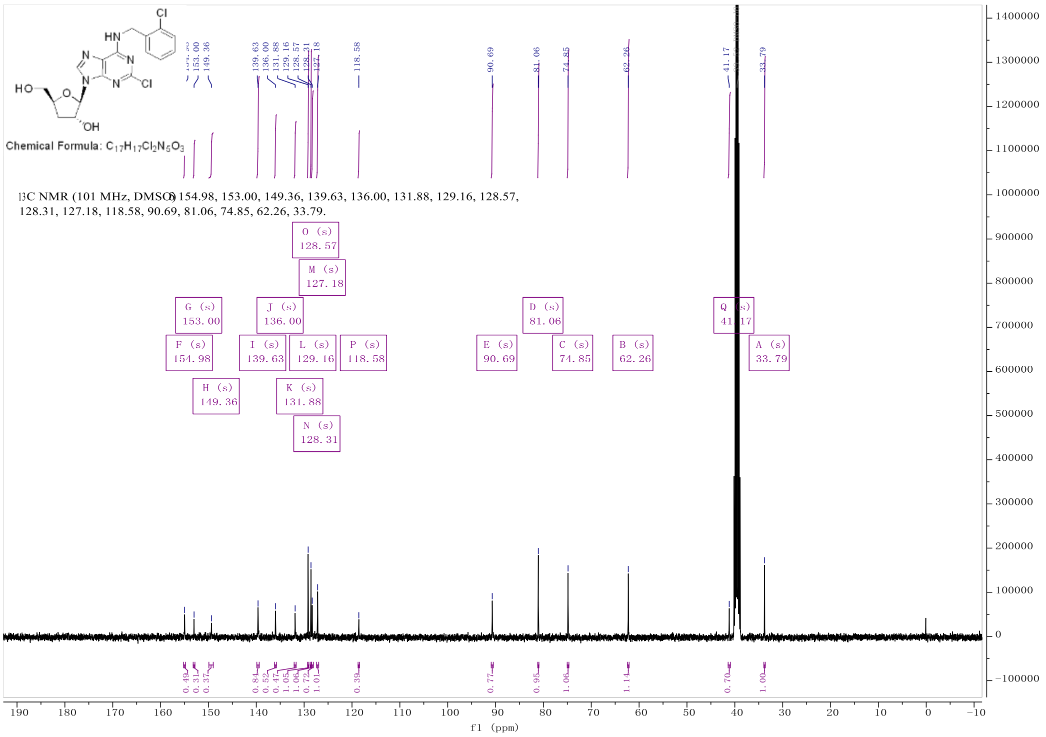

Figure S12. ^{1}**H NMR**, ^{13}**C NMR** and Mass spectra spectrum of compound **1h**

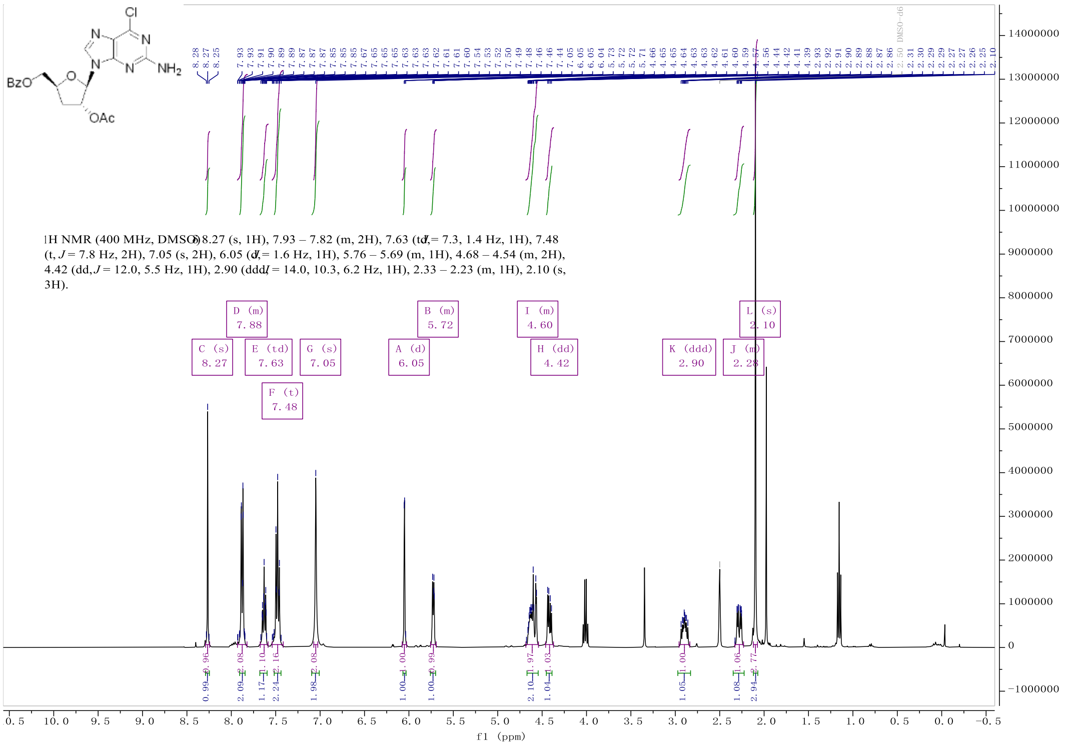

Figure S13. ^{1}**H NMR** spectrum of compound **12**

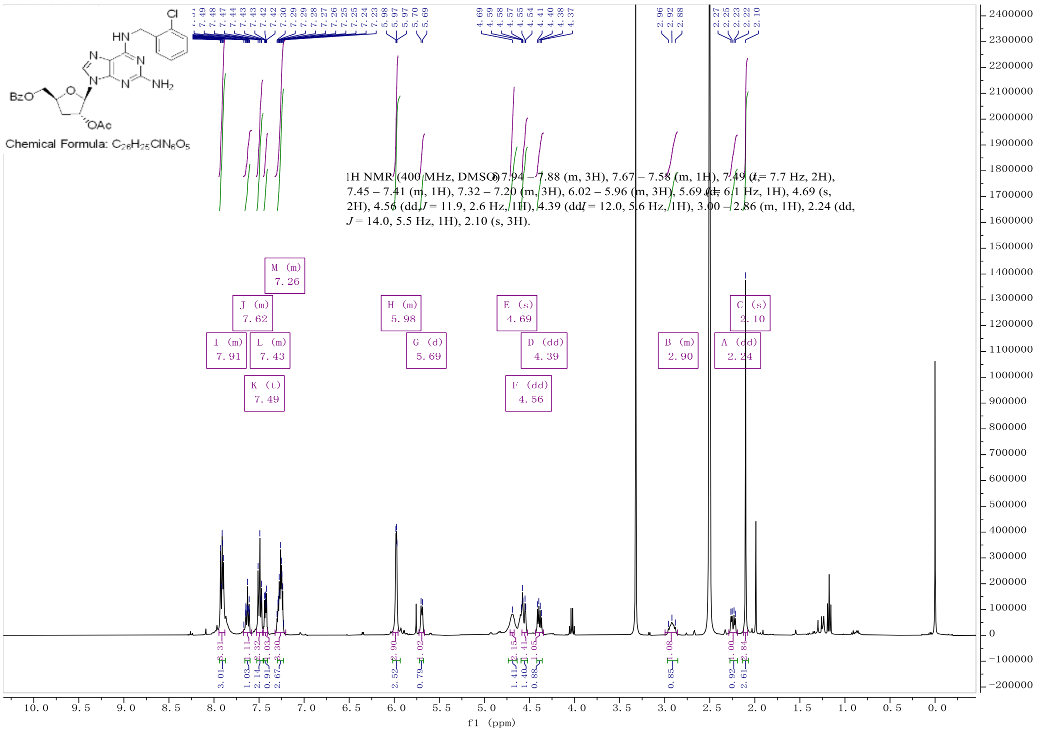

Figure S14. **^{1}H NMR** spectrum of compound **13**

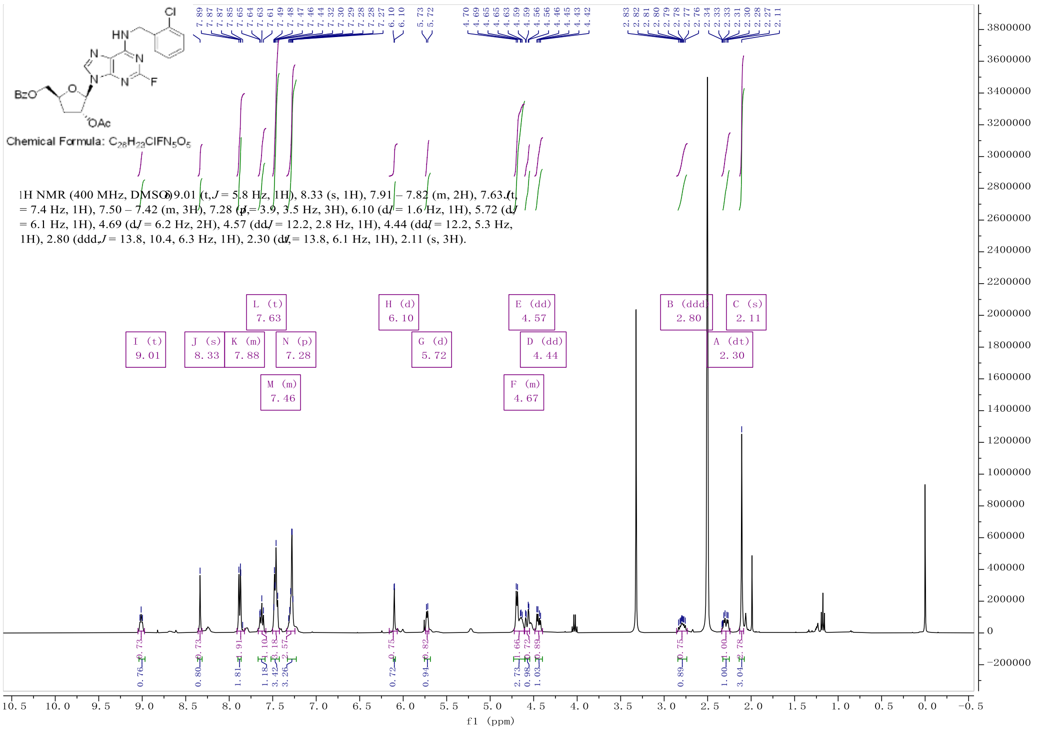

Figure S15. **^{1}H NMR** spectrum of compound **14a**

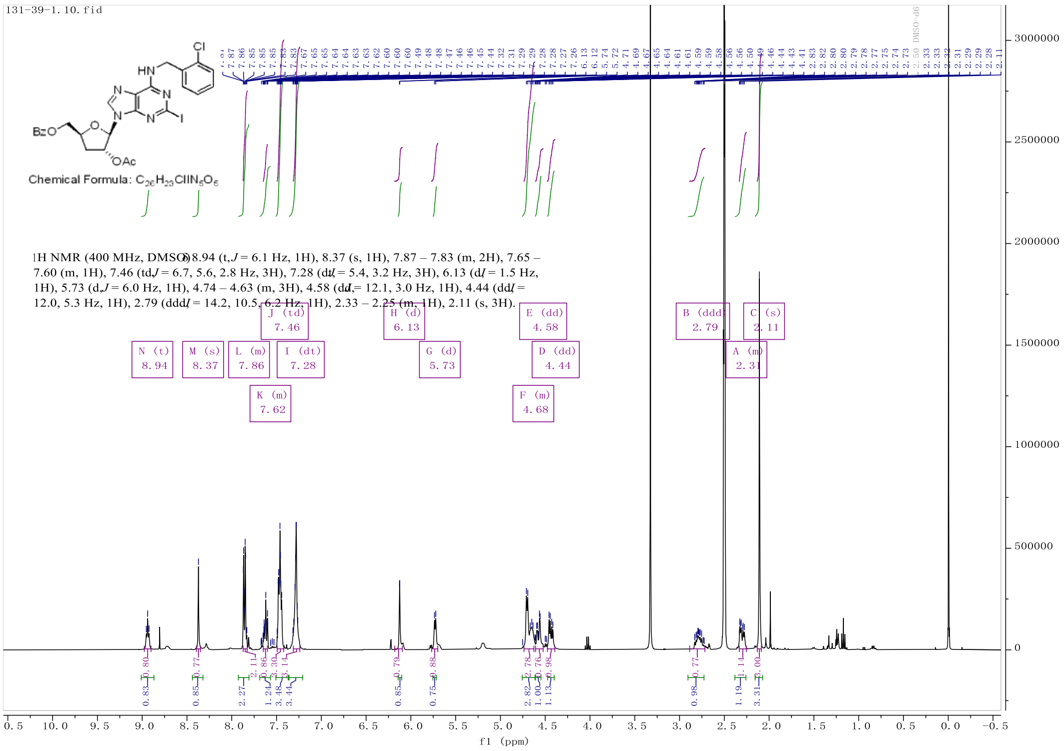

Figure S16. [1]**H NMR** spectrum of compound **14b**

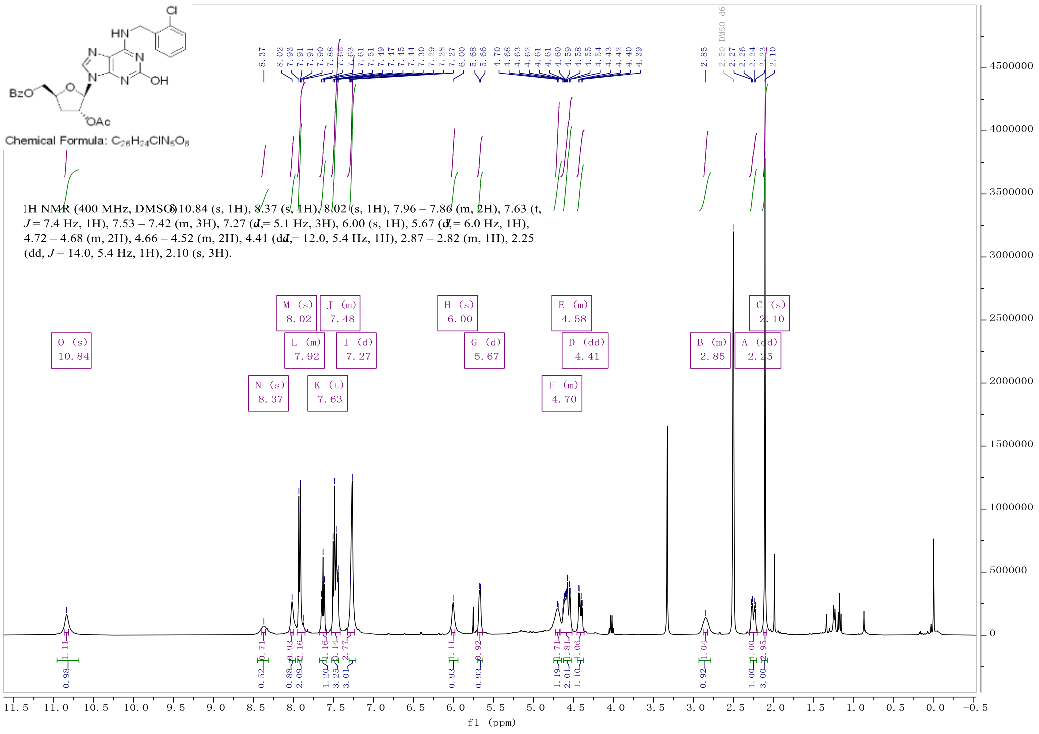

Figure S17. [1]**H NMR** spectrum of compound **14c**

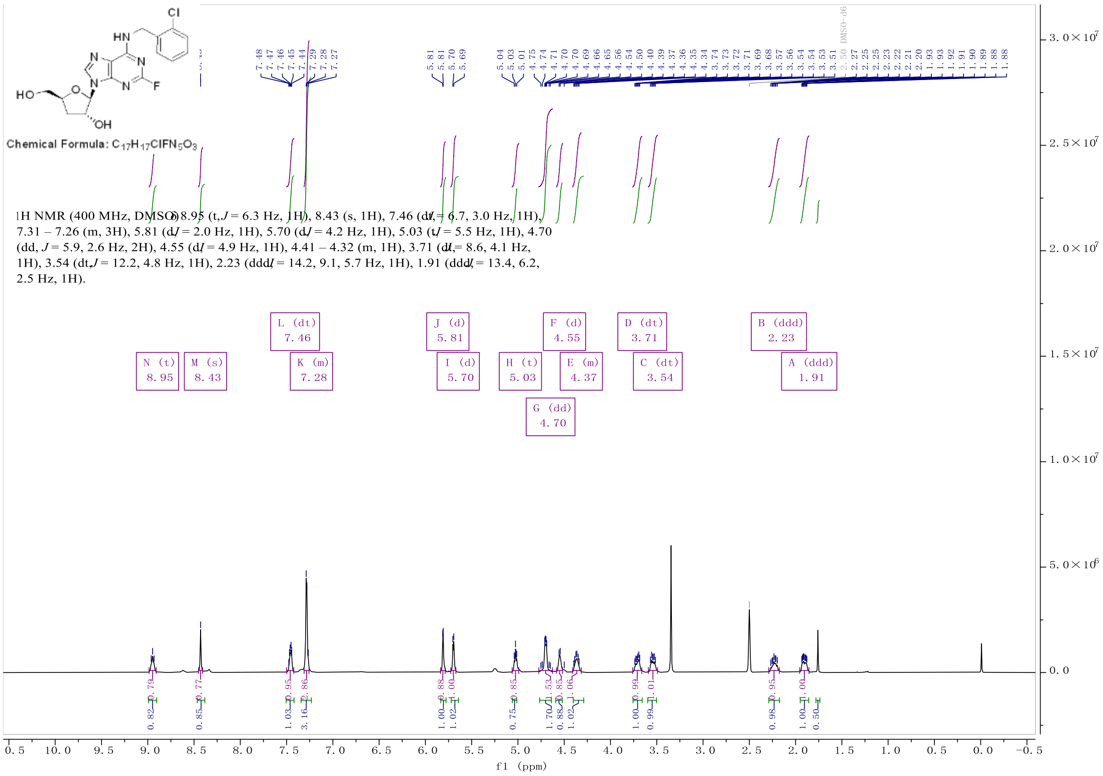
Cl
HN
N
N
N
N
F
HO
O
OH
Chemical Formula: $C_{17}H_{17}ClFN_5O_3$
1H NMR (400 MHz, DMSO) δ 8.95 (t, J = 6.3 Hz, 1H), 8.43 (s, 1H), 7.46 (dt, J = 6.7, 3.0 Hz, 1H), 7.31 – 7.26 (m, 3H), 5.81 (d, J = 2.0 Hz, 1H), 5.70 (d, J = 4.2 Hz, 1H), 5.03 (t, J = 5.5 Hz, 1H), 4.70 (dd, J = 5.9, 2.6 Hz, 2H), 4.55 (d, J = 4.9 Hz, 1H), 4.41 – 4.32 (m, 1H), 3.71 (dt, J = 8.6, 4.1 Hz, 1H), 3.54 (dt, J = 12.2, 4.8 Hz, 1H), 2.23 (ddd, J = 14.2, 9.1, 5.7 Hz, 1H), 1.91 (ddd, J = 13.4, 6.2, 2.5 Hz, 1H).
L (dt) 7.46
J (d) 5.81
F (d) 4.55
D (dt) 3.71
B (ddd) 2.23
N (t) 8.95
M (s) 8.43
K (m) 7.28
I (d) 5.70
H (t) 5.03
E (m) 4.37
C (dt) 3.54
A (ddd) 1.91
G (dd) 4.70
DMSO-d6
f1 (ppm)

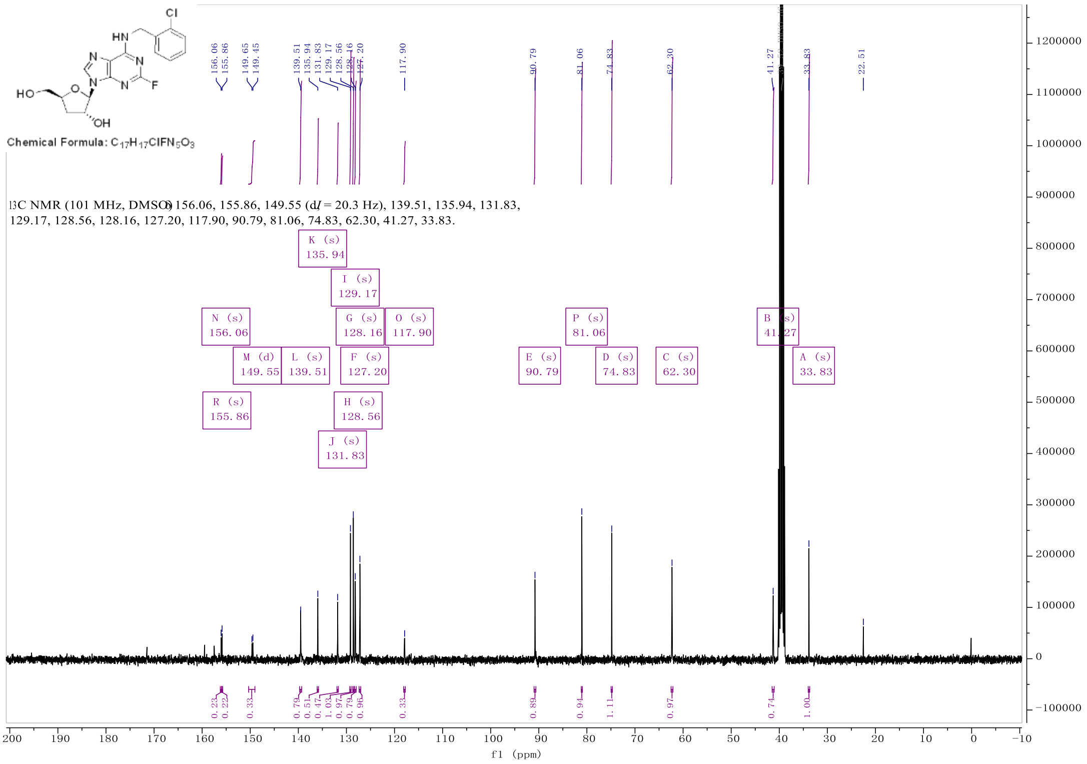
Cl
HN
N
N
N
N
F
HO
O
OH
Chemical Formula: $C_{17}H_{17}ClFN_5O_3$
13C NMR (101 MHz, DMSO) δ 156.06, 155.86, 149.55 (d, J = 20.3 Hz), 139.51, 135.94, 131.83, 129.17, 128.56, 128.16, 127.20, 117.90, 90.79, 81.06, 74.83, 62.30, 41.27, 33.83.
K (s) 135.94
I (s) 129.17
N (s) 156.06
G (s) 128.16
O (s) 117.90
P (s) 81.06
B (s) 41.27
M (d) 149.55
L (s) 139.51
F (s) 127.20
E (s) 90.79
D (s) 74.83
C (s) 62.30
A (s) 33.83
R (s) 155.86
H (s) 128.56
J (s) 131.83
f1 (ppm)

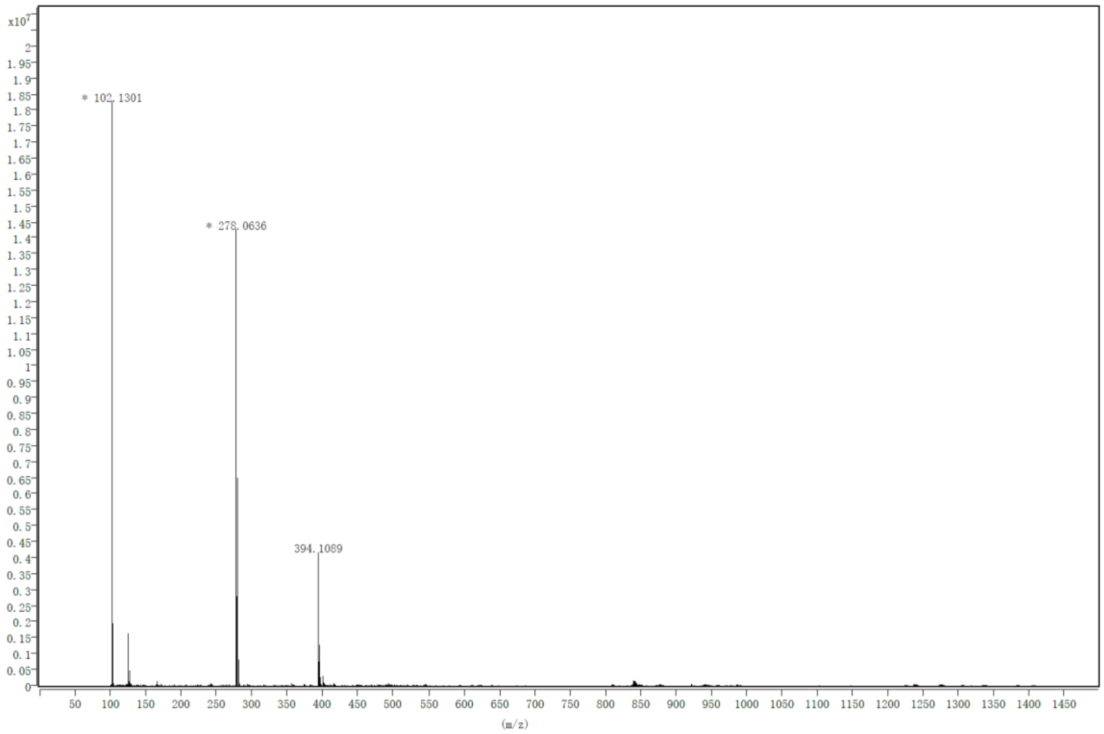

Figure S18. ^{1}**H NMR,** ^{13}**C NMR** and Mass spectra spectrum of compound **1i**

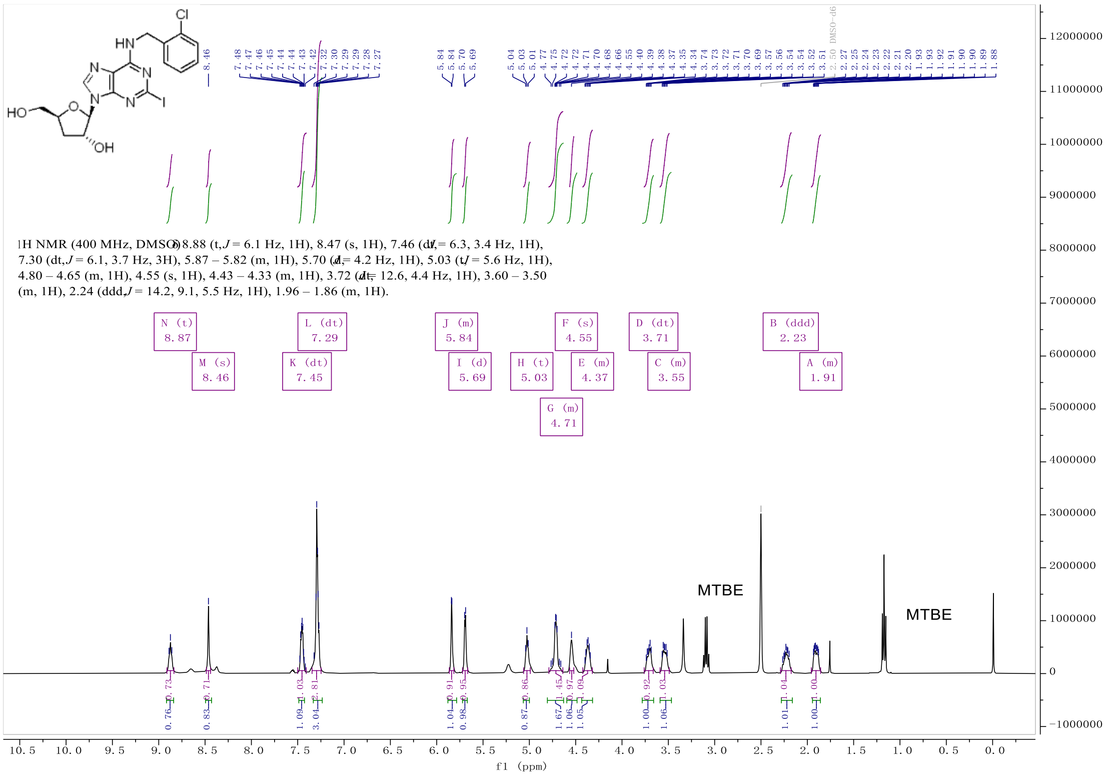

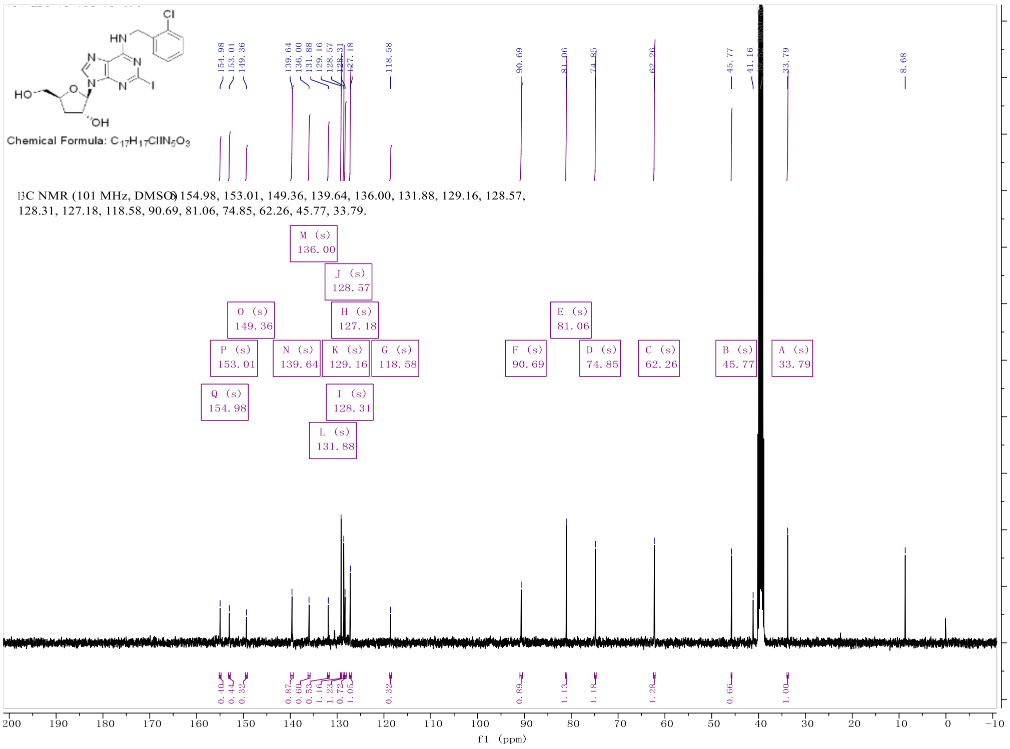

Figure S19. ^{1}**H NMR,** ^{13}**C NMR** and Mass spectra spectrum of compound **1j**

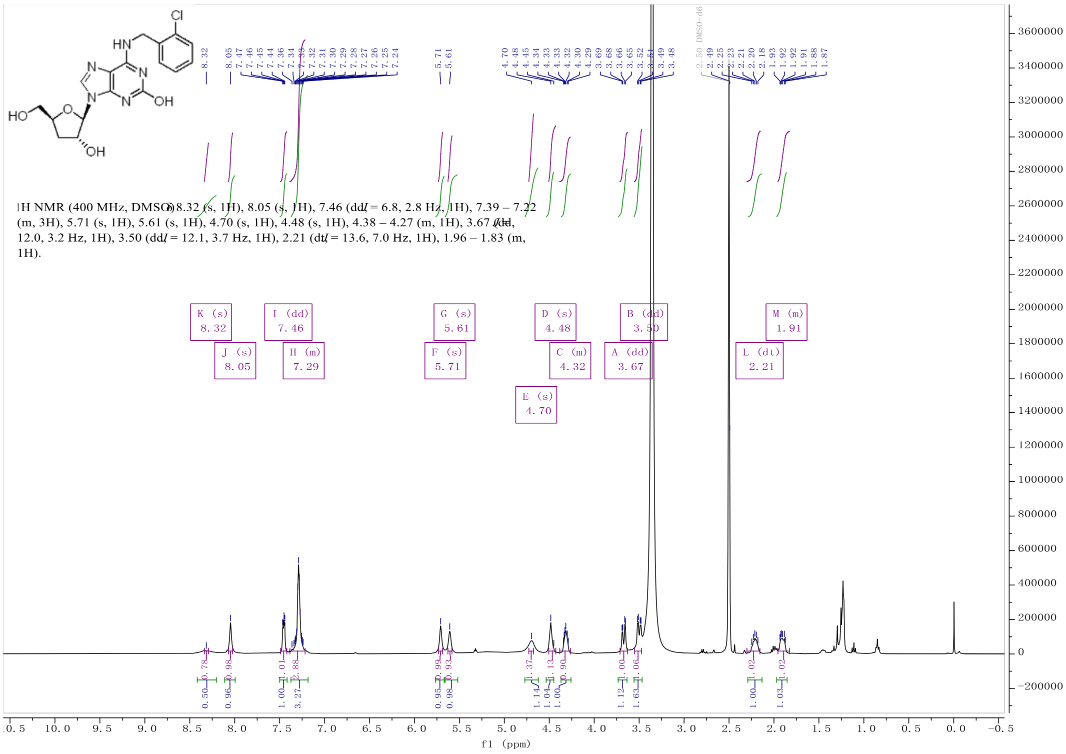

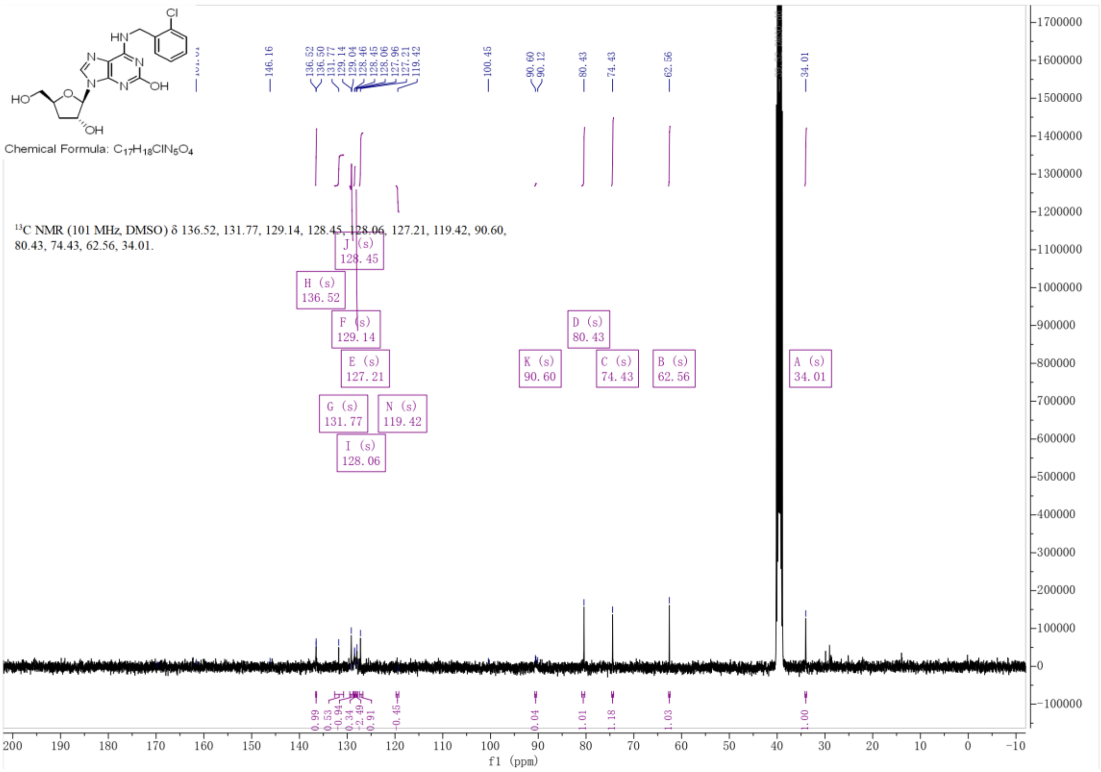

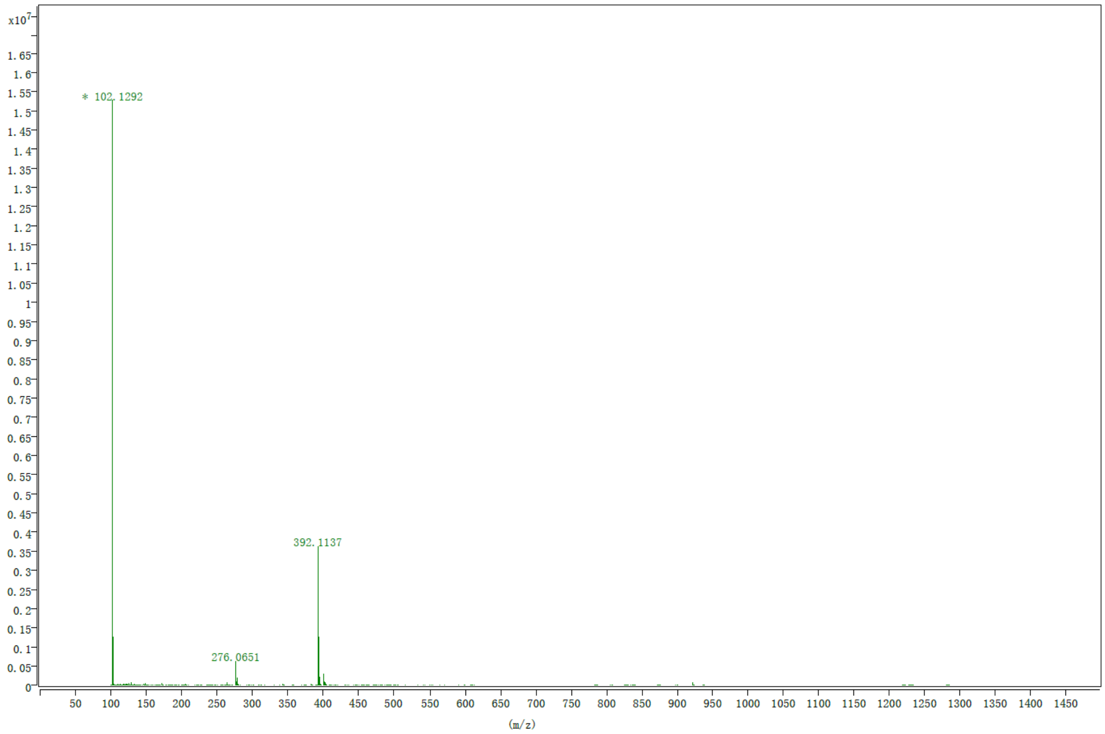

Figure S20. ^{1}**H NMR,** ^{13}**C NMR** and Mass spectra spectrum of compound **1k**

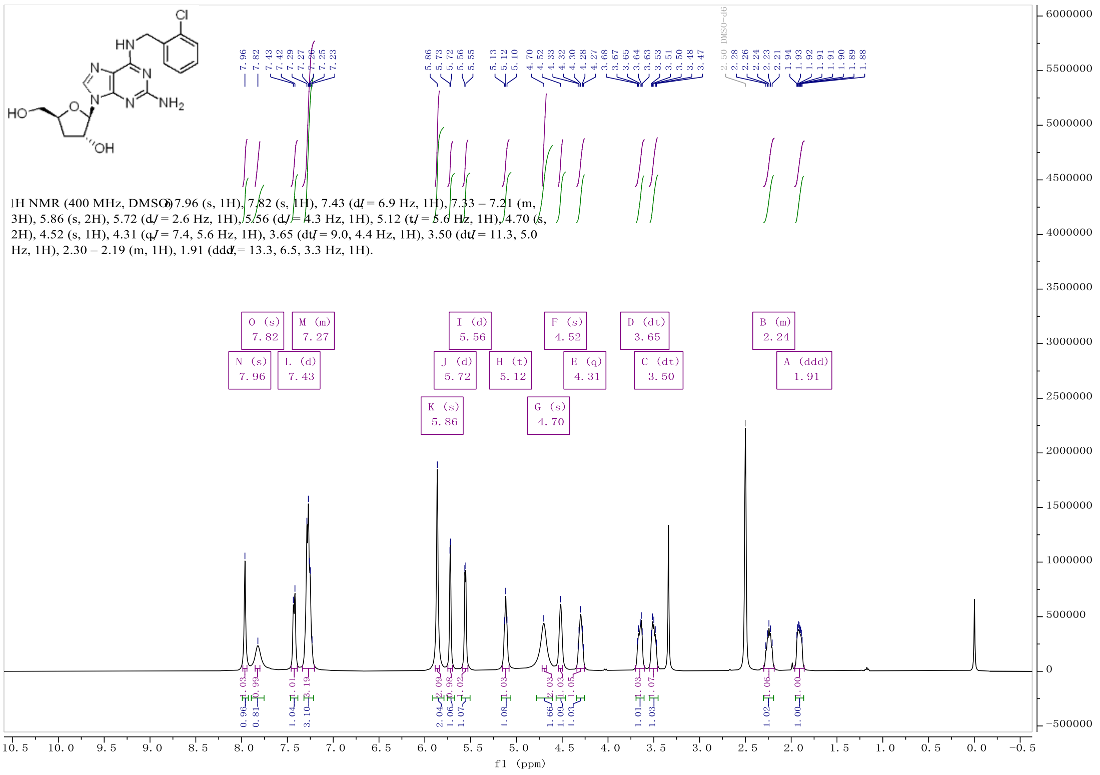

1H NMR (400 MHz, DMSO) δ 7.96 (s, 1H), 7.82 (s, 1H), 7.43 (d, J = 6.9 Hz, 1H), 7.33 – 7.21 (m, 3H), 5.86 (s, 2H), 5.72 (d, J = 2.6 Hz, 1H), 5.56 (d, J = 4.3 Hz, 1H), 5.12 (t, J = 5.6 Hz, 1H), 4.70 (s, 2H), 4.52 (s, 1H), 4.31 (q, J = 7.4, 5.6 Hz, 1H), 3.65 (dt, J = 9.0, 4.4 Hz, 1H), 3.50 (dt, J = 11.3, 5.0 Hz, 1H), 2.30 – 2.19 (m, 1H), 1.91 (ddd, J = 13.3, 6.5, 3.3 Hz, 1H).
f1 (ppm)

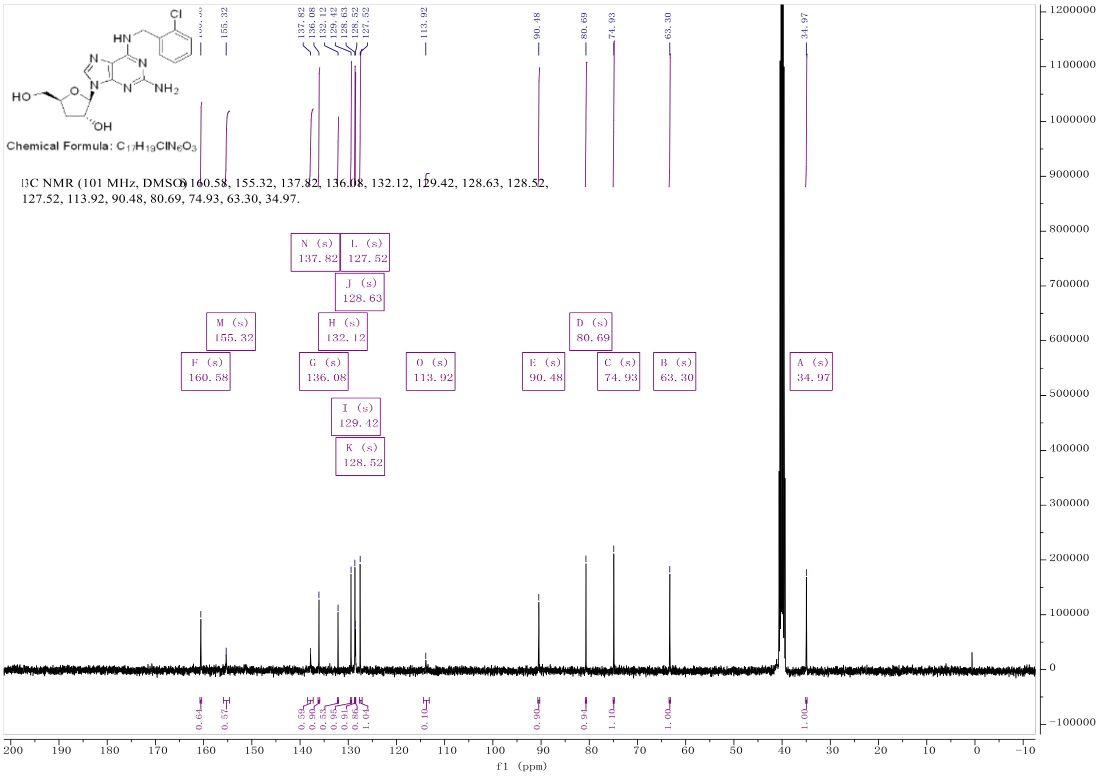

Chemical Formula: $C_{17}H_{19}ClN_6O_3$
13C NMR (101 MHz, DMSO) δ 160.58, 155.32, 137.82, 136.08, 132.12, 129.42, 128.63, 128.52, 127.52, 113.92, 90.48, 80.69, 74.93, 63.30, 34.97.
f1 (ppm)

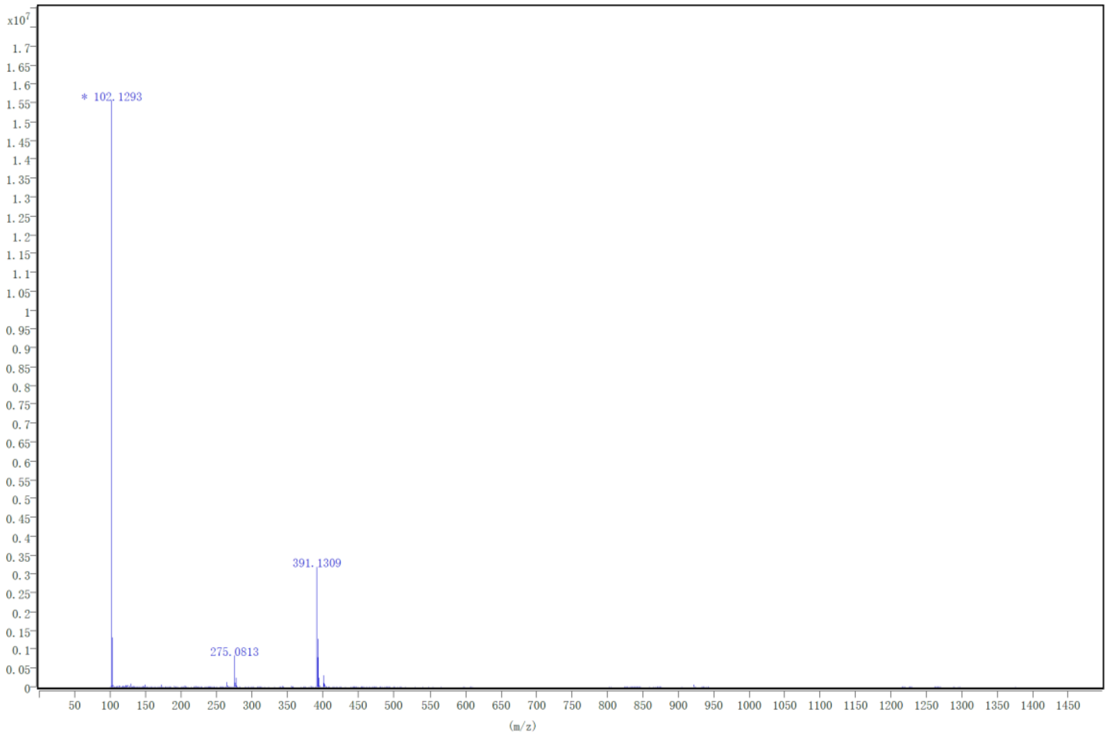

Figure S21. **^{1}H NMR,** **^{13}C NMR** and Mass spectra spectrum of compound **1l**

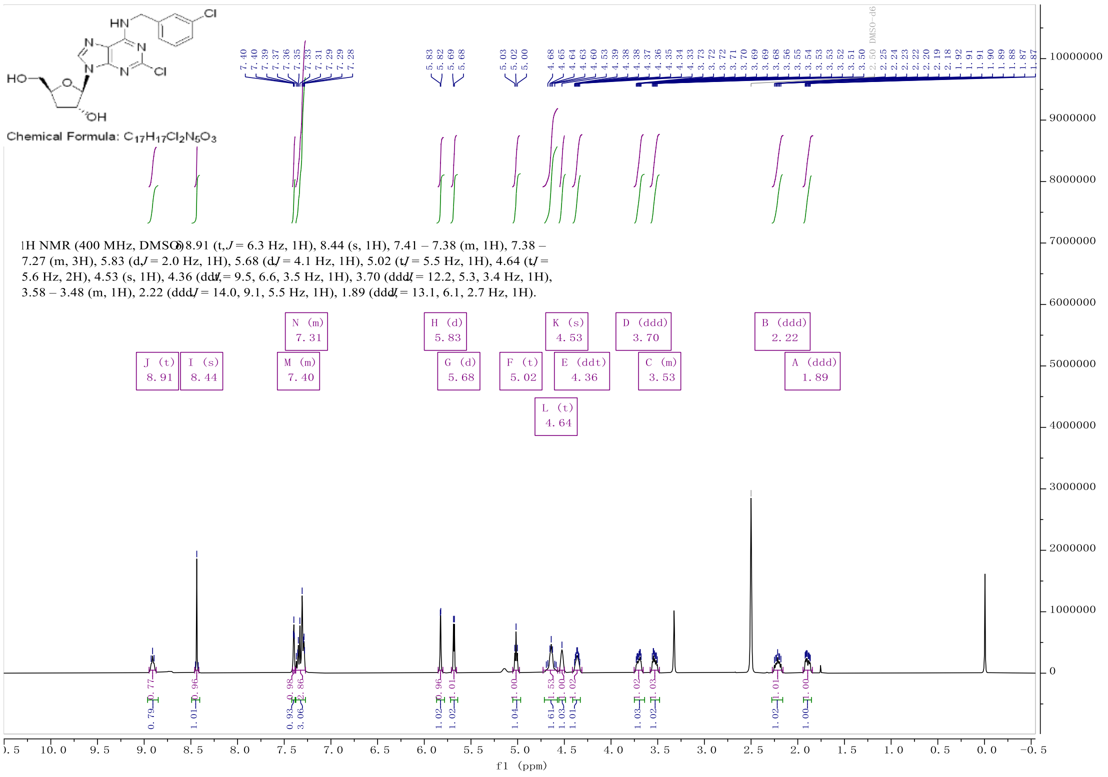

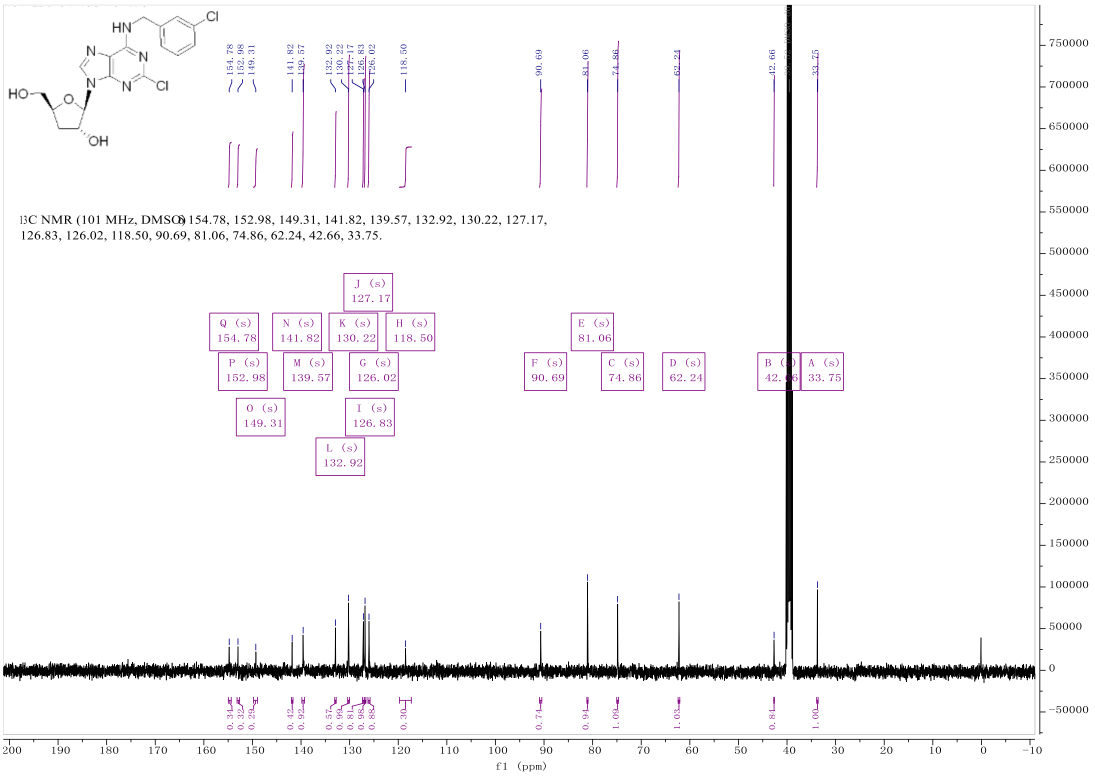

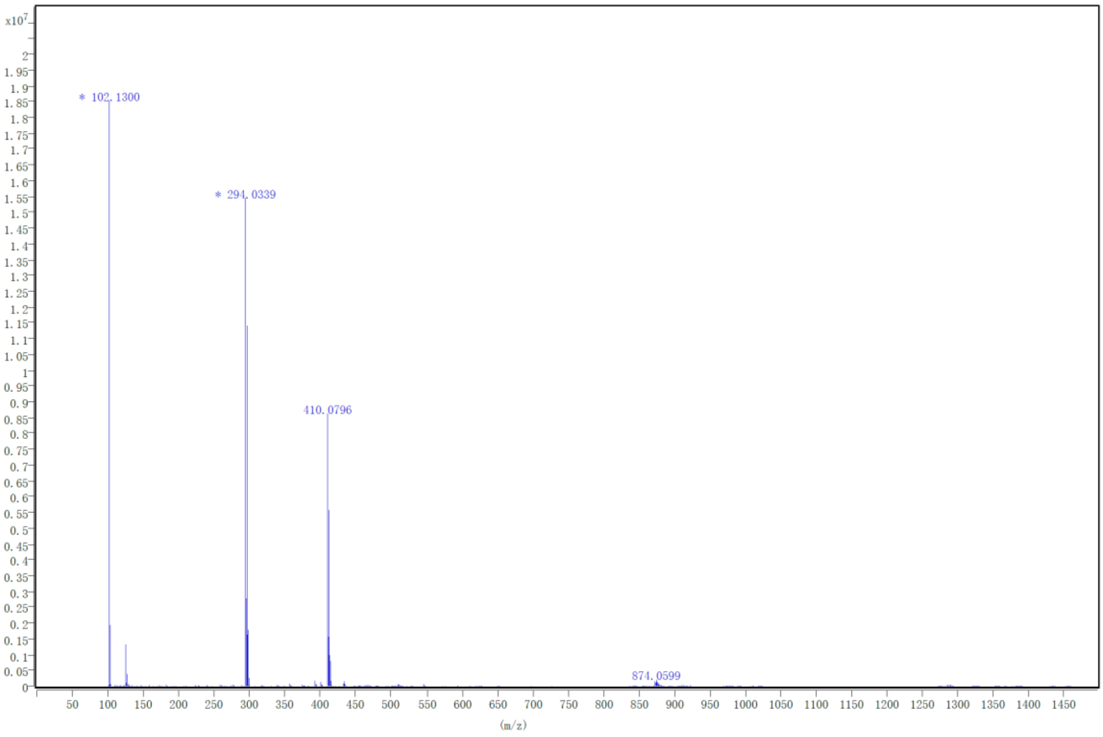

Figure S22. ^{1}**H NMR,** ^{13}**C NMR** and Mass spectra spectrum of compound **2a**

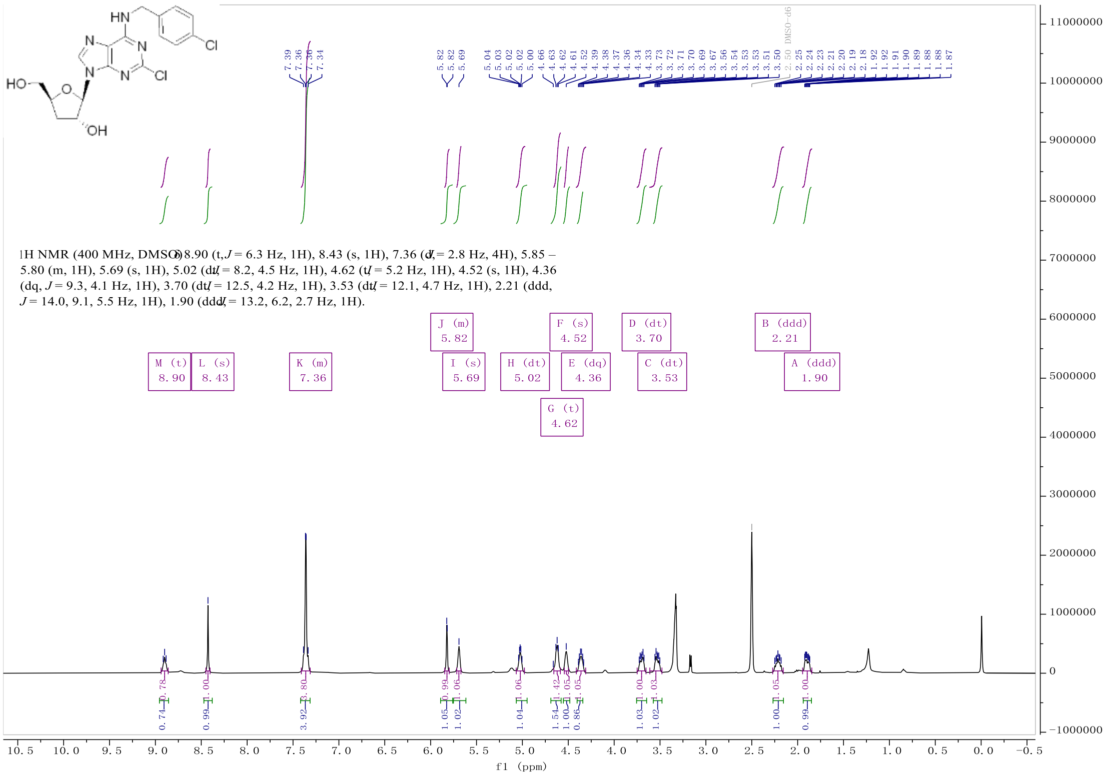

1H NMR (400 MHz, DMSO) δ 8.90 (t, J = 6.3 Hz, 1H), 8.43 (s, 1H), 7.36 (d, J = 2.8 Hz, 4H), 5.85 – 5.80 (m, 1H), 5.69 (s, 1H), 5.02 (dt, J = 8.2, 4.5 Hz, 1H), 4.62 (t, J = 5.2 Hz, 1H), 4.52 (s, 1H), 4.36 (dq, J = 9.3, 4.1 Hz, 1H), 3.70 (dt, J = 12.5, 4.2 Hz, 1H), 3.53 (dt, J = 12.1, 4.7 Hz, 1H), 2.21 (ddd, J = 14.0, 9.1, 5.5 Hz, 1H), 1.90 (ddd, J = 13.2, 6.2, 2.7 Hz, 1H).

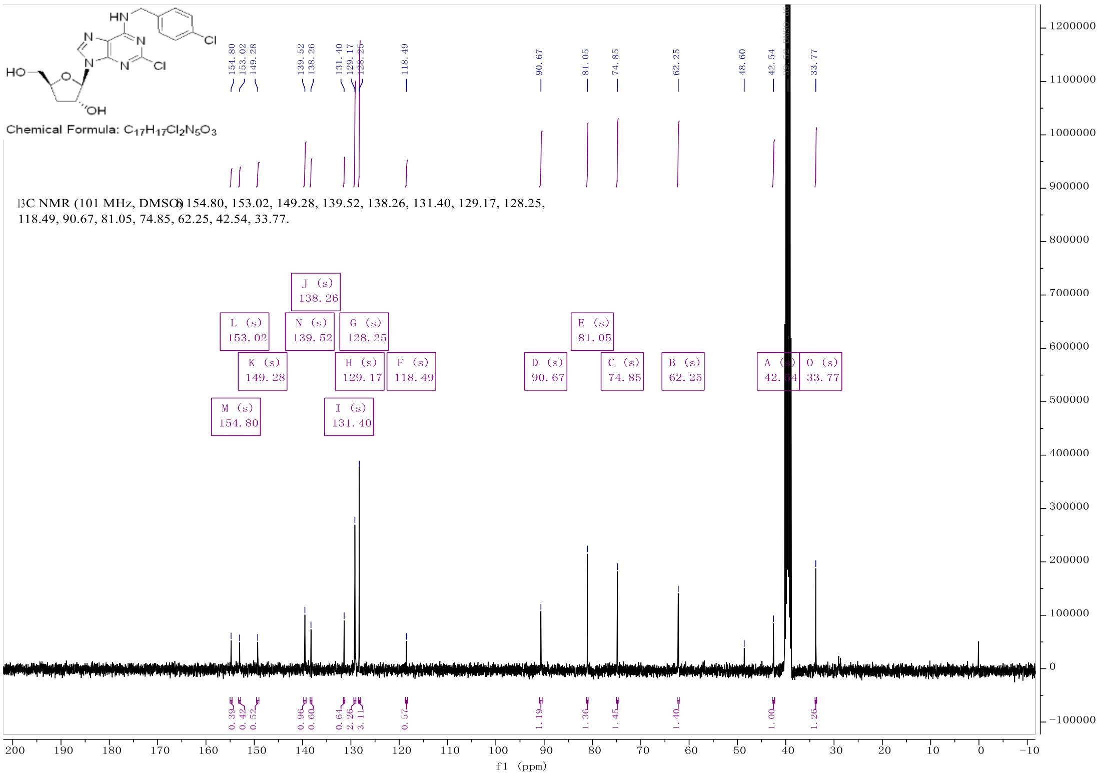

Chemical Formula: $C_{17}H_{17}Cl_2N_5O_3$

13C NMR (101 MHz, DMSO) δ 154.80, 153.02, 149.28, 139.52, 138.26, 131.40, 129.17, 128.25, 118.49, 90.67, 81.05, 74.85, 62.25, 42.54, 33.77.

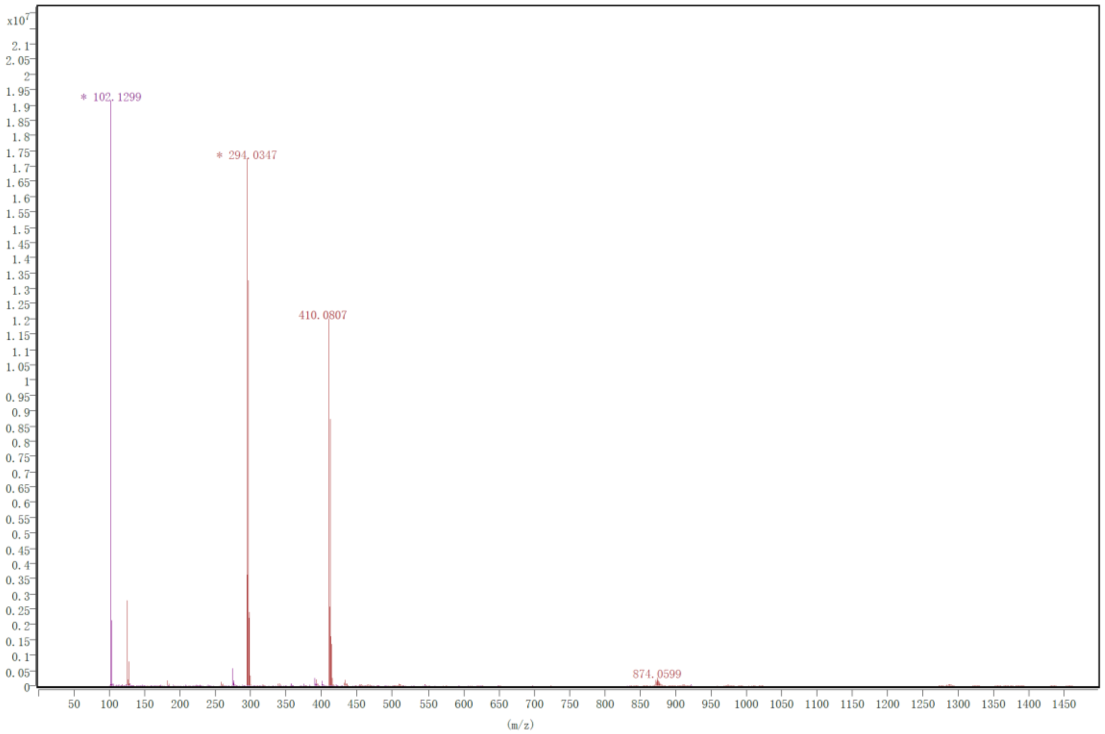

Figure S23. **^{1}H NMR，^{13}C NMR** and Mass spectra spectrum of compound **2b**

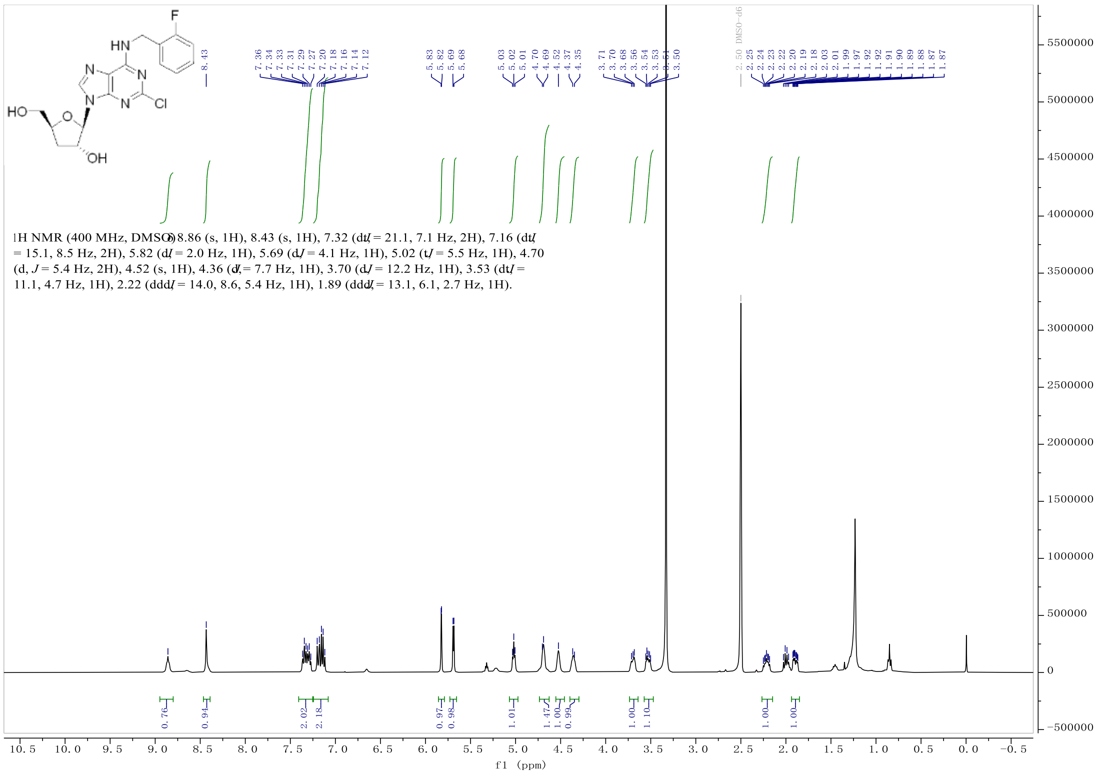

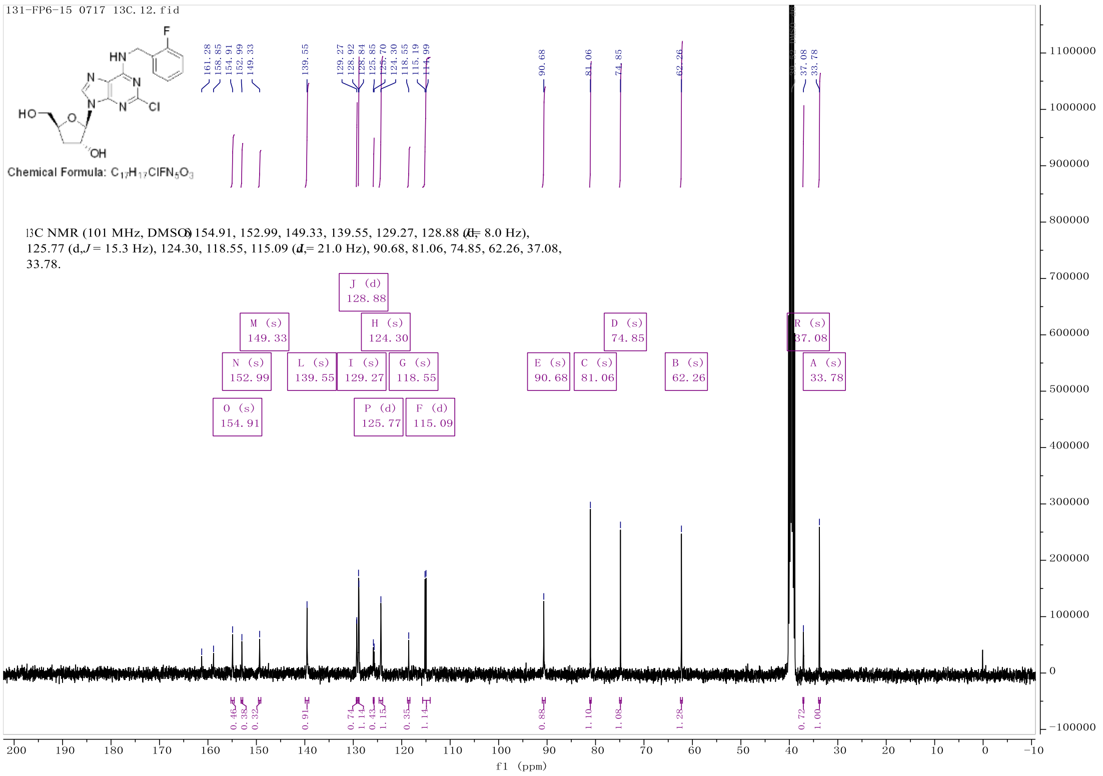

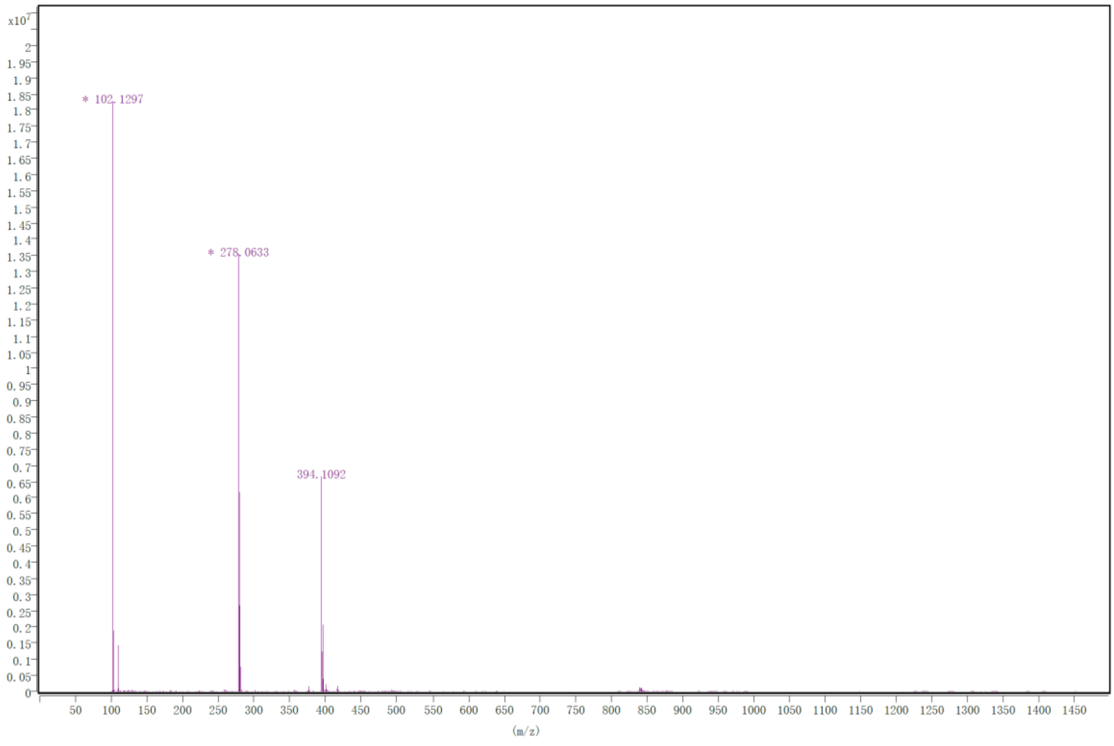

Figure S24. **^{1}H NMR,** **^{13}C NMR** and Mass spectra spectrum of compound **2c**

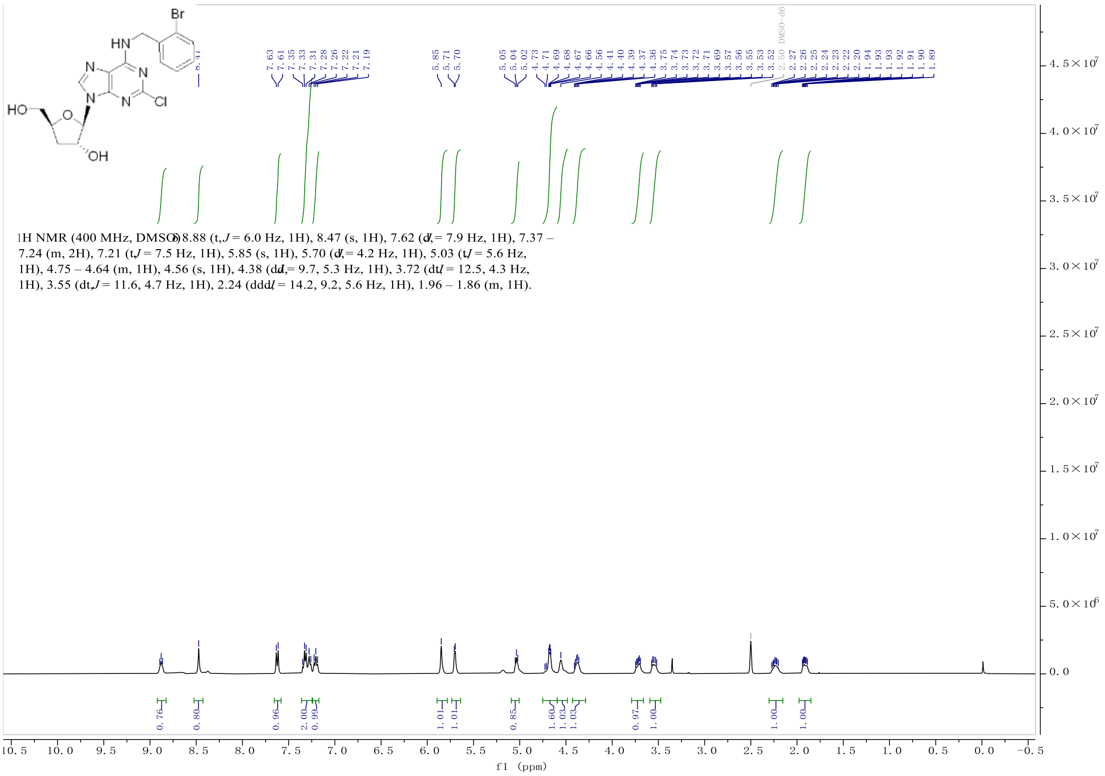
1H NMR (400 MHz, DMSO) δ 8.88 (t, J = 6.0 Hz, 1H), 8.47 (s, 1H), 7.62 (d, J = 7.9 Hz, 1H), 7.37 – 7.24 (m, 2H), 7.21 (t, J = 7.5 Hz, 1H), 5.85 (s, 1H), 5.70 (d, J = 4.2 Hz, 1H), 5.03 (t, J = 5.6 Hz, 1H), 4.75 – 4.64 (m, 1H), 4.56 (s, 1H), 4.38 (dd, J = 9.7, 5.3 Hz, 1H), 3.72 (dt, J = 12.5, 4.3 Hz, 1H), 3.55 (dt, J = 11.6, 4.7 Hz, 1H), 2.24 (ddd, J = 14.2, 9.2, 5.6 Hz, 1H), 1.96 – 1.86 (m, 1H).
f1 (ppm)

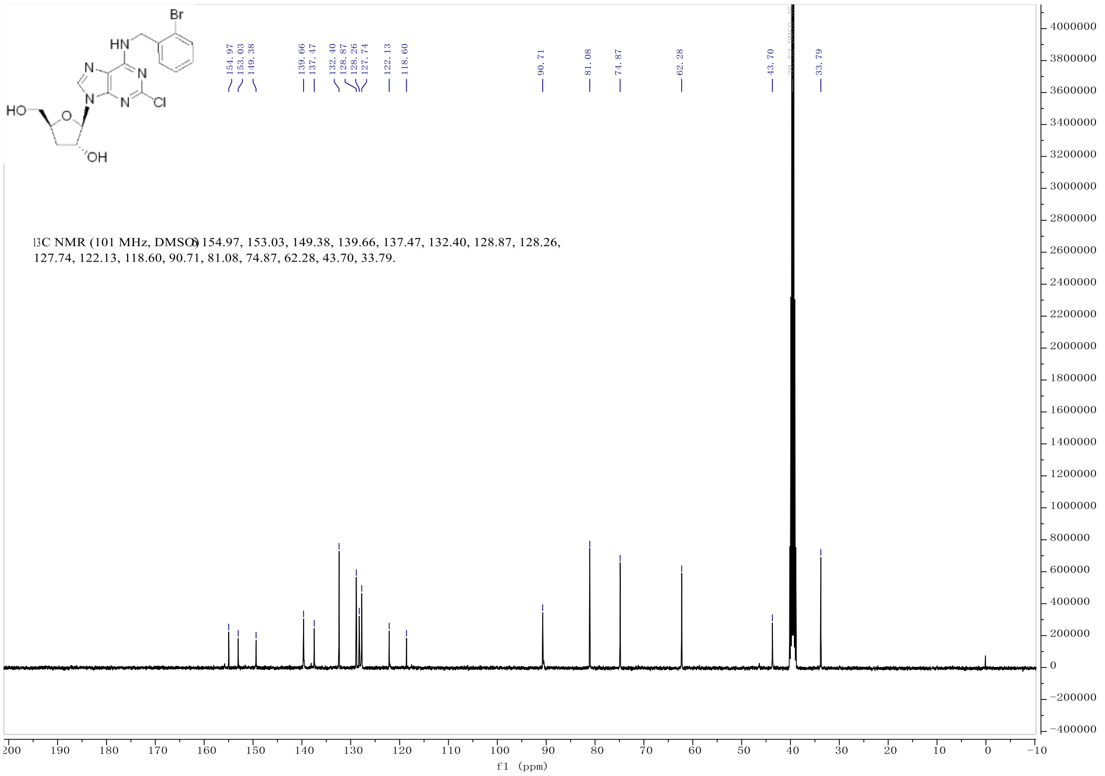
13C NMR (101 MHz, DMSO) δ 154.97, 153.03, 149.38, 139.66, 137.47, 132.40, 128.87, 128.26, 127.74, 122.13, 118.60, 90.71, 81.08, 74.87, 62.28, 43.70, 33.79.
f1 (ppm)

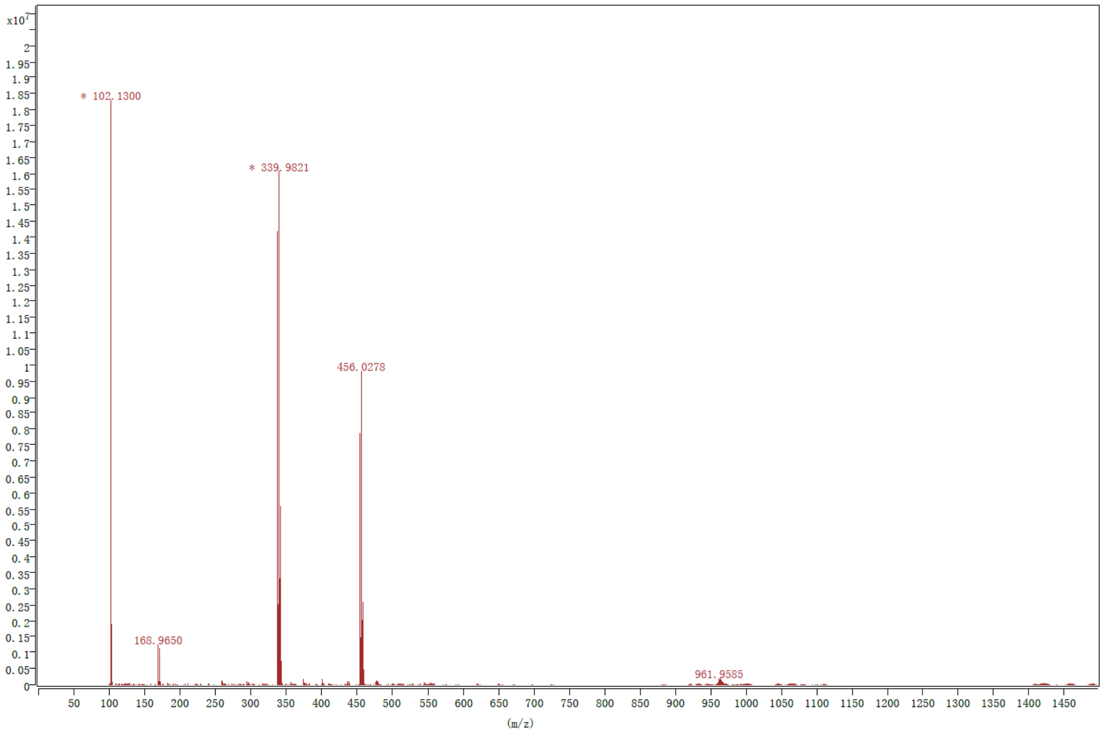

Figure S25. **^{1}H NMR**，**^{13}C NMR** and Mass spectra spectrum of compound **2d**

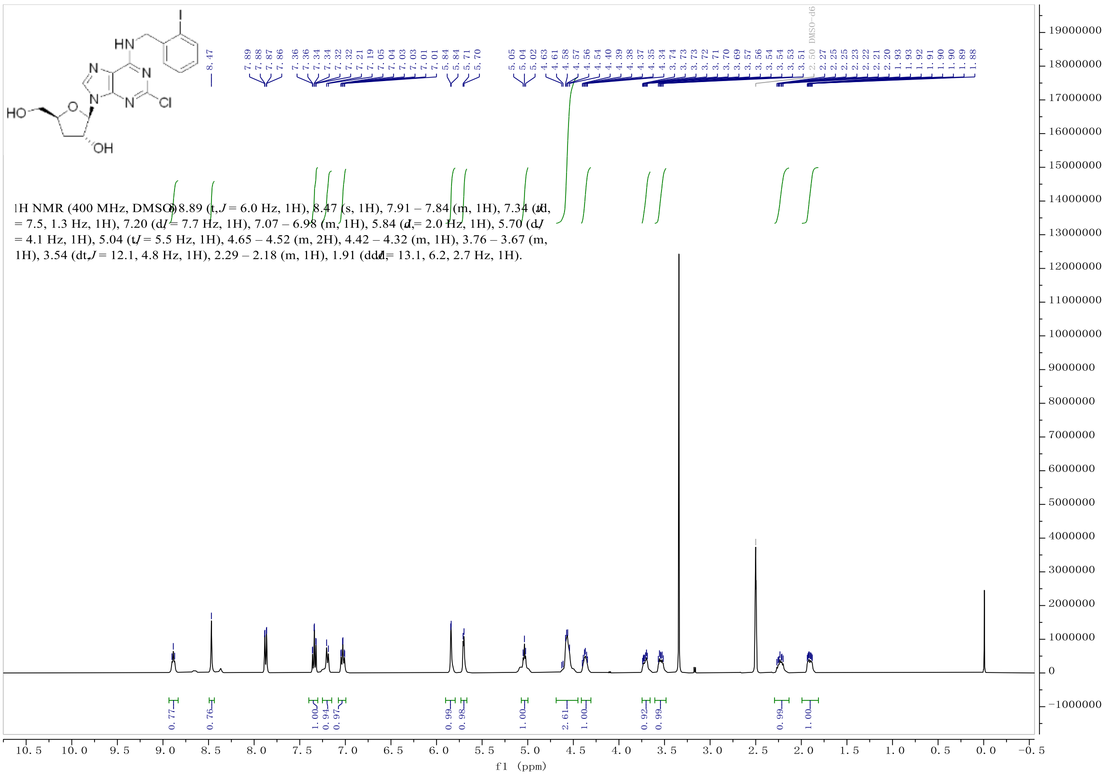

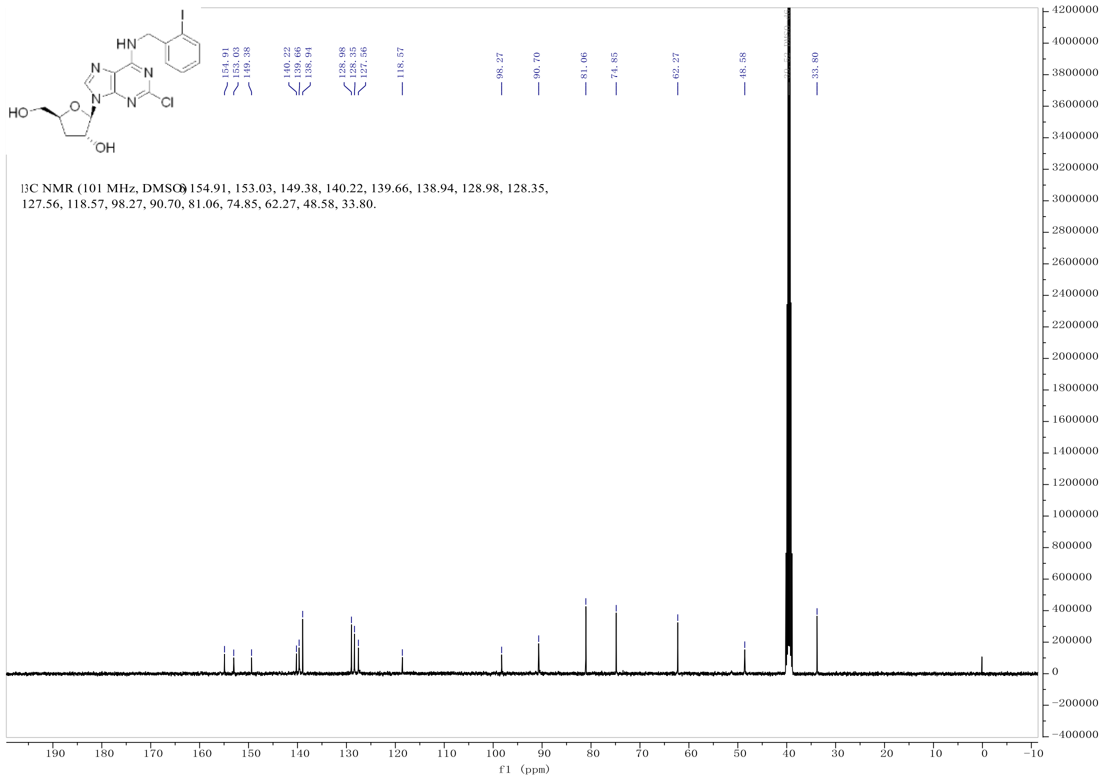

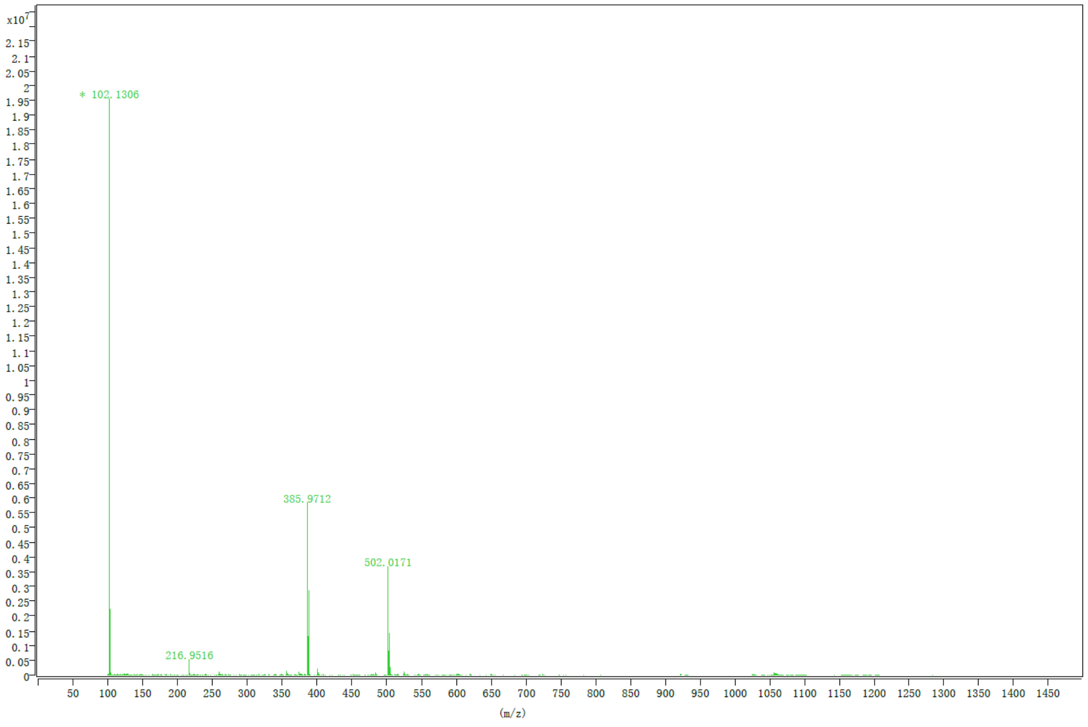

Figure S26. ^{1}**H NMR,** ^{13}**C NMR** and Mass spectra spectrum of compound **2e**

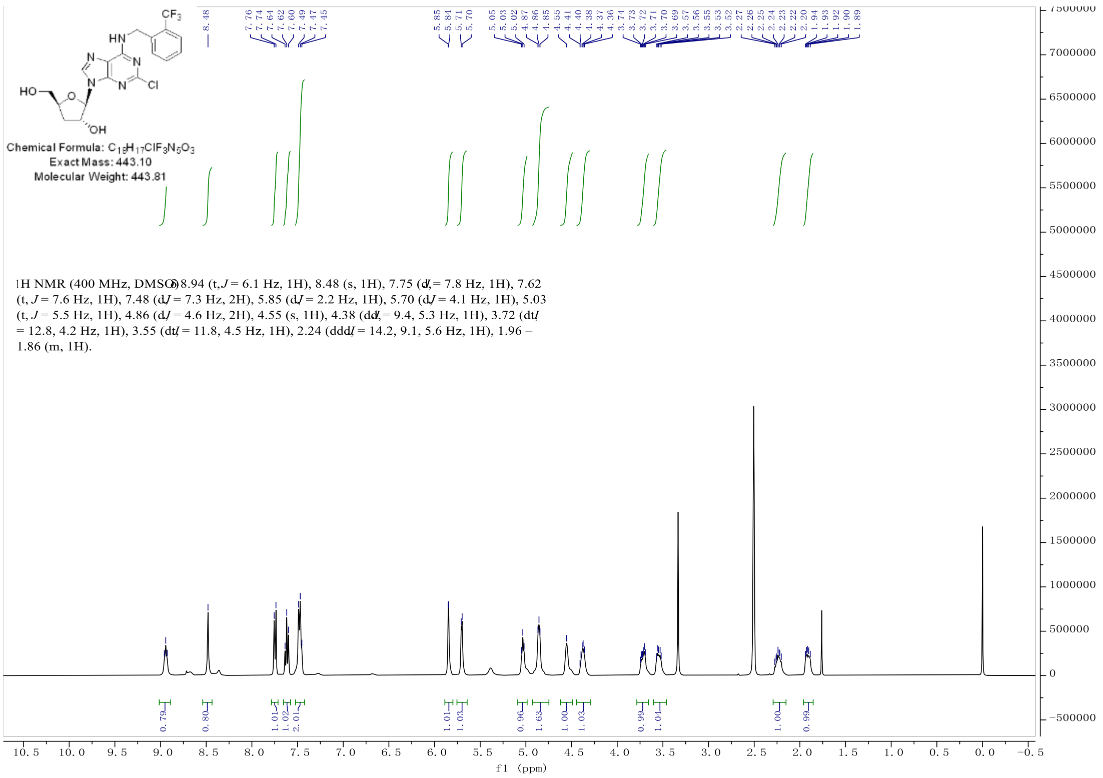
CF3
HN
N
N
N
N
Cl
HO
O
OH
Chemical Formula: C18H17ClF3N5O3
Exact Mass: 443.10
Molecular Weight: 443.81
1H NMR (400 MHz, DMSO) δ 8.94 (t, J = 6.1 Hz, 1H), 8.48 (s, 1H), 7.75 (d, J = 7.8 Hz, 1H), 7.62 (t, J = 7.6 Hz, 1H), 7.48 (d, J = 7.3 Hz, 2H), 5.85 (d, J = 2.2 Hz, 1H), 5.70 (d, J = 4.1 Hz, 1H), 5.03 (t, J = 5.5 Hz, 1H), 4.86 (d, J = 4.6 Hz, 2H), 4.55 (s, 1H), 4.38 (dd, J = 9.4, 5.3 Hz, 1H), 3.72 (dt, J = 12.8, 4.2 Hz, 1H), 3.55 (dt, J = 11.8, 4.5 Hz, 1H), 2.24 (ddd, J = 14.2, 9.1, 5.6 Hz, 1H), 1.96 – 1.86 (m, 1H).
f1 (ppm)

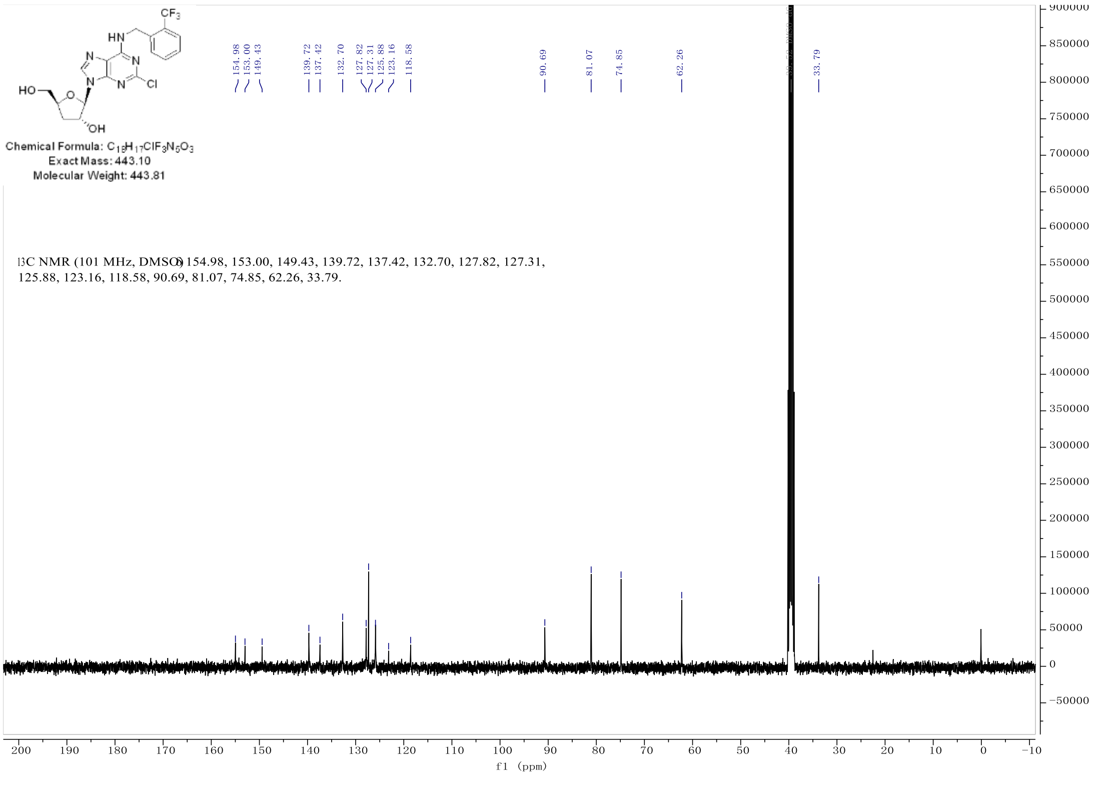
CF3
HN
N
N
N
N
Cl
HO
O
OH
Chemical Formula: C18H17ClF3N5O3
Exact Mass: 443.10
Molecular Weight: 443.81
13C NMR (101 MHz, DMSO) δ 154.98, 153.00, 149.43, 139.72, 137.42, 132.70, 127.82, 127.31, 125.88, 123.16, 118.58, 90.69, 81.07, 74.85, 62.26, 33.79.
f1 (ppm)

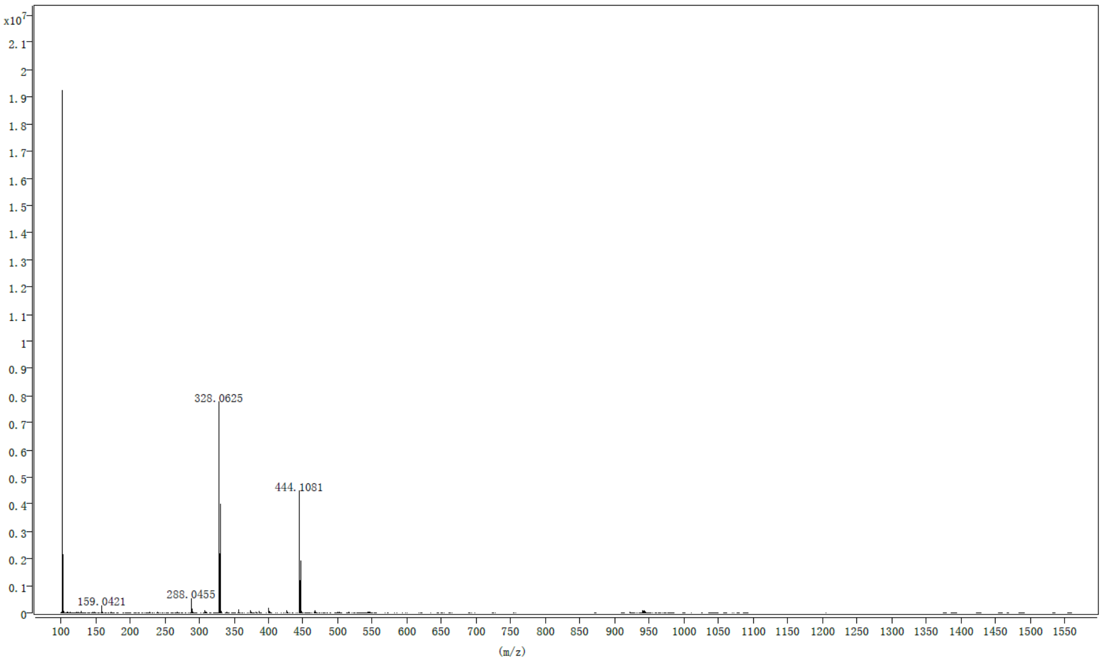

Figure S27. ^{1}**H NMR,** ^{13}**C NMR** and Mass spectra spectrum of compound **2f**

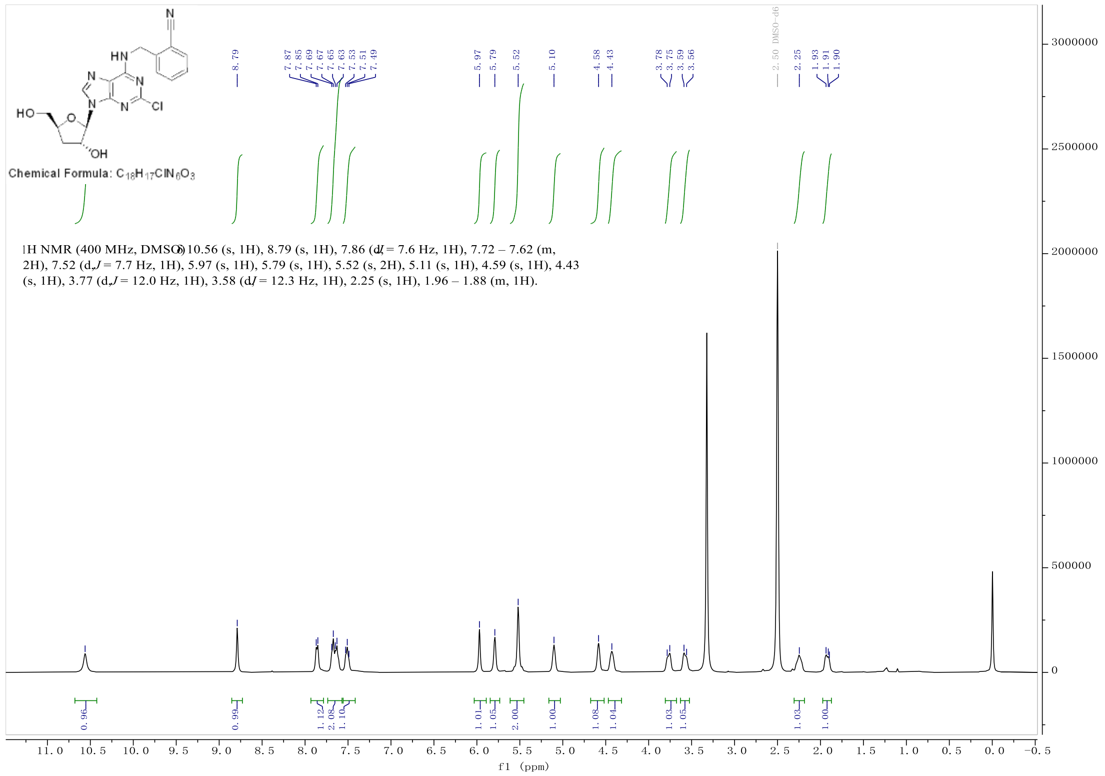

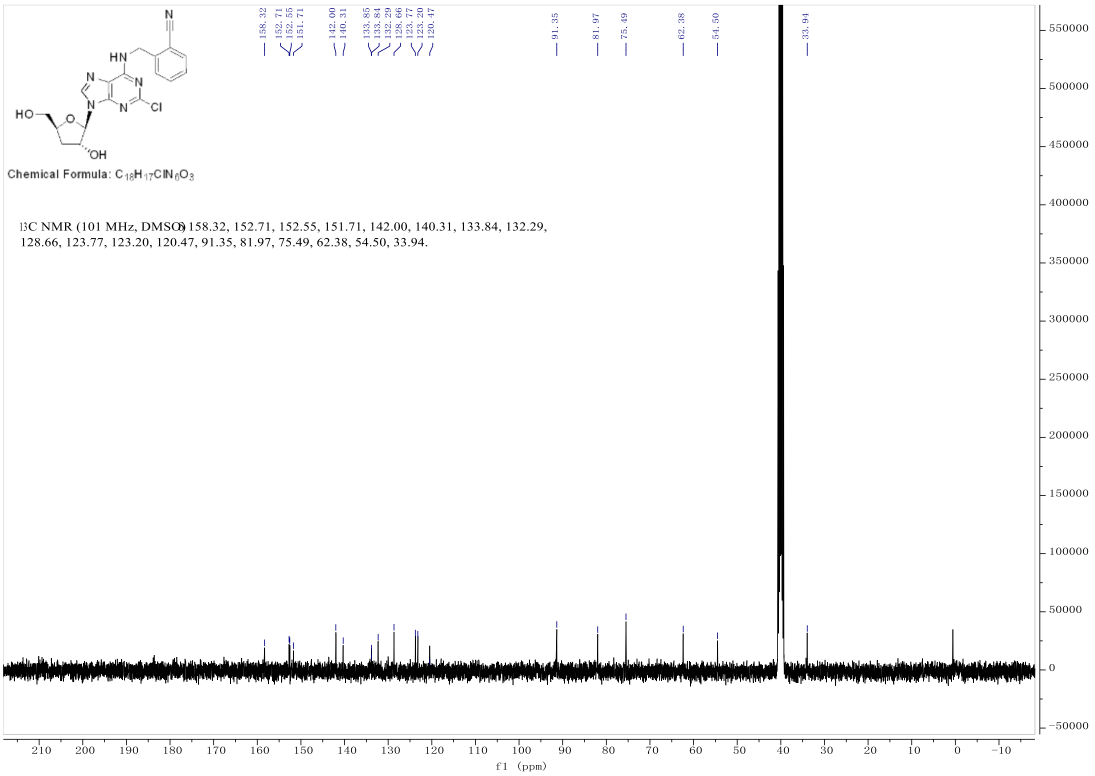

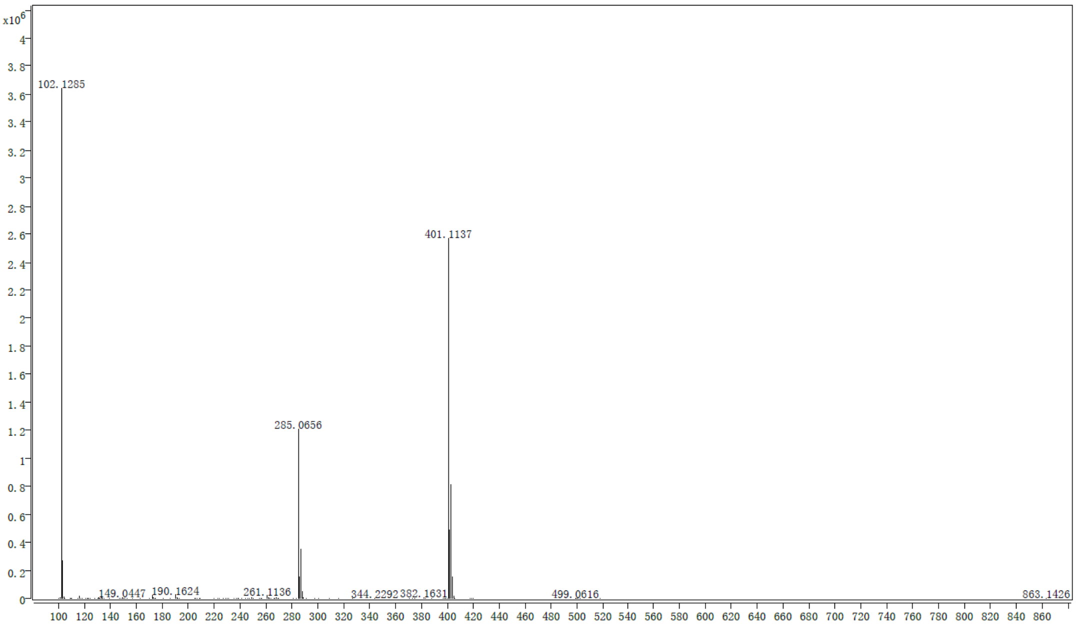

Figure S28. ^{1}**H NMR,** ^{13}**C NMR** and Mass spectra spectrum of compound **2g**

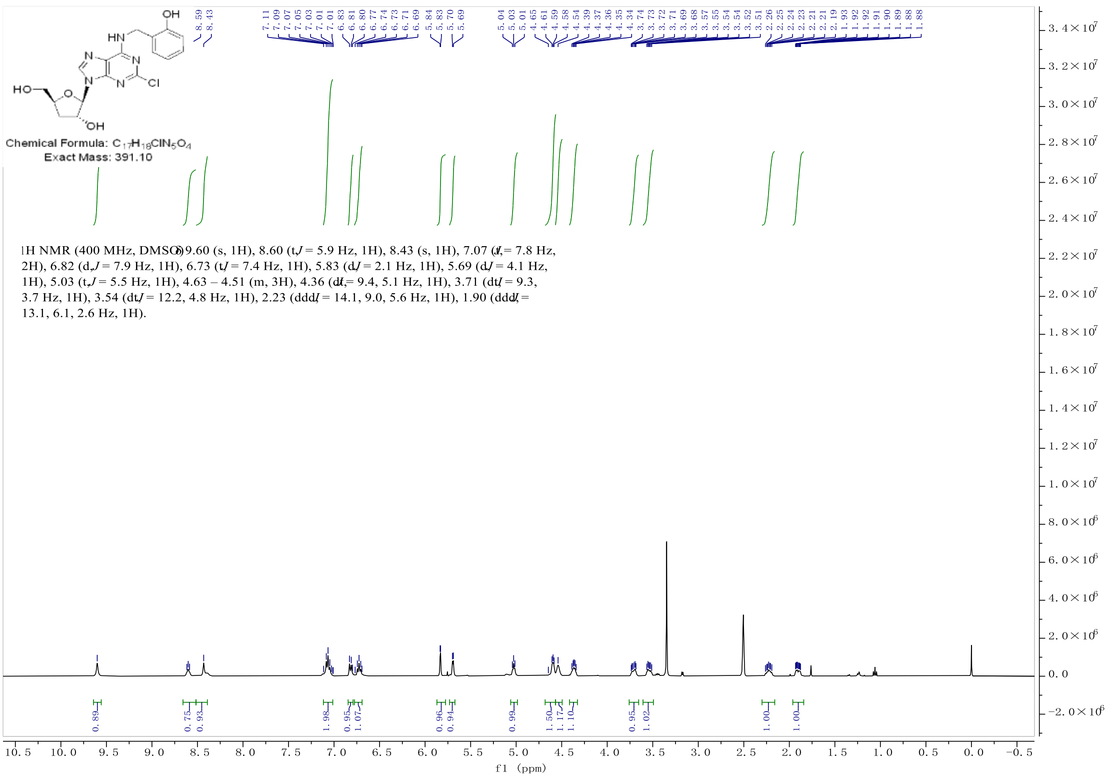
Chemical Formula: $C_{17}H_{18}ClN_5O_4$
Exact Mass: 391.10
1H NMR (400 MHz, DMSO) δ 9.60 (s, 1H), 8.60 (t, J = 5.9 Hz, 1H), 8.43 (s, 1H), 7.07 (d, J = 7.8 Hz, 2H), 6.82 (d, J = 7.9 Hz, 1H), 6.73 (t, J = 7.4 Hz, 1H), 5.83 (d, J = 2.1 Hz, 1H), 5.69 (d, J = 4.1 Hz, 1H), 5.03 (t, J = 5.5 Hz, 1H), 4.63 – 4.51 (m, 3H), 4.36 (dt, J = 9.4, 5.1 Hz, 1H), 3.71 (dt, J = 9.3, 3.7 Hz, 1H), 3.54 (dt, J = 12.2, 4.8 Hz, 1H), 2.23 (ddd, J = 14.1, 9.0, 5.6 Hz, 1H), 1.90 (ddd, J = 13.1, 6.1, 2.6 Hz, 1H).
f1 (ppm)

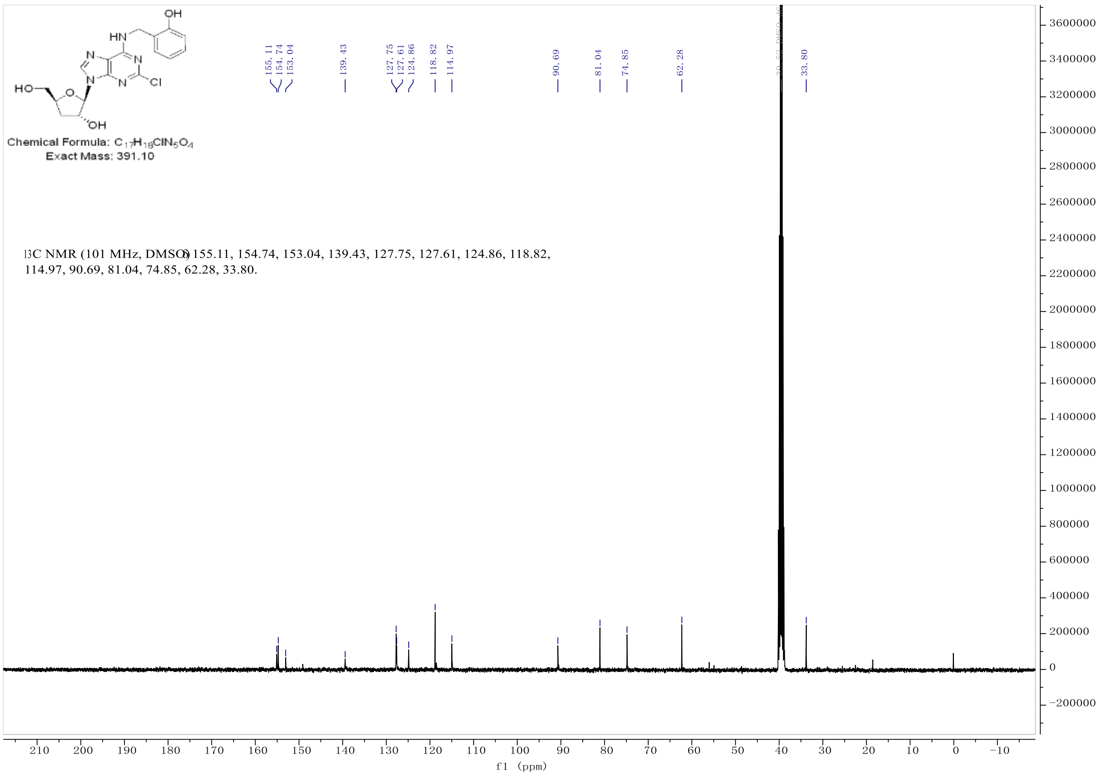
Chemical Formula: $C_{17}H_{18}ClN_5O_4$
Exact Mass: 391.10
13C NMR (101 MHz, DMSO) δ 155.11, 154.74, 153.04, 139.43, 127.75, 127.61, 124.86, 118.82, 114.97, 90.69, 81.04, 74.85, 62.28, 33.80.
f1 (ppm)

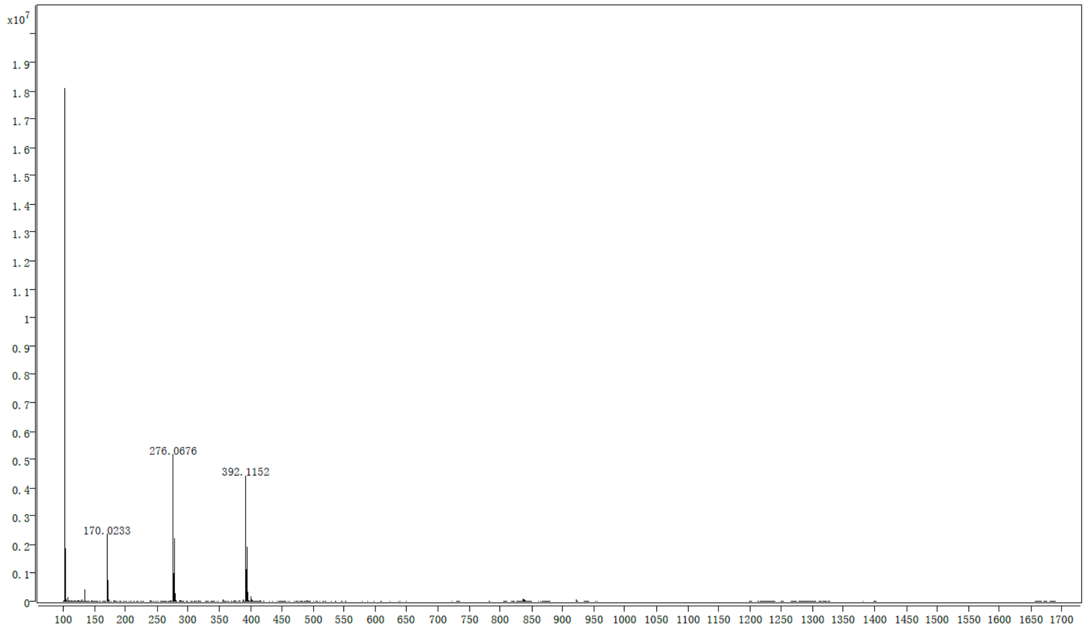

Figure S29. [1]**H NMR,** [13]**C NMR** and Mass spectra spectrum of compound **2h**

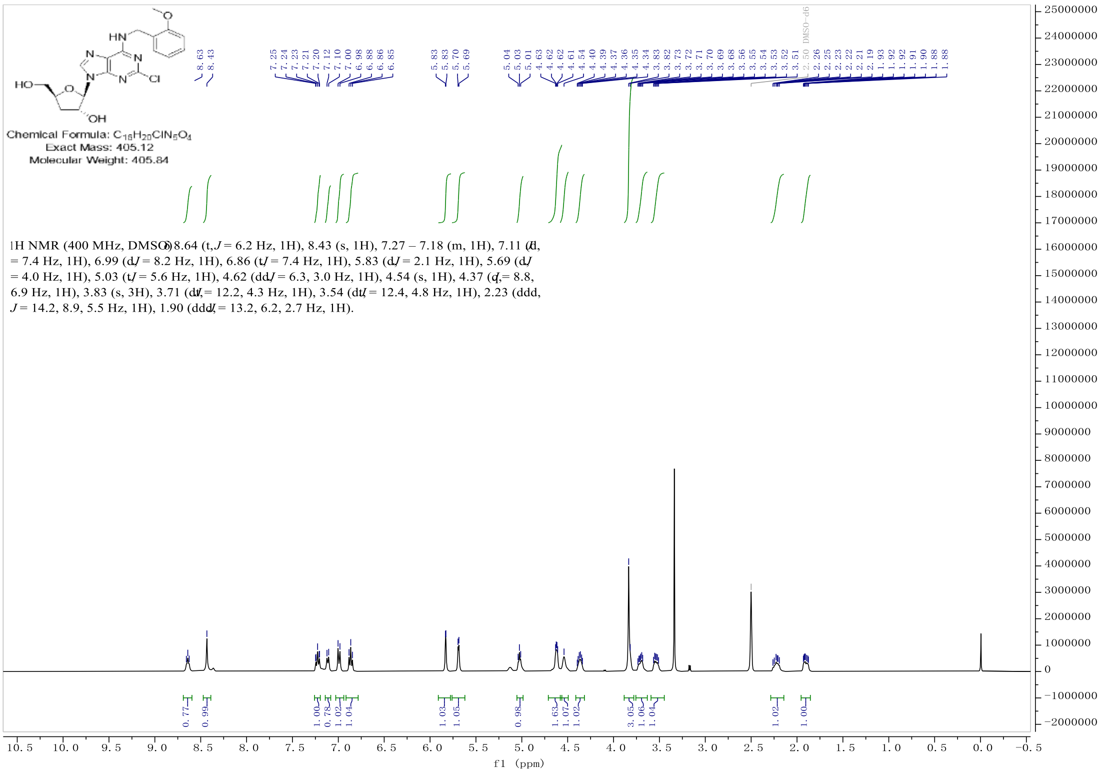

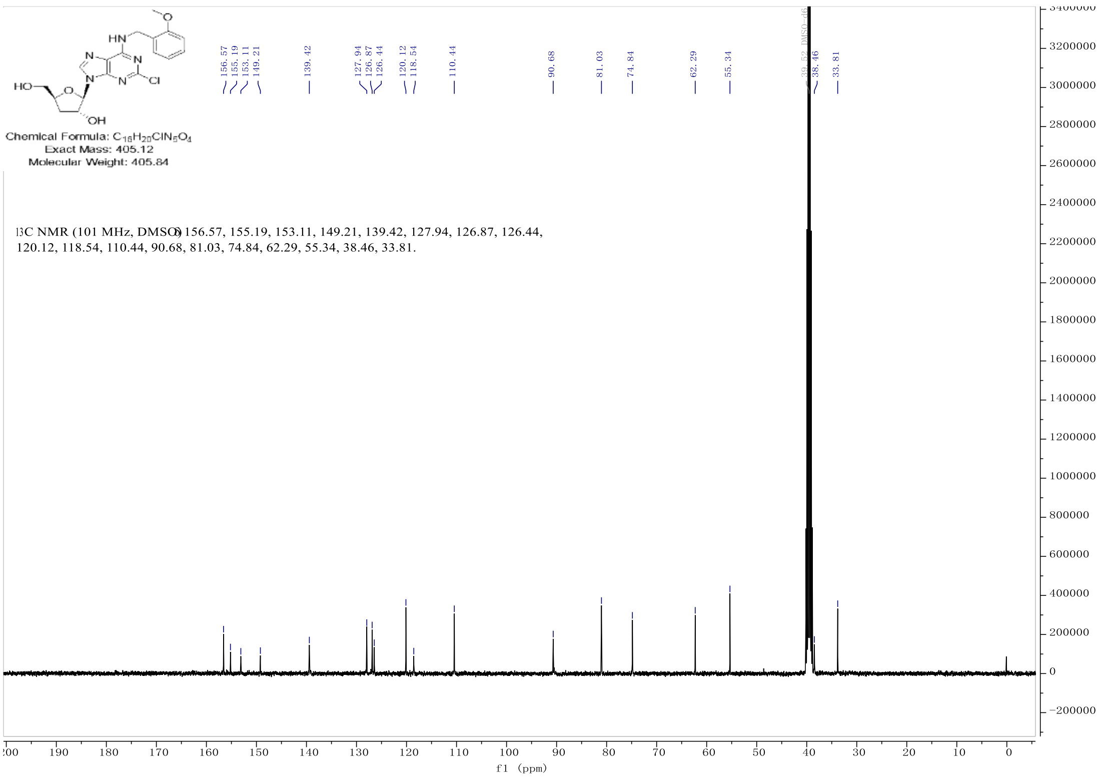

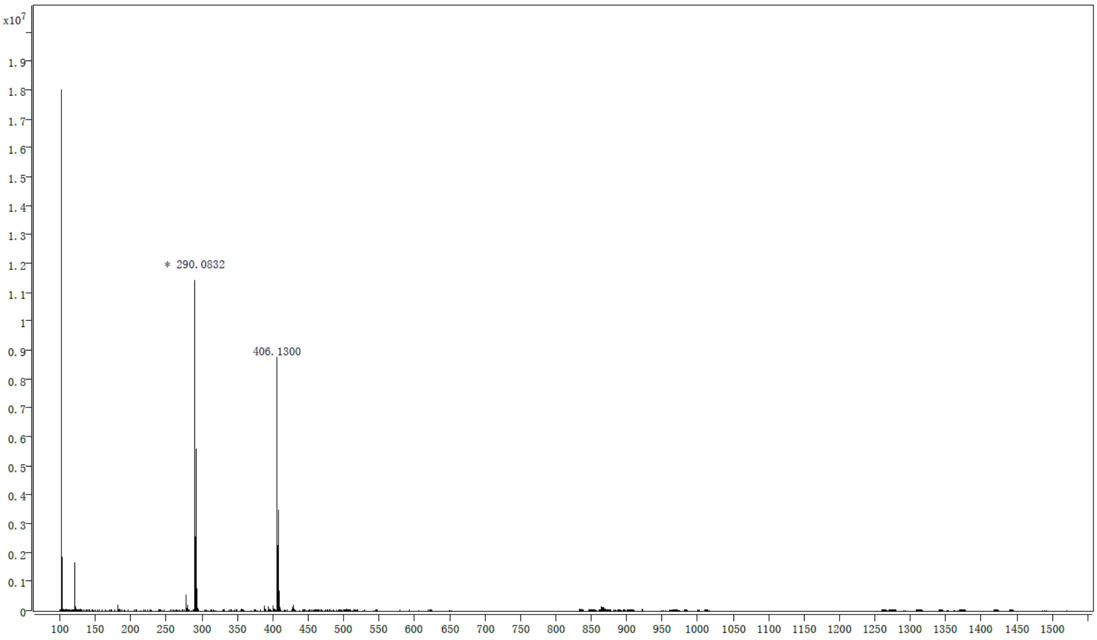

Figure S30. ^{1}**H NMR,** ^{13}**C NMR** and Mass spectra spectrum of compound **2i**

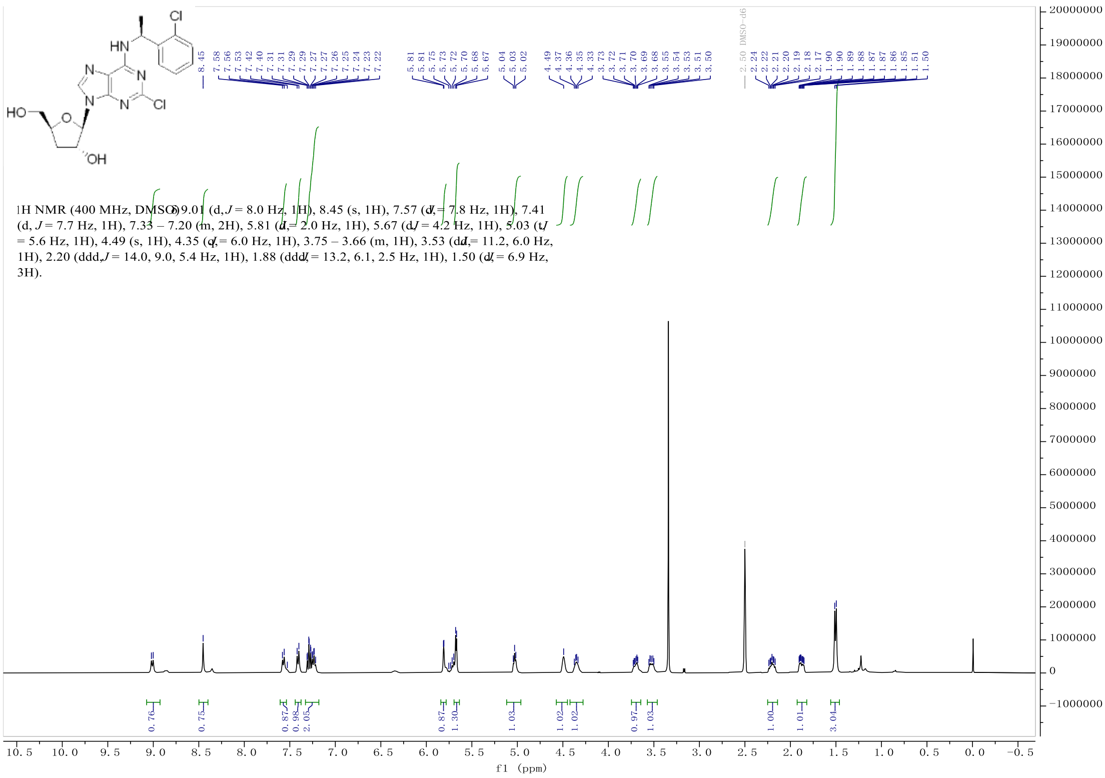
Cl
HN
N
N
N
N
HO
O
Cl
OH
1H NMR (400 MHz, DMSO) δ 9.01 (d, J = 8.0 Hz, 1H), 8.45 (s, 1H), 7.57 (d, J = 7.8 Hz, 1H), 7.41 (d, J = 7.7 Hz, 1H), 7.33 – 7.20 (m, 2H), 5.81 (d, J = 2.0 Hz, 1H), 5.67 (d, J = 4.2 Hz, 1H), 5.03 (t, J = 5.6 Hz, 1H), 4.49 (s, 1H), 4.35 (q, J = 6.0 Hz, 1H), 3.75 – 3.66 (m, 1H), 3.53 (dd, J = 11.2, 6.0 Hz, 1H), 2.20 (ddd, J = 14.0, 9.0, 5.4 Hz, 1H), 1.88 (ddd, J = 13.2, 6.1, 2.5 Hz, 1H), 1.50 (d, J = 6.9 Hz, 3H).
2.50 DMSO-d6
f1 (ppm)

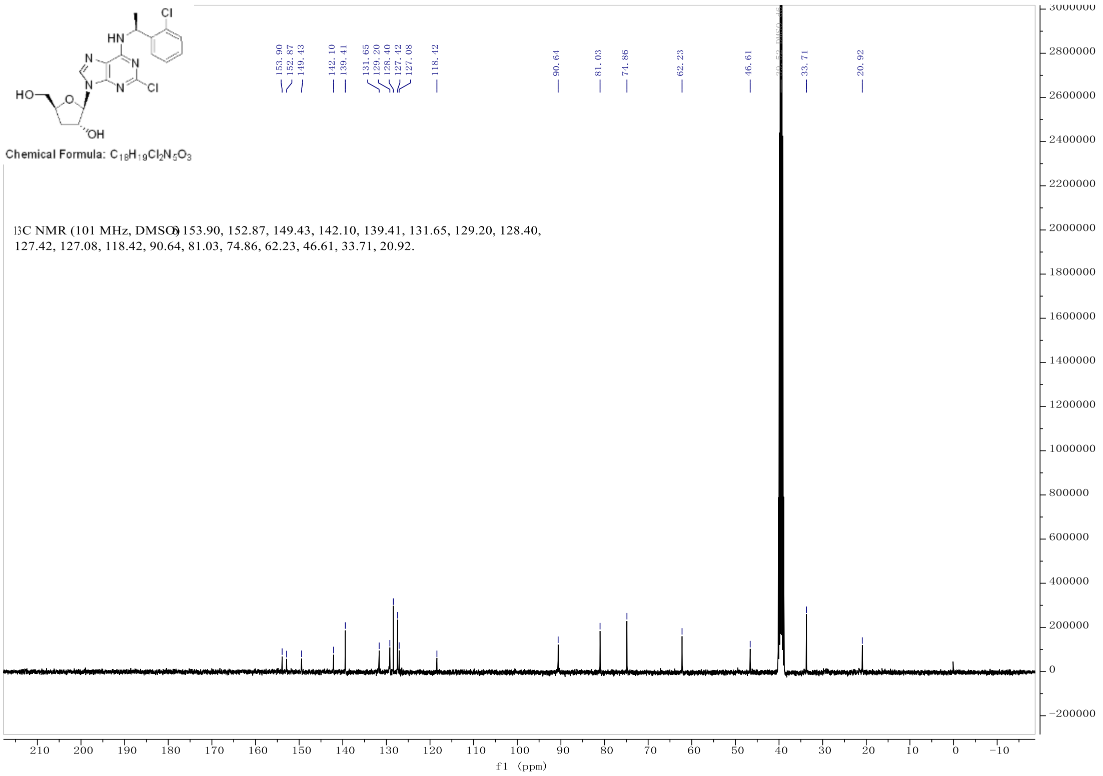
Cl
HN
N
N
N
N
HO
O
Cl
OH
Chemical Formula: $C_{18}H_{19}Cl_2N_5O_3$
13C NMR (101 MHz, DMSO) δ 153.90, 152.87, 149.43, 142.10, 139.41, 131.65, 129.20, 128.40, 127.42, 127.08, 118.42, 90.64, 81.03, 74.86, 62.23, 46.61, 33.71, 20.92.
f1 (ppm)

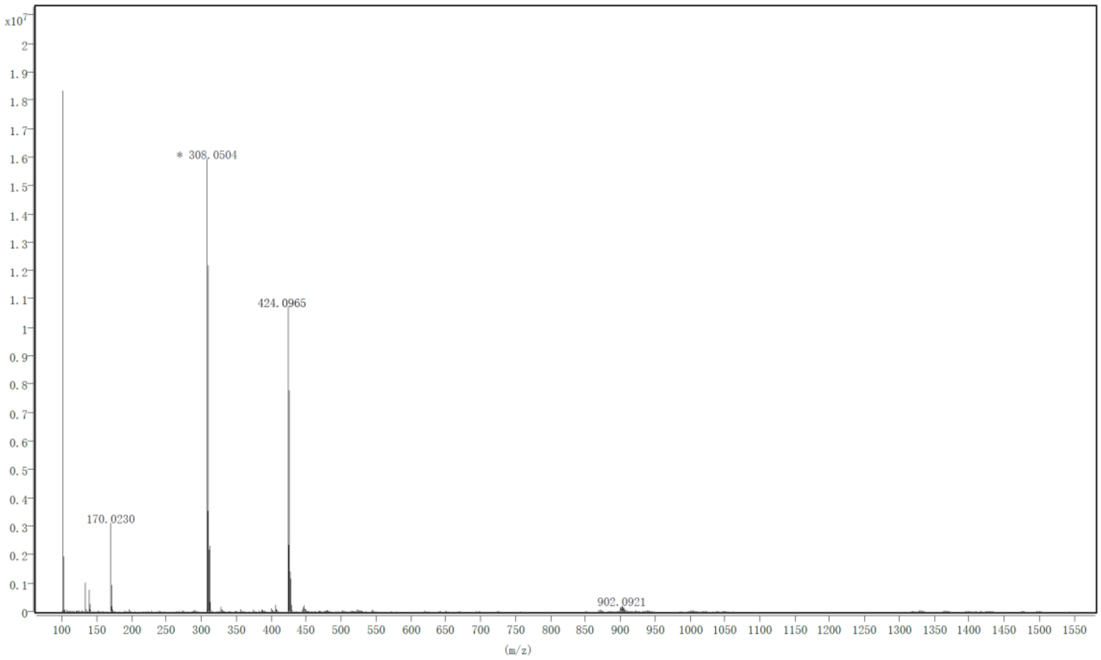

Figure S31. ^{1}**H NMR,** ^{13}**C NMR** and Mass spectra spectrum of compound **2j**

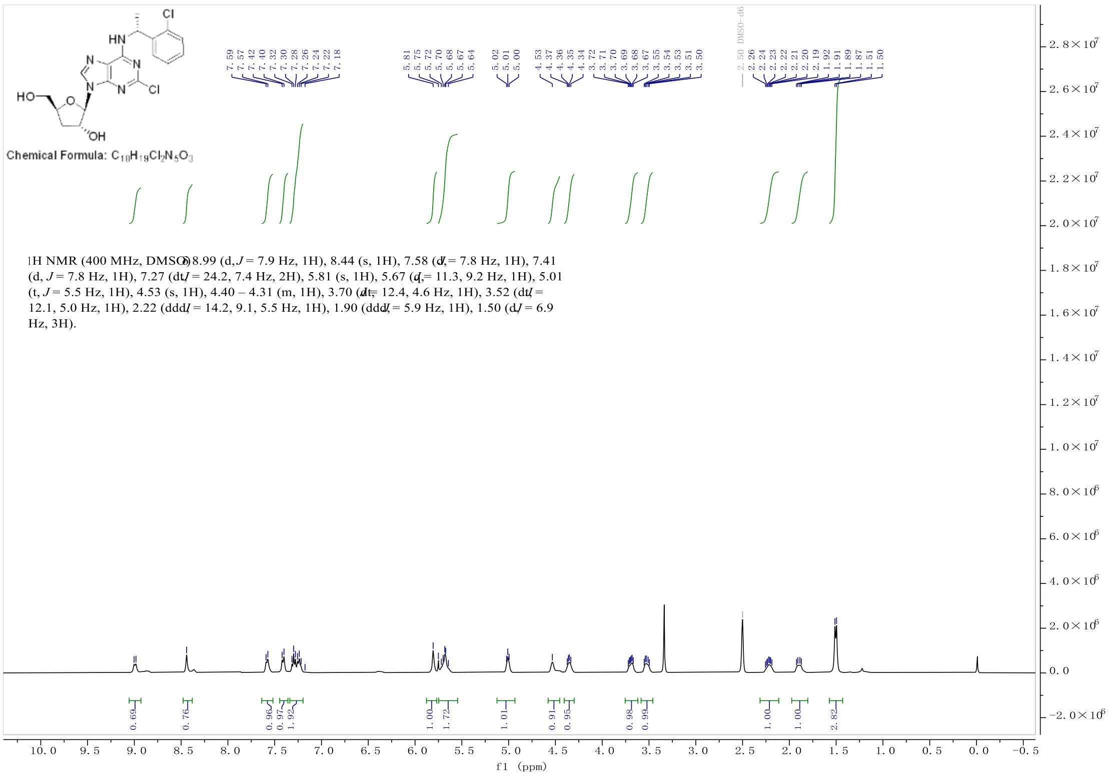

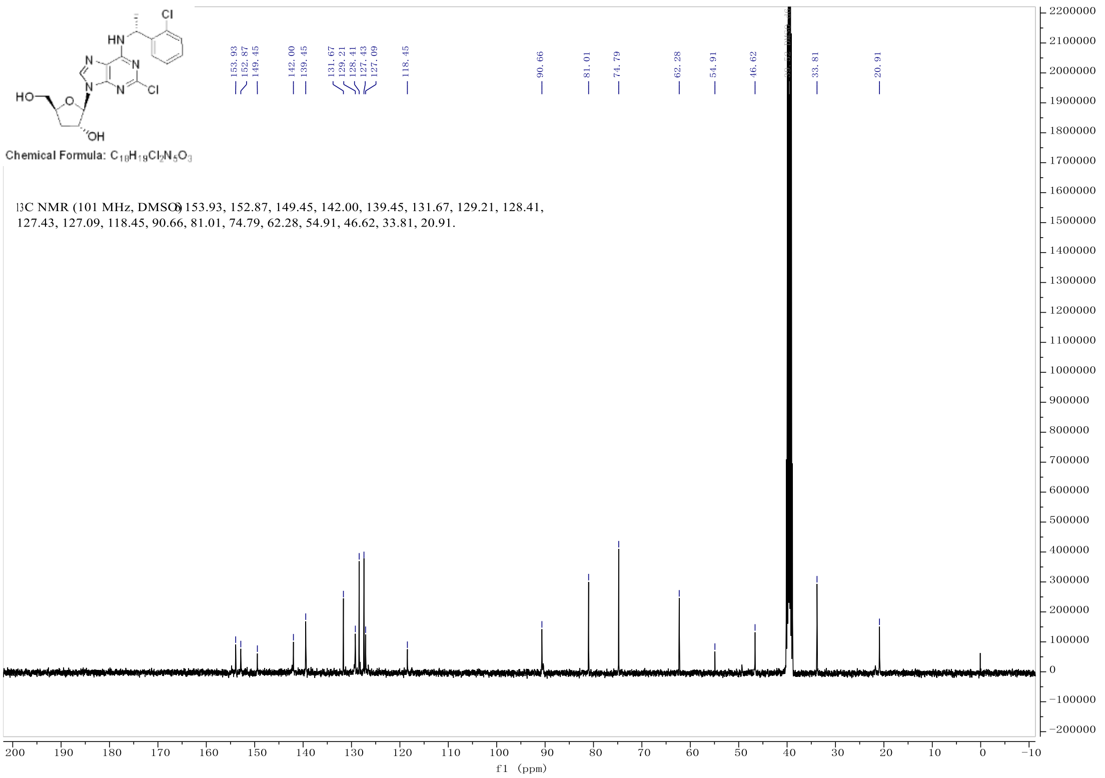

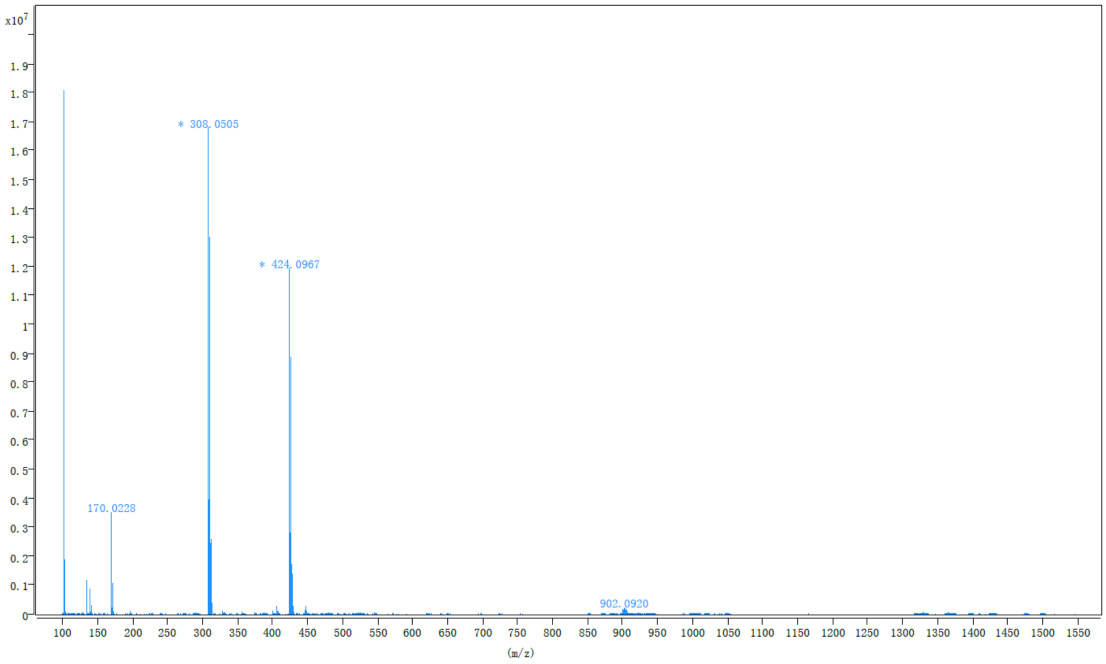

Figure S32. ^{1}**H NMR,** ^{13}**C NMR** and Mass spectra spectrum of compound **2k**

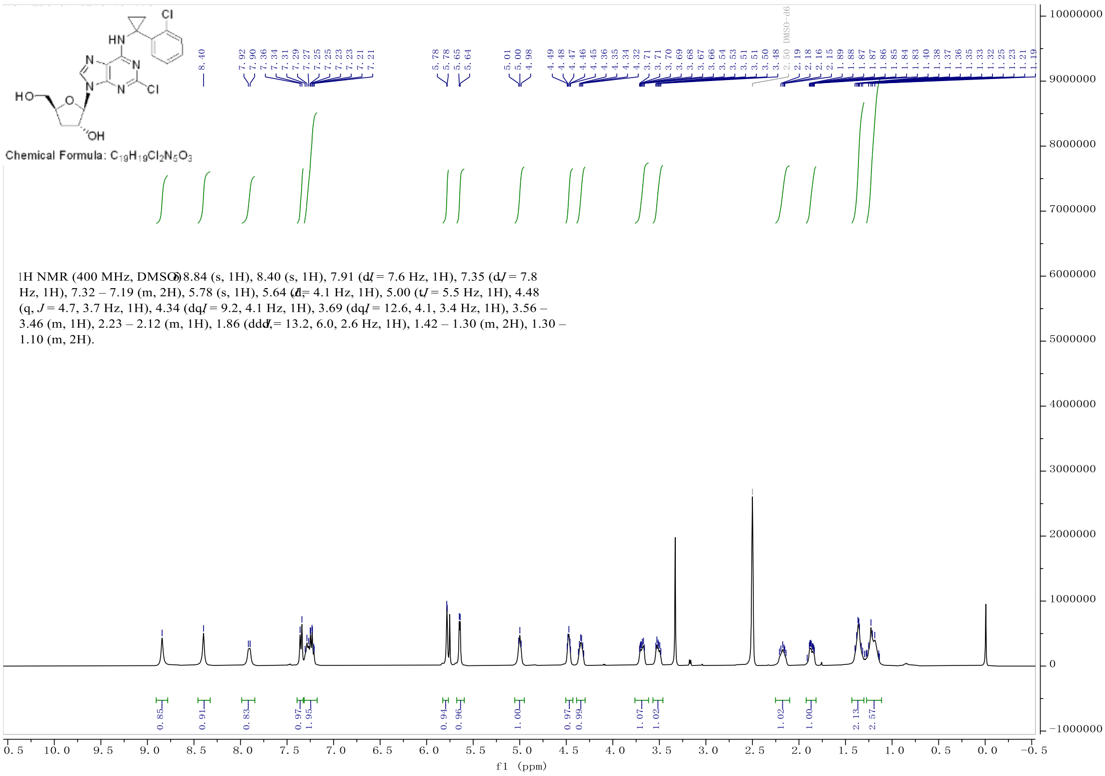
Chemical Formula: C19H19Cl2N5O3
1H NMR (400 MHz, DMSO) δ 8.84 (s, 1H), 8.40 (s, 1H), 7.91 (d, J = 7.6 Hz, 1H), 7.35 (d, J = 7.8 Hz, 1H), 7.32 – 7.19 (m, 2H), 5.78 (s, 1H), 5.64 (d, J = 4.1 Hz, 1H), 5.00 (t, J = 5.5 Hz, 1H), 4.48 (q, J = 4.7, 3.7 Hz, 1H), 4.34 (dq, J = 9.2, 4.1 Hz, 1H), 3.69 (dq, J = 12.6, 4.1, 3.4 Hz, 1H), 3.56 – 3.46 (m, 1H), 2.23 – 2.12 (m, 1H), 1.86 (ddd, J = 13.2, 6.0, 2.6 Hz, 1H), 1.42 – 1.30 (m, 2H), 1.30 – 1.10 (m, 2H).
f1 (ppm)

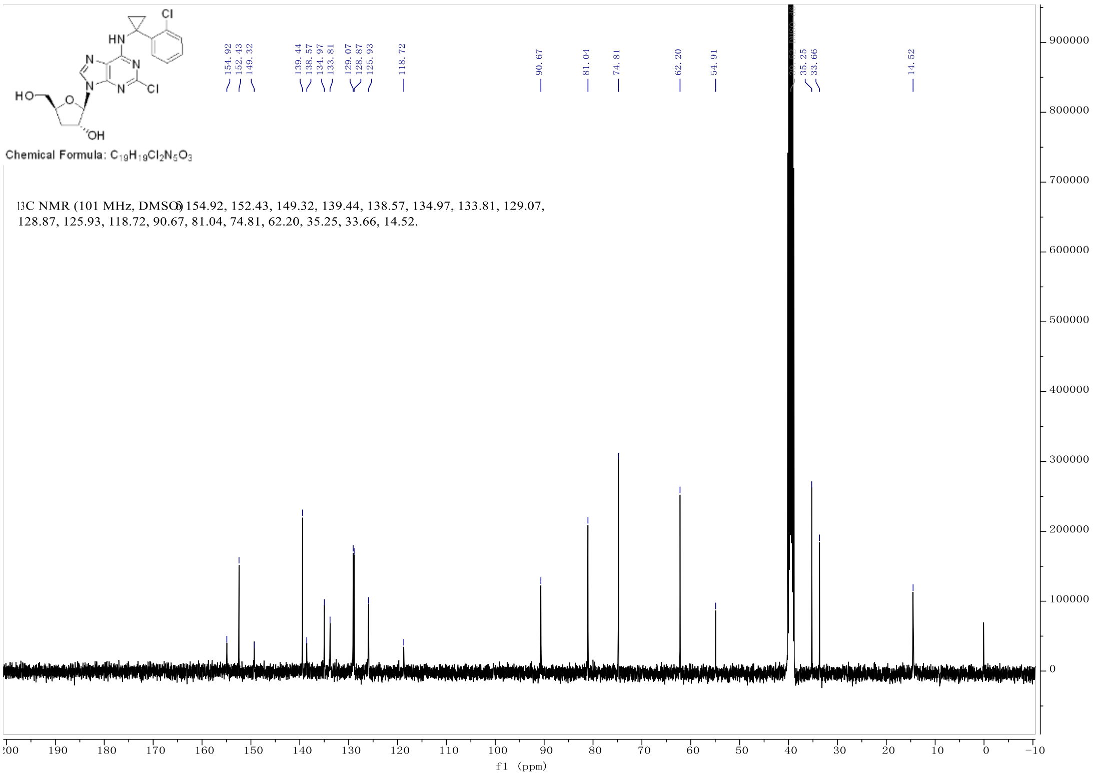
Chemical Formula: C19H19Cl2N5O3
13C NMR (101 MHz, DMSO) δ 154.92, 152.43, 149.32, 139.44, 138.57, 134.97, 133.81, 129.07, 128.87, 125.93, 118.72, 90.67, 81.04, 74.81, 62.20, 35.25, 33.66, 14.52.
f1 (ppm)

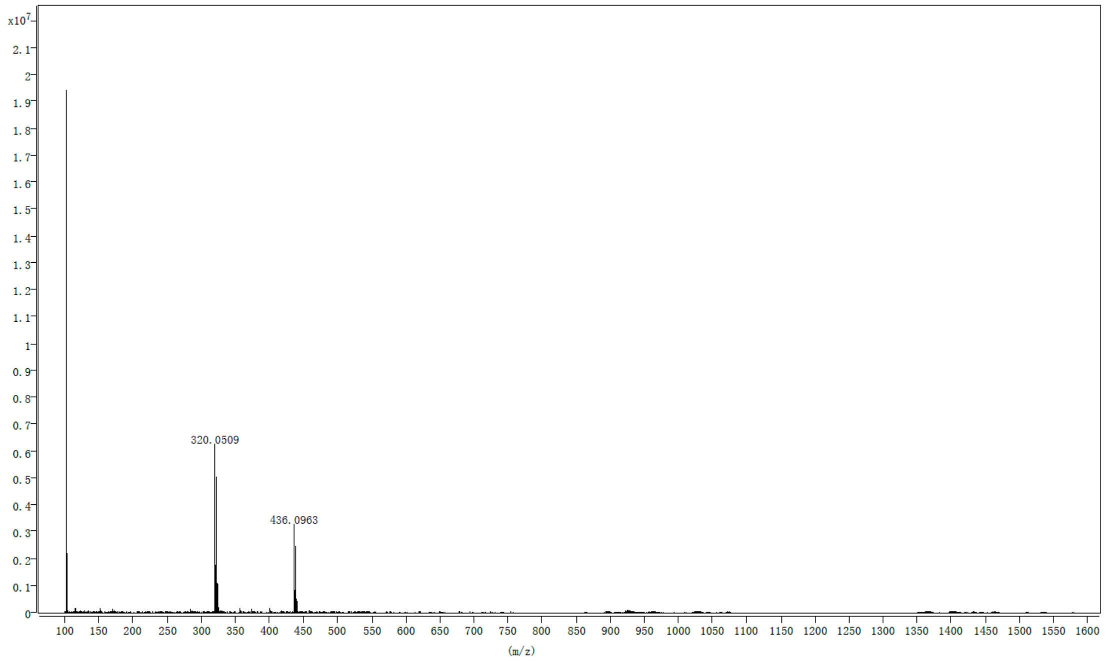

Figure S33. ^{1}H NMR, ^{13}C NMR and Mass spectra spectrum of compound **2l**

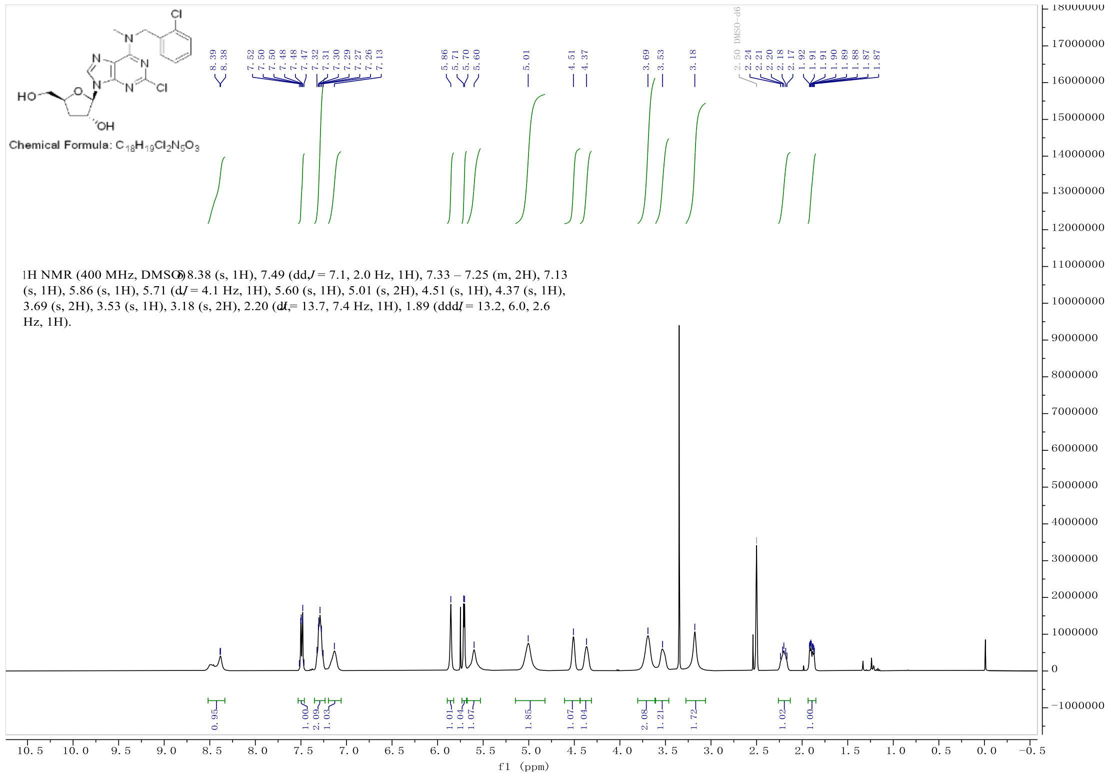

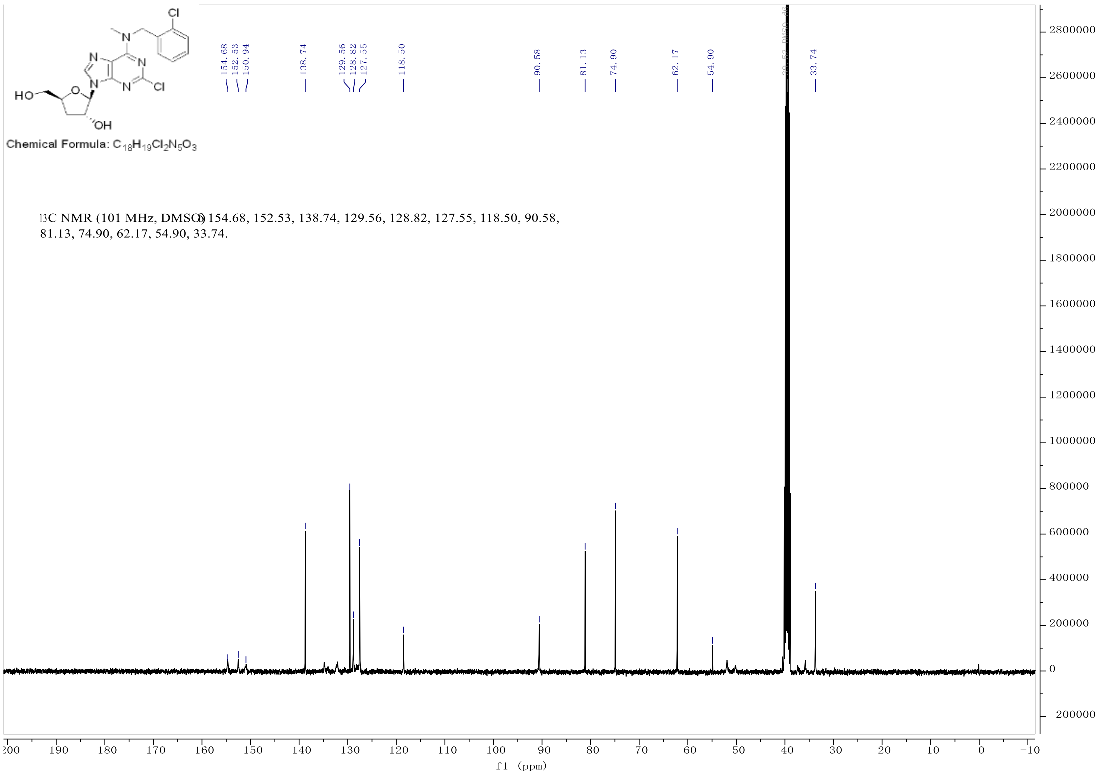

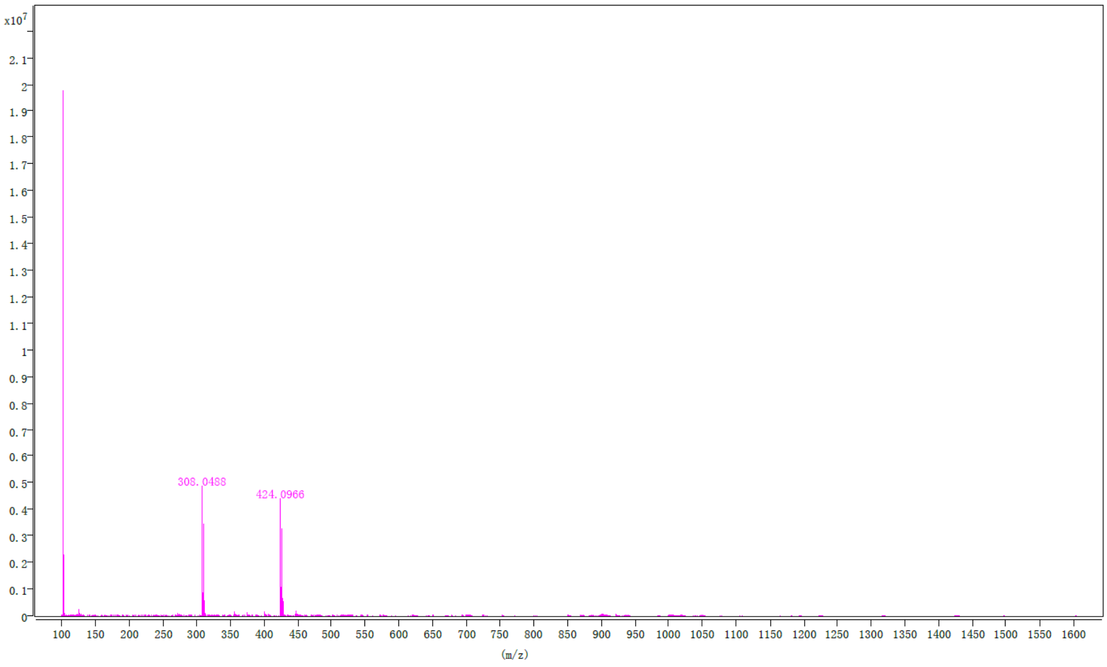

Figure S34. ^{1}**H NMR,** ^{13}**C NMR** and Mass spectra spectrum of compound **3a**

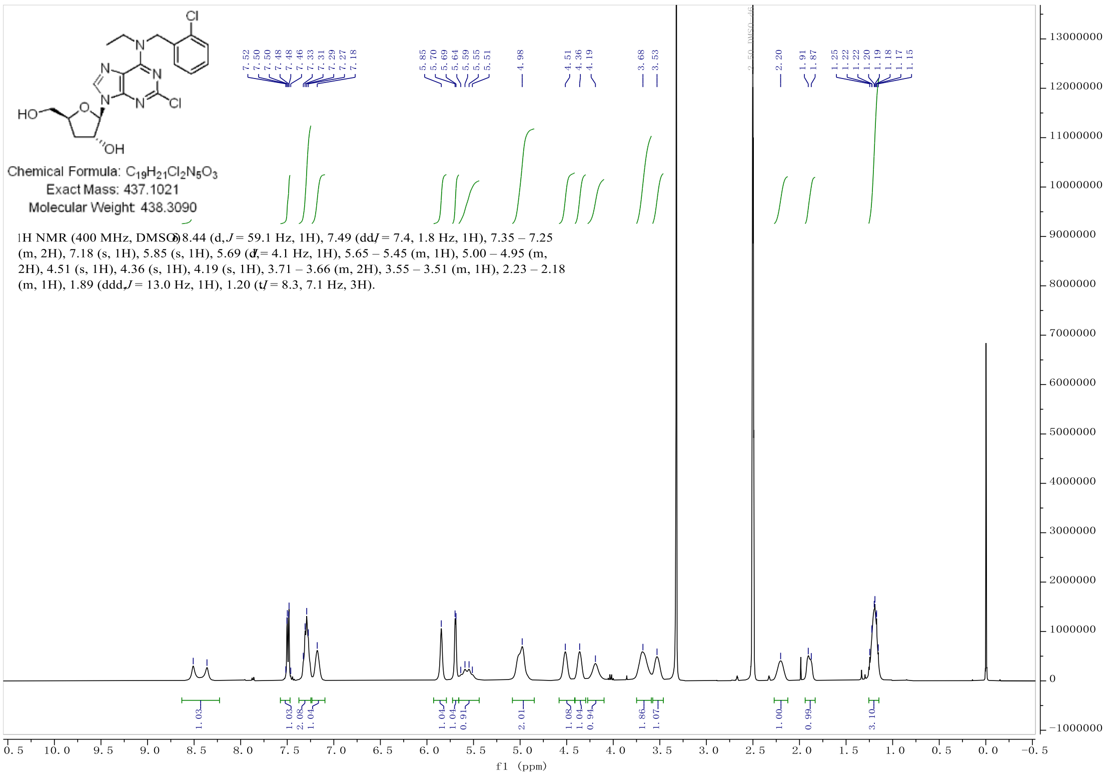

1H NMR (400 MHz, DMSO) δ 8.44 (d, J = 59.1 Hz, 1H), 7.49 (dd, J = 7.4, 1.8 Hz, 1H), 7.35 – 7.25 (m, 2H), 7.18 (s, 1H), 5.85 (s, 1H), 5.69 (d, J = 4.1 Hz, 1H), 5.65 – 5.45 (m, 1H), 5.00 – 4.95 (m, 2H), 4.51 (s, 1H), 4.36 (s, 1H), 4.19 (s, 1H), 3.71 – 3.66 (m, 2H), 3.55 – 3.51 (m, 1H), 2.23 – 2.18 (m, 1H), 1.89 (ddd, J = 13.0 Hz, 1H), 1.20 (t, J = 8.3, 7.1 Hz, 3H).

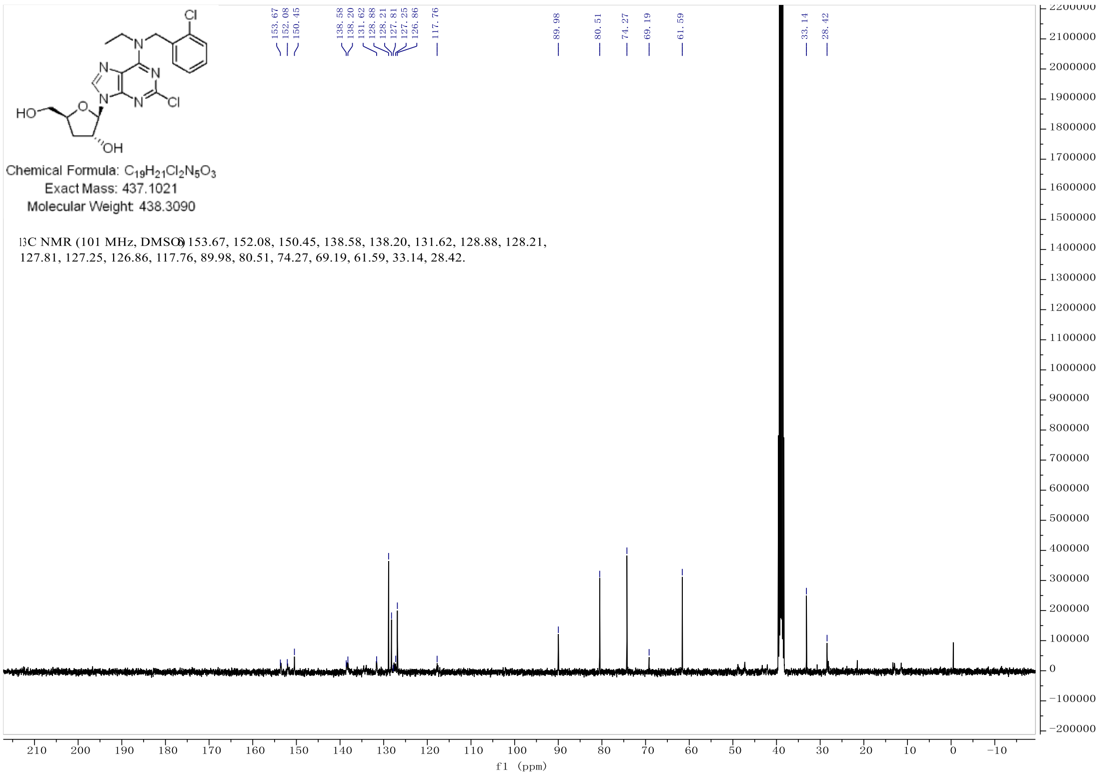

13C NMR (101 MHz, DMSO) δ 153.67, 152.08, 150.45, 138.58, 138.20, 131.62, 128.88, 128.21, 127.81, 127.25, 126.86, 117.76, 89.98, 80.51, 74.27, 69.19, 61.59, 33.14, 28.42.

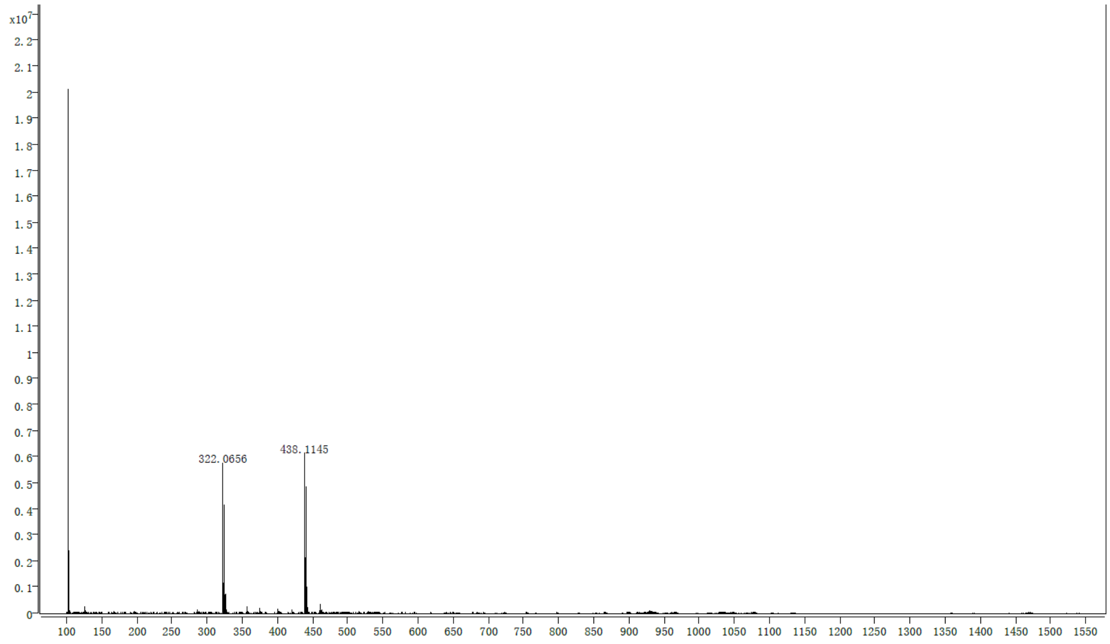

Figure S35. ^{1}H NMR，^{13}C NMR and Mass spectra spectrum of compound **3b**

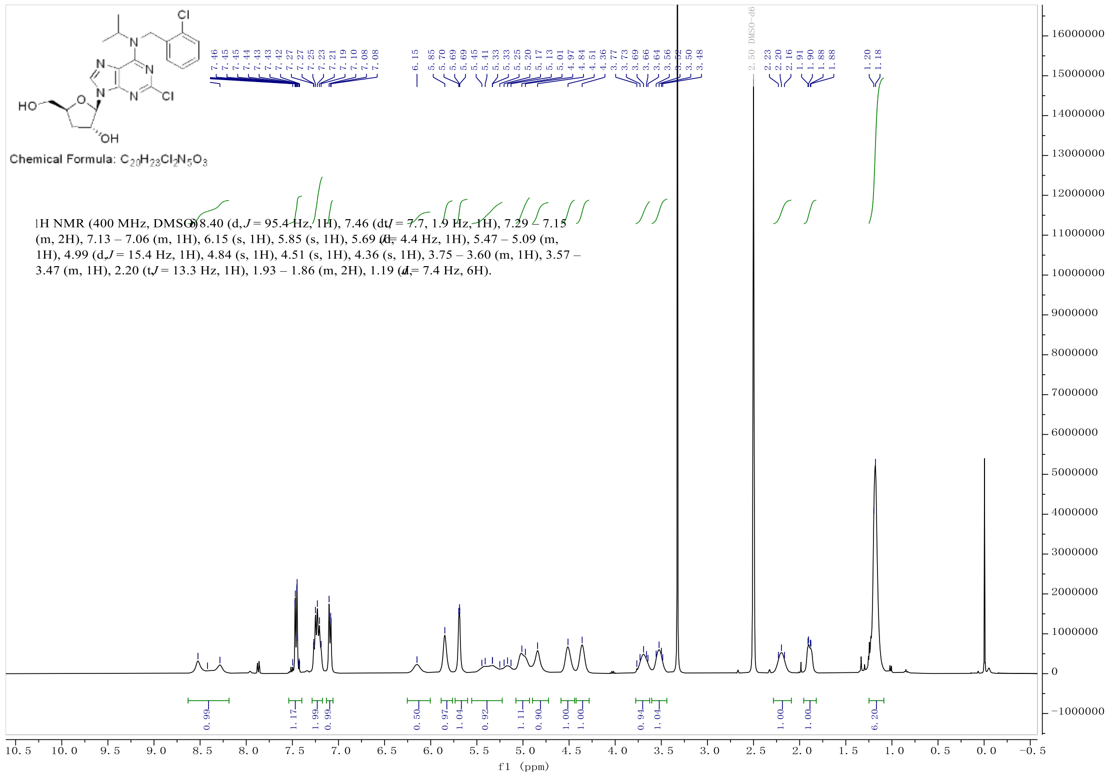

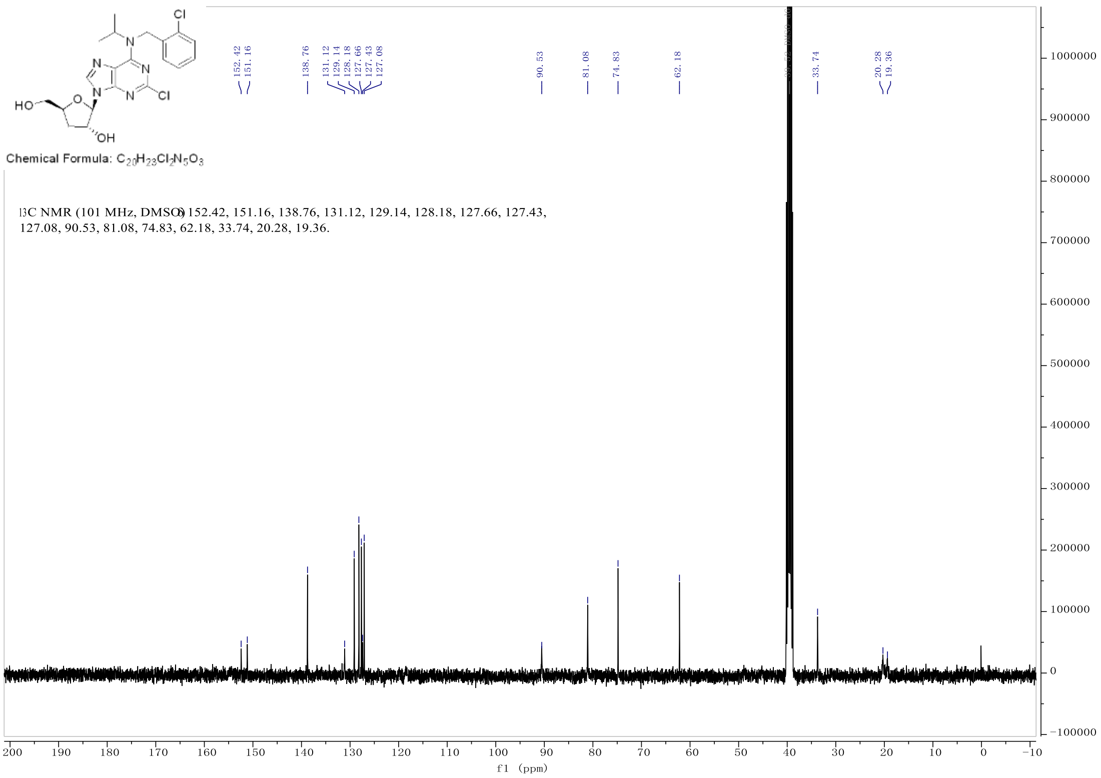

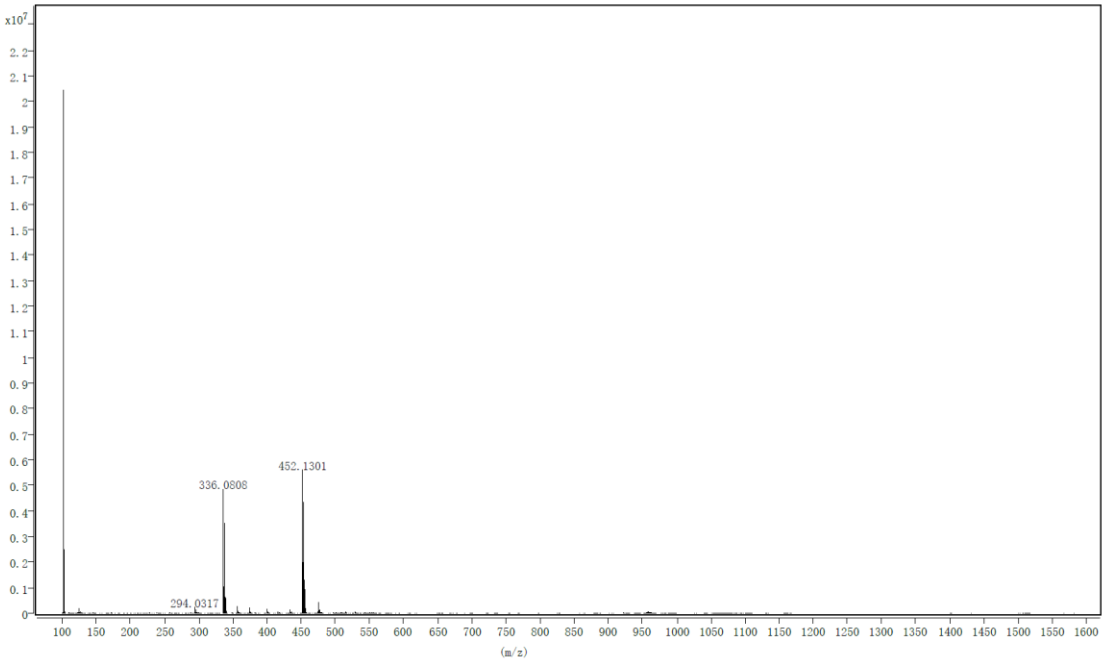

Figure S36. ^{1}**H NMR,** ^{13}**C NMR** and Mass spectra spectrum of compound **3c**

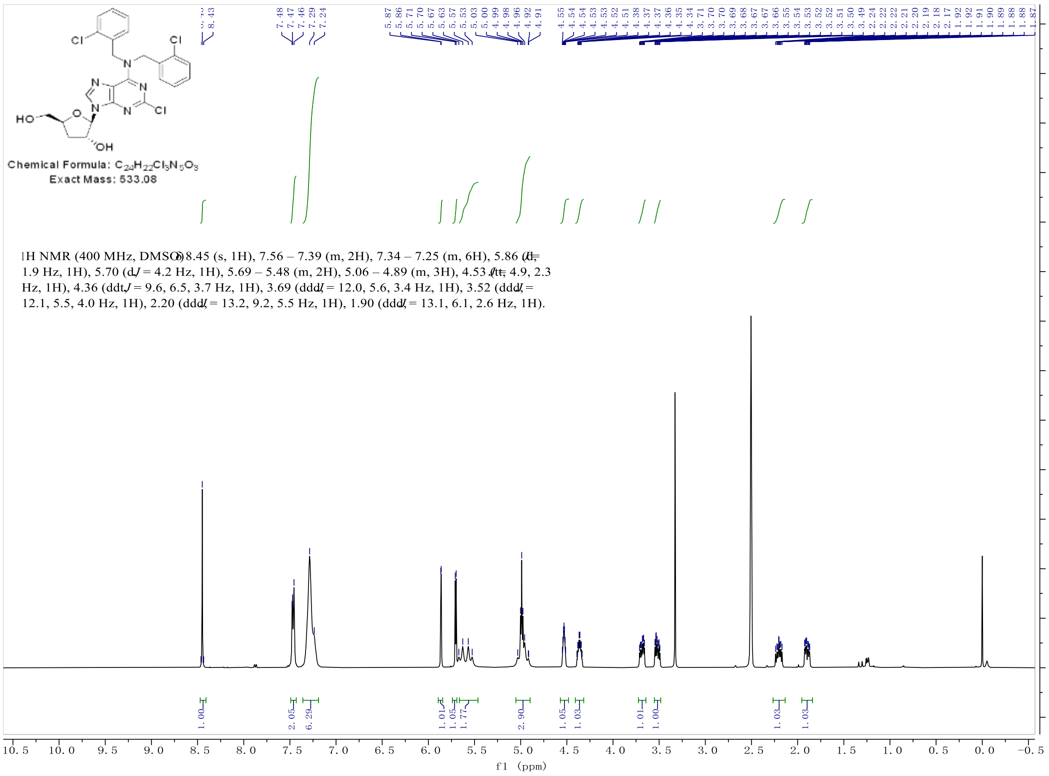
Cl
N
Cl
N
N
N
N
Cl
HO
O
OH
Chemical Formula: $C_{24}H_{22}Cl_3N_5O_3$
Exact Mass: 533.08
1H NMR (400 MHz, DMSO) δ 8.45 (s, 1H), 7.56 – 7.39 (m, 2H), 7.34 – 7.25 (m, 6H), 5.86 (d, J = 1.9 Hz, 1H), 5.70 (d, J = 4.2 Hz, 1H), 5.69 – 5.48 (m, 2H), 5.06 – 4.89 (m, 3H), 4.53 (tt, J = 4.9, 2.3 Hz, 1H), 4.36 (ddt, J = 9.6, 6.5, 3.7 Hz, 1H), 3.69 (ddd, J = 12.0, 5.6, 3.4 Hz, 1H), 3.52 (ddd, J = 12.1, 5.5, 4.0 Hz, 1H), 2.20 (ddd, J = 13.2, 9.2, 5.5 Hz, 1H), 1.90 (ddd, J = 13.1, 6.1, 2.6 Hz, 1H).
f1 (ppm)

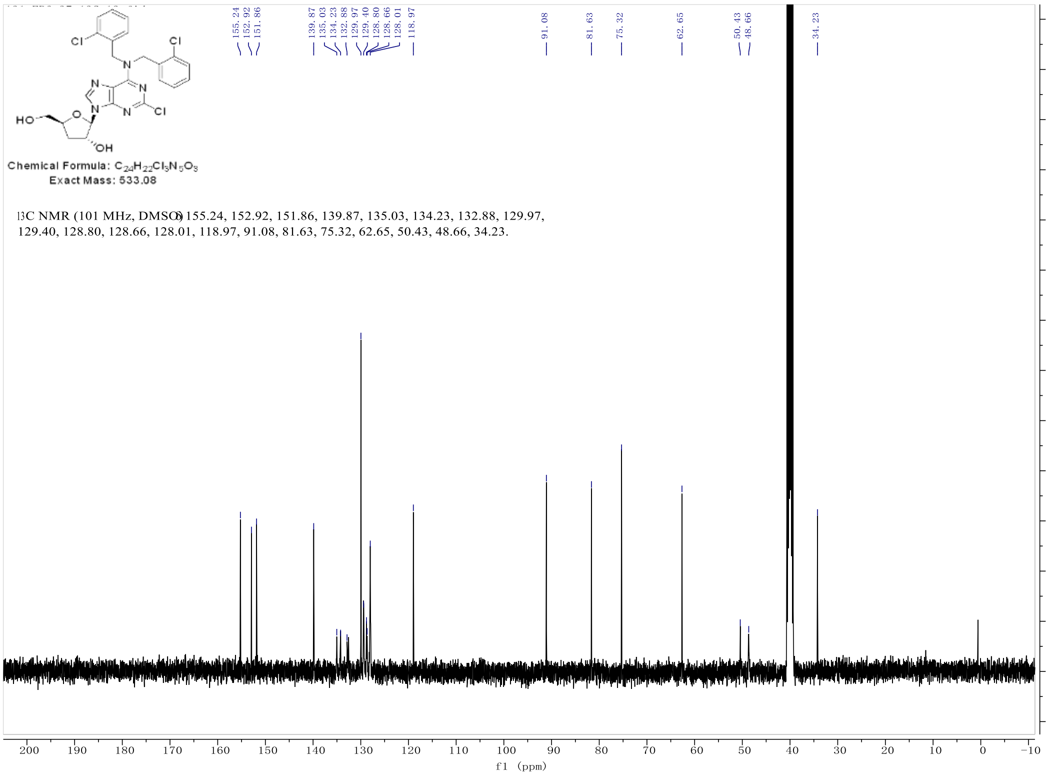
Chemical Formula: $C_{24}H_{22}Cl_3N_5O_3$
Exact Mass: 533.08
13C NMR (101 MHz, DMSO) δ 155.24, 152.92, 151.86, 139.87, 135.03, 134.23, 132.88, 129.97, 129.40, 128.80, 128.66, 128.01, 118.97, 91.08, 81.63, 75.32, 62.65, 50.43, 48.66, 34.23.
f1 (ppm)

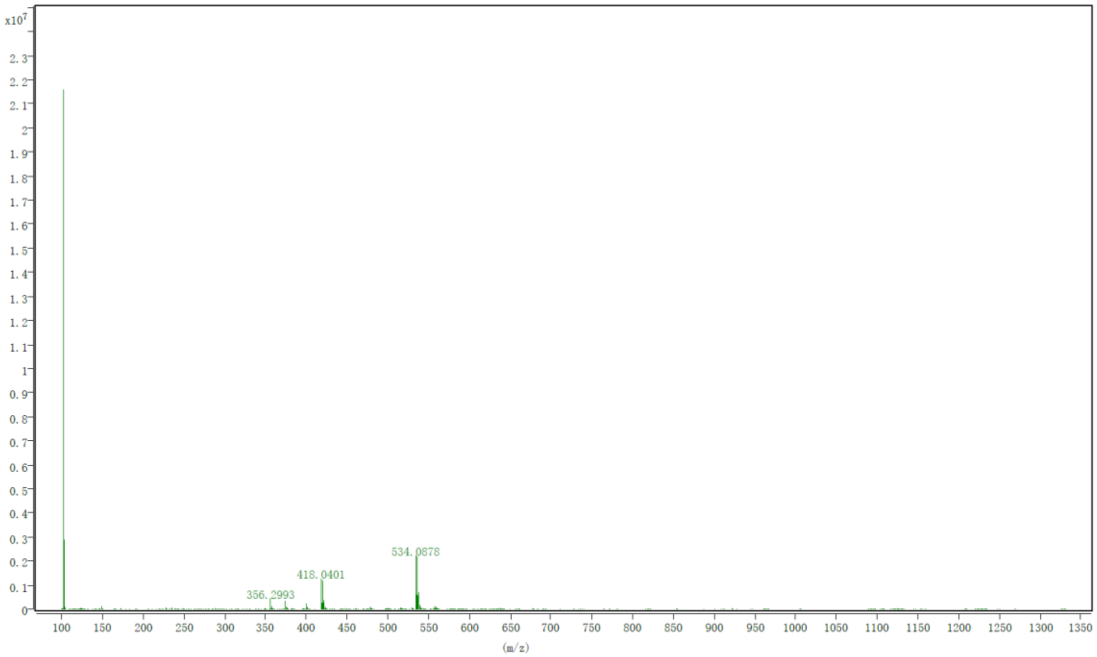

Figure S37. ^{1}**H NMR,** ^{13}**C NMR** and Mass spectra spectrum of compound **3d**
